# Preclinical small molecule WEHI-7326 overcomes drug resistance and elicits response in patient-derived xenograft models of human treatment-refractory tumors

**DOI:** 10.1038/s41419-020-03269-0

**Published:** 2021-03-12

**Authors:** Christoph Grohmann, Francesca Walker, Mark Devlin, Meng-Xiao Luo, Anderly C. Chüeh, Judy Doherty, François Vaillant, Gwo-Yaw Ho, Matthew J. Wakefield, Clare E. Weeden, Alvin Kamili, Jayne Murray, Sela T. Po’uha, Janet Weinstock, Serena R. Kane, Maree C. Faux, Esmee Broekhuizen, Ye Zheng, Kristy Shield-Artin, Nadia J. Kershaw, Chin Wee Tan, Helen M. Witchard, Gregor Ebert, Susan A. Charman, Ian Street, Maria Kavallaris, Michelle Haber, Jamie I. Fletcher, Marie-Liesse Asselin-Labat, Clare L. Scott, Jane E. Visvader, Geoffrey J. Lindeman, Keith G. Watson, Antony W. Burgess, Guillaume Lessene

**Affiliations:** 1 Walter and Eliza Hall Institute, Parkville, VIC 3052 Australia; 2 The University of Melbourne, Department of Medical Biology, Parkville, VIC 3050 Australia; 3 Ludwig Institute for Cancer Research, Melbourne, VIC 3000 Australia; 4 Peter MacCallum Cancer Centre, Victorian Comprehensive Cancer Centre building, Melbourne, 3000 Australia; 5 Cancer Therapeutics CRC, Melbourne, VIC 3000 Australia; 6 The University of Melbourne, Department of Obstetrics and Gynaecology, Parkville, VIC 3050 Australia; 7 Children’s Cancer Institute, Lowy Cancer Research Centre, UNSW, Sydney, NSW 2052 Australia; 8 School of Women’s and Children’s Health, UNSW, Sydney, NSW 2052 Australia; 9 Centre for Drug Candidate Optimisation, Monash Institute of Pharmaceutical Sciences, Monash University, Clayton, VIC 3052 Australia; 10 ARC Centre of Excellence in Convergent Bionano Science and Technology, Australian Centre for Nanomedicine, UNSW, Sydney, NSW 2052 Australia; 11 The University of Melbourne, Department of Medicine, Parkville, VIC 3000 Australia; 12 The University of Melbourne, Department of Pharmacology and Therapeutics, Parkville, VIC 3050 Australia

**Keywords:** Breast cancer, Cancer models, Cancer therapeutic resistance, Drug development, Mitosis

## Abstract

Targeting cell division by chemotherapy is a highly effective strategy to treat a wide range of cancers. However, there are limitations of many standard-of-care chemotherapies: undesirable drug toxicity, side-effects, resistance and high cost. New small molecules which kill a wide range of cancer subtypes, with good therapeutic window in vivo, have the potential to complement the current arsenal of anti-cancer agents and deliver improved safety profiles for cancer patients. We describe results with a new anti-cancer small molecule, WEHI-7326, which causes cell cycle arrest in G2/M, cell death in vitro, and displays efficacious anti-tumor activity in vivo. WEHI-7326 induces cell death in a broad range of cancer cell lines, including taxane-resistant cells, and inhibits growth of human colon, brain, lung, prostate and breast tumors in mice xenografts. Importantly, the compound elicits tumor responses as a single agent in patient-derived xenografts of clinically aggressive, treatment-refractory neuroblastoma, breast, lung and ovarian cancer. In combination with standard-of-care, WEHI-7326 induces a remarkable complete response in a mouse model of high-risk neuroblastoma. WEHI-7326 is mechanistically distinct from known microtubule-targeting agents and blocks cells early in mitosis to inhibit cell division, ultimately leading to apoptotic cell death. The compound is simple to produce and possesses favorable pharmacokinetic and toxicity profiles in rodents. It represents a novel class of anti-cancer therapeutics with excellent potential for further development due to the ease of synthesis, simple formulation, moderate side effects and potent in vivo activity. WEHI-7326 has the potential to complement current frontline anti-cancer drugs and to overcome drug resistance in a wide range of cancers.

## Introduction

For cancer therapy, the development of new medicines with improved safety profiles that prolong survival rates of patients is a crucial goal^1^. Despite the promising decline in mortality rates for many types of malignancies to date, new drugs only contribute to a small percentage in this trend^2^, while systemic chemotherapy still forms the mainstay of cancer treatment^3^.

The unrestrained proliferation of cancer cells compared to normal cells has been used in the development of chemotherapy drugs to specifically inhibit cell cycle progression at different stages, in particular the mitotic phase^4^. Mitosis is a highly complex process primarily driven by the mitotic spindle microtubules. Failure to form a proper spindle leads to defects in chromosome separation, activation of the spindle checkpoint, cell cycle arrest and ultimately apoptosis. Some of the most effective anti-cancer drugs in clinical use are disrupting mitotic progression, and are commonly referred to as ‘anti-mitotics’^5,6^.

Microtubule-targeting agents (MTAs) have proven to be successful anti-mitotics in the clinic. Prominent examples of MTAs include the groups of taxanes^7–9^ and vinca alkaloids^10,11^, both being commonly administered as first-line treatment against a variety of malignancies^12^. However, two factors—namely resistance^13,14^ and toxicity—have limited their effectiveness in the clinic^4^. While the mechanisms of resistance are complex and only partially understood^15^, toxicity is linked to the MTAs’ effect on tubulin’s essential role in healthy cells during interphase^16^.

Thereby, the focus of current anti-cancer drug discovery efforts has shifted to drugs which target other components of the diverse and complex mitotic apparatus^17^ including kinases, e.g., Aurora kinases^18,19^, Polo kinases^20,21^ and other motor proteins or protein complexes involved, leading to mitotic arrest and cell death^22^.

Although these agents often circumvent neurotoxicity problems, their efficacy, in particular as single-agent therapeutics, has been limited^6^. Besides general drug resistance mechanisms^5,23^, mitotic slippage has been identified experimentally as a risk factor limiting the efficacy of these new anti-mitotic drugs^12^, highlighting the need for new treatment strategies not susceptible to mitotic slippage. Additionally, clinical trials have shown that combination with conventional chemotherapeutics results in higher response rates than targeted single-agent therapy^2^.

We have generated the anti-mitotic small molecule WEHI-7326, which shows broad in vivo efficacy in a range of tumor models. From a clinical perspective, WEHI-7326 is efficacious and well tolerated in vivo in patient-derived mouse xenograft (PDX) models of standard-of-care chemotherapy-resistant triple-negative breast cancer (TNBC)^24^, lung squamous cell carcinoma (LUSC)^25,26^, high-grade serous ovarian cancer (HGSOC)^27^, and high-risk neuroblastoma (HR-NB)^28^. When WEHI-7326 was administered in combination with standard-of-care relapse therapy, a mouse model of highly aggressive, *MYCN*-amplified neuroblastoma^29^ showed complete tumor growth inhibition. Our data indicate that WEHI-7326 (**1**) is a promising agent suitable for clinical evaluation in cancer therapy.

## Results

### Discovery and synthesis of a new class of anti-cancer molecules

**Fig. 1:**
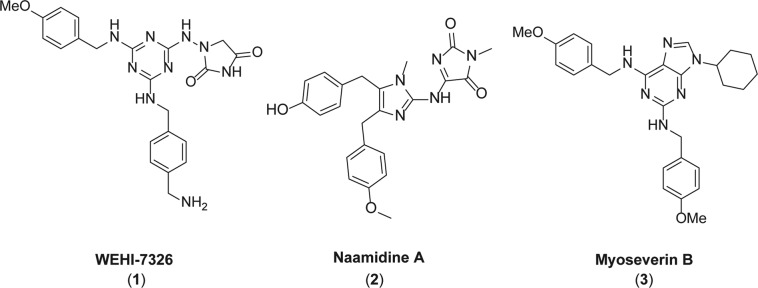
Discovery of WEHI-7326. Chemical structures of the anti-tumor compound WEHI-7326, natural product Naamidine A, and purine library compound Myoseverin B.

Inspired by the natural product naamidine A (**2**) and the cell cycle inhibitor myoseverin B (**3**) (Fig. 1)^30–33^, we synthesized a new series of compounds with potent anti-cancer activity (Supplementary Fig. S1a): compounds **1**, **5** and **6** inhibited proliferation of colon cancer (CRC) SW480 cell line in vitro at or below micromolar concentration (Supplementary Fig. S1b) and caused a strong G2/M cell cycle arrest (Supplementary Fig. S1c)^34^. Our lead compound WEHI-7326 exceeded potency of myoseverin B (**3**) by almost ten-fold (Supplementary Fig. S1b, d) and was considerably more potent than Naamidine A^33^. Suppression of cell growth was not due to immediate induction of cell death or apoptosis, as assessed by flow cytometry (Supplementary Fig. S1c), suggesting that those compounds inhibited cell proliferation specifically. Moreover, WEHI-7326 did not show any significant cytotoxic activity in vitro (IC_50_ > 40 μM) when assessed in HepG2 cells (Supplementary Fig. S1e).

### WEHI-7326 is a mitotic inhibitor and potential anticancer agent

**Fig. 2:**
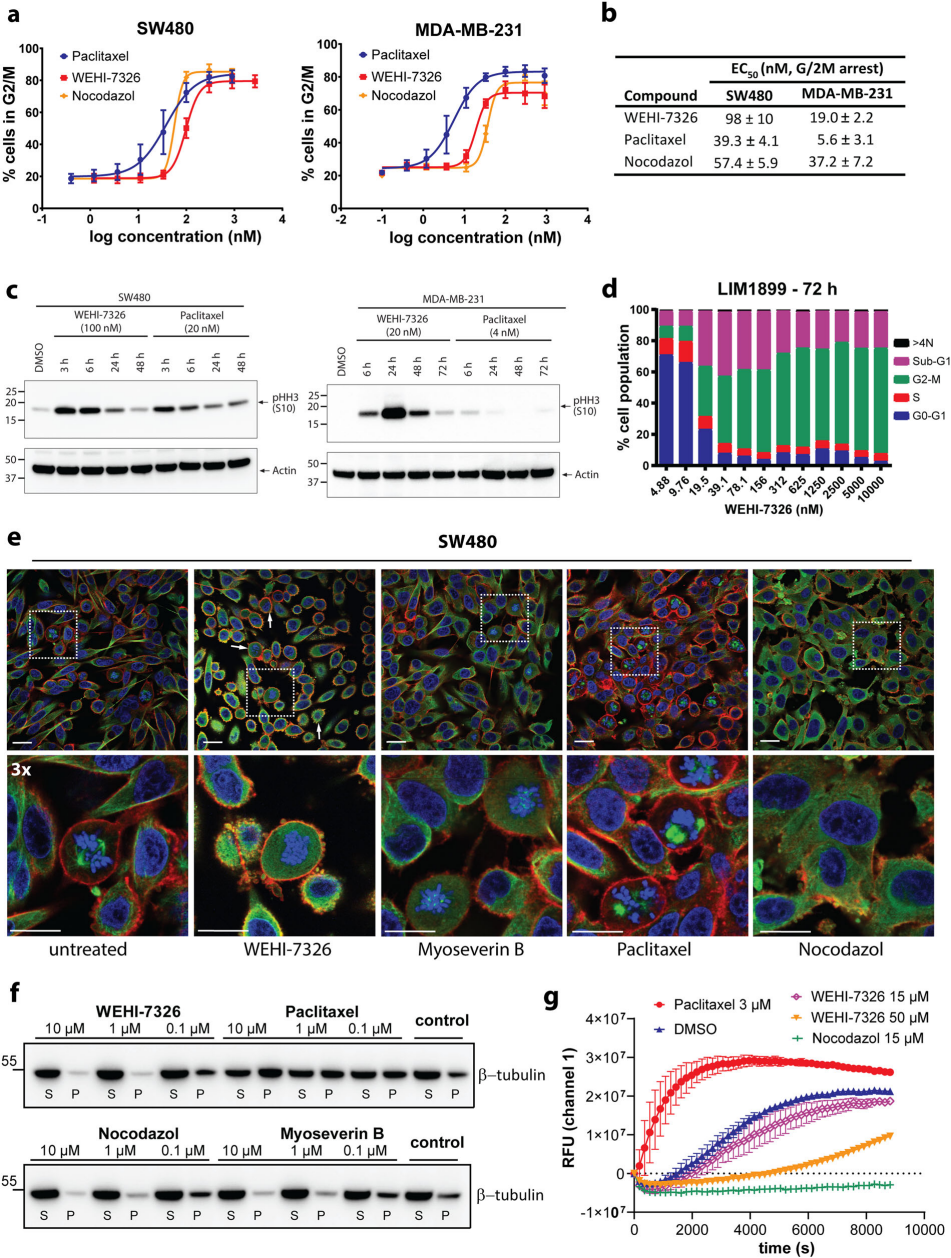
Antimitotic activity of WEHI-7326: cancer cell lines and effects on tubulin polymerization. **a** Dose-dependent antimitotic activity of WEHI-7326, nocodazol and taxol in tumor cell lines SW480 (colorectal) and MDA-MB-231 (breast). Data are presented as percentage of cells in G2/M after overnight incubation with increasing concentrations of inhibitors and PI staining followed by flow cytometry analysis. Mean average of 3 independent experiments ±SEM. **b** EC_50_ values (nM) for G2/M arrest on SW480 and MDA-MB-231 cell lines, given as mean average of 3 independent experiments ±SEM. **c** Western blot analysis of mitotic arrest in SW480 and MDA-MB-231 cells treated with EC_50_ concentrations of WEHI-7326 or Paclitaxel as marked by expression of phosho-S10 Histone H3 (p-HH3 S10), at different timepoints post treatment. Expression of *β*-actin provided as loading control. **d** Cell cycle analysis of LIM1899 cells 72 h post treatment with increasing concentrations of WEHI-7326 as measured by PI staining for DNA content followed by flow cytometry analysis. Sub-G1 represents apoptotic fractions and >4 N cell fractions of mitotic slippage. **e** Immunofluorescence confocal microscopy of tubulin polymerization in SW480 cells treated by various agents. Cells were incubated for 24 h with control (0.1% DMSO), nocodazole (1 μM), paclitaxel (1 μM), myoseverin B (1 μM) or WEHI-7326 (1 μM). Cells were fixed and stained with *β*-tubulin antibody (green) and DAPI (DNA; blue) and phalloidin (actin, red). Shown are single confocal sections and enlarged areas (3x) to highlight changes in microtubule network; scale bar = 20 μm. Representative images from 2 independent experiments. **f** Cellular tubulin polymerization assay in cells: SW480 cells were incubated overnight with paclitaxel, myoseverin B or WEHI-7326, then processed to detect fractions of polymerized (P) and soluble (S) tubulin by western blot analysis. Representative blot of 3 independent experiments. **g** Tubulin polymerization was assessed in vitro over time using a fluorescence-based tubulin polymerization assay (BK011P). Tubulin was incubated with control (DMSO), paclitaxel (3 μM), nocodazole (15 μM) and WEHI-7326 at indicated concentrations (in DMSO). Polymerization was measured by excitation at 360 nm and emission at 420 nm. Representative graph of 3 independent experiments performed in duplicates.

Typically, WEHI-7326 caused accumulation of cells in G2/M with nanomolar EC_50_ values in cancer cell lines, as assessed via flow cytometry (Fig. 2). Its effects on the cell cycle were comparable in potency to the widely used anti-mitotic drugs paclitaxel and nocodazole (Fig. 2a, b). Accordingly, WEHI-7326 inhibited cellular proliferation in the low nanomolar range on a broad range of tumor cell types (Table 1). In a panel of cancer cell lines, WEHI-7326’s anti-proliferative activity was comparable to nocodazole, over 10-fold higher than myoseverin B’s, and not as high as paclitaxel’s (Supplementary Fig. S2). When cells were treated with WEHI-7326, a rapid and strong mitotic arrest marked by phospho-Ser10 histone H3 (p-HH3) expression was observed (Fig. 2c). The expression of p-HH3 was dependent on the concentration of WEHI-7326 (Supplementary Fig. S3). Cell cycle analysis on LIM1899 CRC cell line showed a high percentage of cells in G2/M 72 h post-treatment, while no significant evidence of mitotic slippage (>4 N DNA content) but increased cell death was observed (sub-G1 population, as shown in Fig. 2d). Analysis *via* AnnexinV/PI flow cytometry and Western blotting for cleaved caspase-3 revealed a positive correlation between treatment with WEHI-7326 and induction of these apoptotic markers in cells (Supplementary Fig. S4). Live-cell imaging of SW480 cells treated with WEHI-7326 revealed membrane blebbing and morphological features of apoptotic death (see Supplementary Videos S1 and S2).

**Table 1:** Inhibition of cellular proliferation by WEHI-7326: a panel of cancer cell lines.

Cell line	Type of cancer	IC_50_ (nM)*
LoVo	Colon carcinoma	42.2 ± 9.9
SW480	Colon carcinoma	39.5 ± 4.1
LIM1899	Colon carcinoma	31.3 ± 2.6
LIM 2537	Colon carcinoma	58.9 ± 7.5
MDA-MB-231	Breast carcinoma	24.4 ± 3.1
PC3	Prostate carcinoma	48.9 ± 11.1
H1437	Non-small cell lung carcinoma	29.7 ± 10.8
U87MGΔ2–7	Glioblastoma expressing activated EGFR	6.1 ± 1.6
CEM	Leukemia, acute lymphoblastic	3.99 ± 0.04
CEM dEpoB30	Leukemia (taxane resistant, β-tubulin mutation)	1.67 ± 0.04
CEM dEpoB300	Leukemia (taxane resistant, β-tubulin mutation)	6.51 ± 0.08

*72 h viability assay using CellTiterGlo or Alamar Blue (leukemia cell lines) reagent. IC_50_ values are the mean of 3 independent experiments run in duplicates shown with SEM.

When compared to MTAs paclitaxel, nocodazole and myoseverin B using confocal immunofluorescence microscopy (Fig. 2e and Supplementary Fig. S5), WEHI-7326 caused arrest in an earlier stage of prometaphase, characterized by dissolution of the nuclear envelope and condensed chromosome. Similar to MTAs morphologically, WEHI-7326 treatment resulted in rounded cells. Unlike paclitaxel and other MTAs, polymerized tubulin structures or spindle formation were not observed with WEHI-7326 even at high concentration (1 μM, 10x EC_50_, as shown in Fig. 2e). Rather, cells were characterized by depolymerized tubulin (Supplementary Fig. S5). At lower concentration (100 nM) of WEHI-7326, the tubulin network started to dissolve, resembling tubulin depolymerizer nocodazole (Supplementary Fig. S5); however, chromosome bundling was observed with WEHI-7326 but not with nocodazole treatment.

Many mitotic inhibitors affect tubulin polymerization^30,35,36^. We therefore compared the effects of WEHI-7326 on tubulin stability in live SW480 cells and in vitro with three known MTAs: paclitaxel (tubulin polymerizer), nocodazole and myoseverin B (depolymerizers) (Fig. 2f, g)^35^. We observed no significant change in the ratio of polymerized to soluble tubulin after treatment with WEHI-7326 at 100 nM (IC_50_) compared with control cells (Fig. 2f). However, an increasing amount of soluble tubulin was observed at 10- to 100-fold higher concentrations, resembling the behavior of nocodazole and myoseverin B, but distinct from paclitaxel. Importantly, WEHI-7326 did not affect in vitro tubulin polymerization rates with purified tubulin at concentrations up to 15 μM compared to vehicle or MTA controls paclitaxel and nocodazol (Fig. 2g). However, a higher concentration (50 μM) of WEHI-7326 markedly slowed tubulin polymerization.

These results suggest that WEHI-7326 can affect microtubule dynamics. In contrast to nocodazole, myoseverins and other triazines^30,35,36^, the high doses required in our experiments to observe this effect indicated that a different mechanism-of-action for WEHI-7326 may underpin its potent activity at lower concentration.

Cells can acquire resistance to MTAs through mutation or overexpression of tubulin^14,37^. When tested on epothilone-resistant leukemia cell lines CEM-dEpoB30 and dEpoB300 that are cross-resistant to paclitaxel^38^, WEHI-7326 retained nanomolar potency (Table 1 and Supplementary Fig. S6). This suggests the mechanism-of-action driving WEHI-7326’s anti-proliferative activity is distinct from that of paclitaxel and other MTAs, bearing great potential benefits to patients with taxane-refractory or MTA treatment-resistant tumors.

### WEHI-7326 is well tolerated in mice and efficacious in tumor xenograft models

On the basis of our promising in vitro data, we assessed the in vivo efficacy and tolerability with dose escalation along with pharmacokinetic studies (Supplementary Fig. S7): WEHI-7326 can be easily formulated (Supplementary Fig. S7a) and possesses a favorable pharmacokinetic profile (Supplementary Fig. S4b–d) when delivered intravenously. Based on the expected low hepatic clearance detected in vitro and very low lipophilicity (Supplementary Fig. S7), the measured systemic clearance is likely due to renal elimination. There were no adverse reactions or compound-related side effects up to a dose of 30 mg/kg in Swiss outbred mice. A single-dose acute toxicity study in rats at 20 mg/kg revealed minimal and reversible differences in hematology and coagulation parameters 14 days post dose, with majority of macroscopic findings at necropsy and significant clinical pathology outcomes considered incidental and unrelated to treatment (Supplementary Tables S1–6). A follow-up non-GLP 14 day maximum tolerated dose study in female Sprague-Dawley rats defined 10 mg/kg (twice weekly, i.v.) as the maximum tolerated dose, whereas signs of toxicity were observed with repeat dosing at higher concentrations of 15 mg/kg or 30 mg/kg; 60 mg/kg was found to be acutely lethal (Supplementary Table S7).

**Fig. 3:**
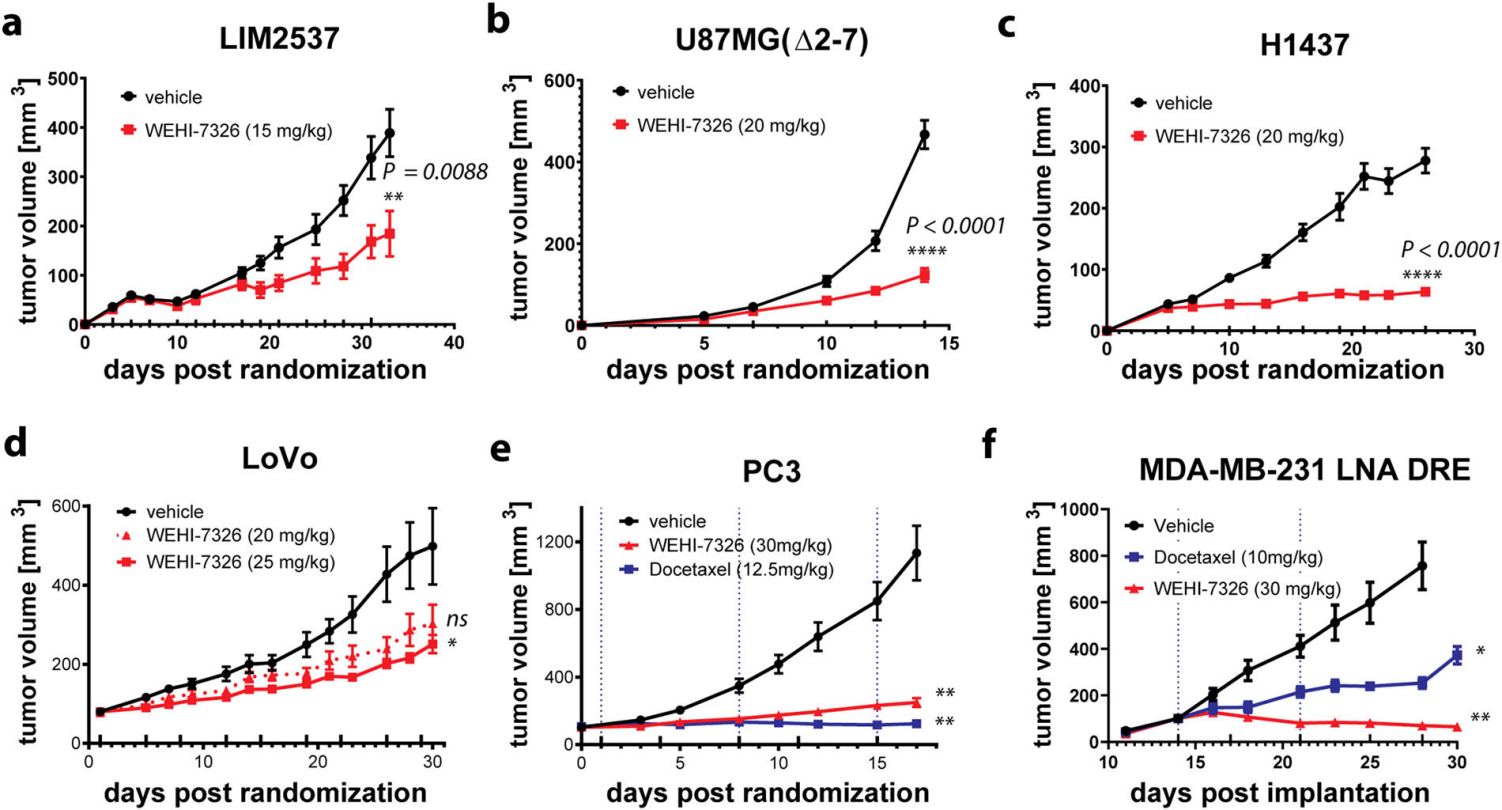
WEHI-7326 reduces the growth of multiple tumor types in xenograft models. Tumor growth curves of tumor xenografts in Balb/c nude mice. **a**–**c** LIM2537 (colon carcinoma), U87MG(Δ2–7) (glioblastoma) or H1437 (non-small cell lung carcinoma) were implanted subcutaneously in female mice. Treatment with vehicle or WEHI-7326 was initiated at day 5 and administered three times weekly *via* intraperitoneal injection. Tumor diameters were measured thrice weekly and used to calculate tumor volume. Data show mean±SEM (*n* = 8). **d** LoVo (colon carcinoma) in male mice. Treatment with vehicle or WEHI-7326 (biweekly) was administered *via* tail vein injection. Tumor diameters were measured twice weekly and used to calculate tumor volume. Data show mean±SEM (*n* = 5). **e** PC3 (human prostate carcinoma) in male mice. Treatment with vehicle (saline), Docetaxel (weekly) or WEHI-7326 (biweekly) was administered *via* tail vein injection. Tumor diameters were measured twice weekly and used to calculate tumor volume. Vertical line in left graph show docetaxel treatment. Data show mean ± SEM (*n* = 10). **f** MDA-MB-231 LNA DRE (docetaxel resistant breast cancer) in female mice. Treatment with control vehicle, docetaxel (weekly) or WEHI-7326 (biweekly) was administered *via* tail vein injection. Tumor diameters were measured thrice weekly and used to calculate tumor volume. Vertical line in left graph show docetaxel treatment. Data show mean ± SEM (*n* = 12). Upward ticks on x-axis depict drug administration points for WEHI-7326, dotted blue lines for docetaxel. *P*-values for differences in tumor volume (compared to vehicle) were generated via unpaired *t*-tests; *p* > 0.05 (ns), *p* < 0.05 (*), *p* < 0.01 (**) and *p* < 0.0001 (****).

The efficacy of WEHI-7326 in reducing the growth of ectopic xenograft tumors in nude mice was determined. WEHI-7326 was effective in reducing tumor growth in LIM2537, LoVo (colon carcinomas)^39^, U87MG-(Δ2–7) (recombinant glioblastoma)^40^ and H1437 (non-small cell lung carcinoma)^41^ tumors (Fig. 3). Effectiveness in the xenograft studies correlated with the tumor growth rates: WEHI-7326 was more effective in the fast growing and more aggressive U87MG-(Δ2–7) and H1437 tumors than in the comparably slower growing LIM2537 and LoVo tumor xenografts. Mice treated for up to 36 days with WEHI-7326 were healthy and displayed normal behavior and posture. The major side effects of WEHI-7326 administration were weight loss (reverted by supplemental nutrition, as shown in Supplementary Figs. S8a–d) and the appearance of reversible small lesions at the site of injection. Preliminary toxicology of mice treated with WEHI-7326 for 36 days showed no gross alterations of the major organs, except for enlargement of the spleen (H1437, as shown in Supplementary Fig. S9) consistent with extramedullary hemopoiesis. The efficacy results show a 60% tumor growth inhibition in the LoVo human colon carcinoma model following treatment with WEHI-7326 at 25 mg/kg (Fig. 3d) while the compound was well tolerated over the course of the experiment (~6% body weight loss at a dose of 25 mg/kg, 9 doses in total, as shown in Supplementary Fig. S8d), with no signs of increased liver or spleen weights (Supplementary Fig. S9d) 24 h after the last dose. *Post mortem* analysis of LIM2537 xenograft tumors revealed significant cell death in the WEHI-7326 treatment group (Supplementary Fig. S9g).

The efficacy of WEHI-7326 *vs* standard-of-care chemotherapy docetaxel were compared in a PC3 xenograft model of human prostate cancer in male nude mice. The results showed a strong tumor growth inhibition following treatment with either docetaxel or WEHI-7326 (~75% TGI at day 17 for WEHI-7326) (Fig. 3e and Supplementary Fig. S9e). Importantly, WEHI-7326 was better tolerated than docetaxel which induced toxicity after only three doses in this model: mice had to be culled one week after dose three as they reached a pre-determined toxicity ethical endpoint (Supplementary Fig. S8e). It is likely that cachexia observed in this model compounded the impact of docetaxel.

We next compared the effect of docetaxel and WEHI-7326 in vivo in an orthotopic MDA-MB-231 LNA-DRE xenograft model for human triple-negative breast cancer. This cell line was derived from a MDA-MB-231 LNA primary tumor which continued to grow in a mouse undergoing docetaxel treatment and was used as a taxane-resistant tumor model. Both docetaxel (10 mg/kg weekly, i.v.) and WEHI-7326 (30 mg/kg biweekly, i.v.) dosing resulted in strong tumor growth inhibition (docetaxel 70% TGI, WEHI-7326 105% TGI, as shown in Fig. 3f). Notably, WEHI-7326 caused tumor regression. Again, docetaxel showed significant toxicity and only two doses were administered before body weight loss and health of the mice required euthanasia at day 30, at which point the tumors had started to grow at the rate seen in the vehicle treated mice (Supplementary Figs. S8f and S9f). In contrast, the WEHI-7326-treated mice showed no signs of toxicity, appeared healthy and gained weight, thus a second experiment was conducted with a dose of 35 mg/kg biweekly, i.v. until day 44 (30 days treatment). Following cessation of WEHI-7326 treatment, four mice had to be culled at day 57 showing signs of tumor growth and body weight loss, but three mice were still in perfect health at day 93 with almost complete tumor regression (Supplementary Fig. S8g, h).

### WEHI-7326 activity in taxane-resistant PDX models of triple-negative breast cancer

**Fig. 4:**
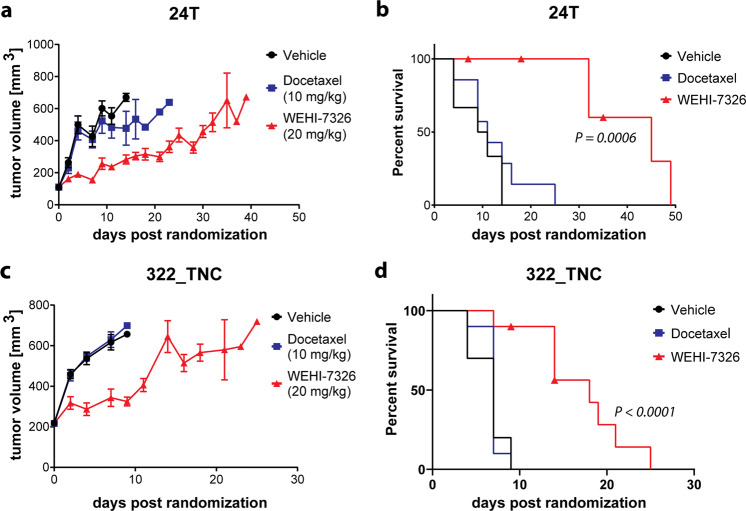
Efficacy of WEHI-7326 in taxane-refractory patient-derived xenograft mouse models of triple-negative breast cancer. **a**, **b** Tumor growth curve (**a**) and Kaplan–Meier survival plot (**b**) of 24 T TNBC PDX recipient female mice treated with either docetaxel (10 mg/kg, i.p.; once) or WEHI-7326 (20 mg/kg, i.v.; twice a week). p(docetaxel) = 0.3307 (ns), p(WEHI-7326) = 0.0006 (***) compared to vehicle control; *p*-values obtained through Log-rank (Mantel-Cox) test, *n* = 7 mice. Adverse effects in WEHI-7326 arm: 1 sick, 1 found dead, 1 with back leg paralysis. **c**, **d** Tumor growth curve (**c**) and Kaplan–Meier survival plot (**d**) of 322 T TNBC PDX recipient female mice treated with either docetaxel (10 mg/kg, i.p.; once) or WEHI-7326 (20 mg/kg, i.v.; twice a week). Vehicle mice were administered the vehicle for WEHI-7326. p(docetaxel) = 0.7974 (ns), p(WEHI-7326) <0.0001 (****) compared to vehicle control; p-values obtained through Log-rank (Mantel-Cox) test, *n* = 10 mice. Adverse effect in WEHI-7326 arm: 1 sick, 1 found dead.

We next expanded our pre-clinical studies of WEHI-7326 to validate these promising results in a clinically relevant, patient-derived xenograft model of human triple-negative breast cancer (TNBC). Two aggressive TNBC PDX models 24T^24^ and 322 T, highly refractory to treatment with standard-of-care taxanes, were chosen as possible clinical targets for WEHI-7326. NOD-SCID-IL2Rγc^–/–^ (NSG) female recipient mice of TNBC 24 T and 322 T tumors were randomized into three treatment arms and vehicle, docetaxel (10 mg/kg, i.p., once) or WEHI-7326 (20 mg/kg, i.v., biweekly) were administered for a maximum of three weeks, followed by an observational period. As expected, both taxane-resistant PDXs showed no difference in tumor growth or survival when treated with docetaxel compared to vehicle control (Fig. 4). However, both models responded well to single-agent therapy with WEHI-7326 without significant weight loss (Supplementary Fig. S10). WEHI-7326 significantly inhibited tumor growth in both models and consequently increased survival times compared to docetaxel (11 days vs 45 days for 24 T and 7 days vs 18 days for 322 T), even after cessation of treatment at day 21. The effects of short-time treatment with WEHI-7326 on the 24 T tumor cells was further studied in vivo and compared to docetaxel (Supplementary Fig. S11): at 16 h post single-dose treatment, a reduction in proliferation (Ki67 expression) and increased induction of apoptosis (cleaved/activated caspase-3) was found in both treatment groups but was significantly higher in the WEHI-7326 arm. No obvious findings of toxicity or peripheral neuropathy were observed upon post mortem analysis of spine and organs in this short-time treatment with WEHI-7326 (Supplementary Fig. S12).

### WEHI-7326 activity in treatment-resistant PDX models of lung cancer

**Fig. 5:**
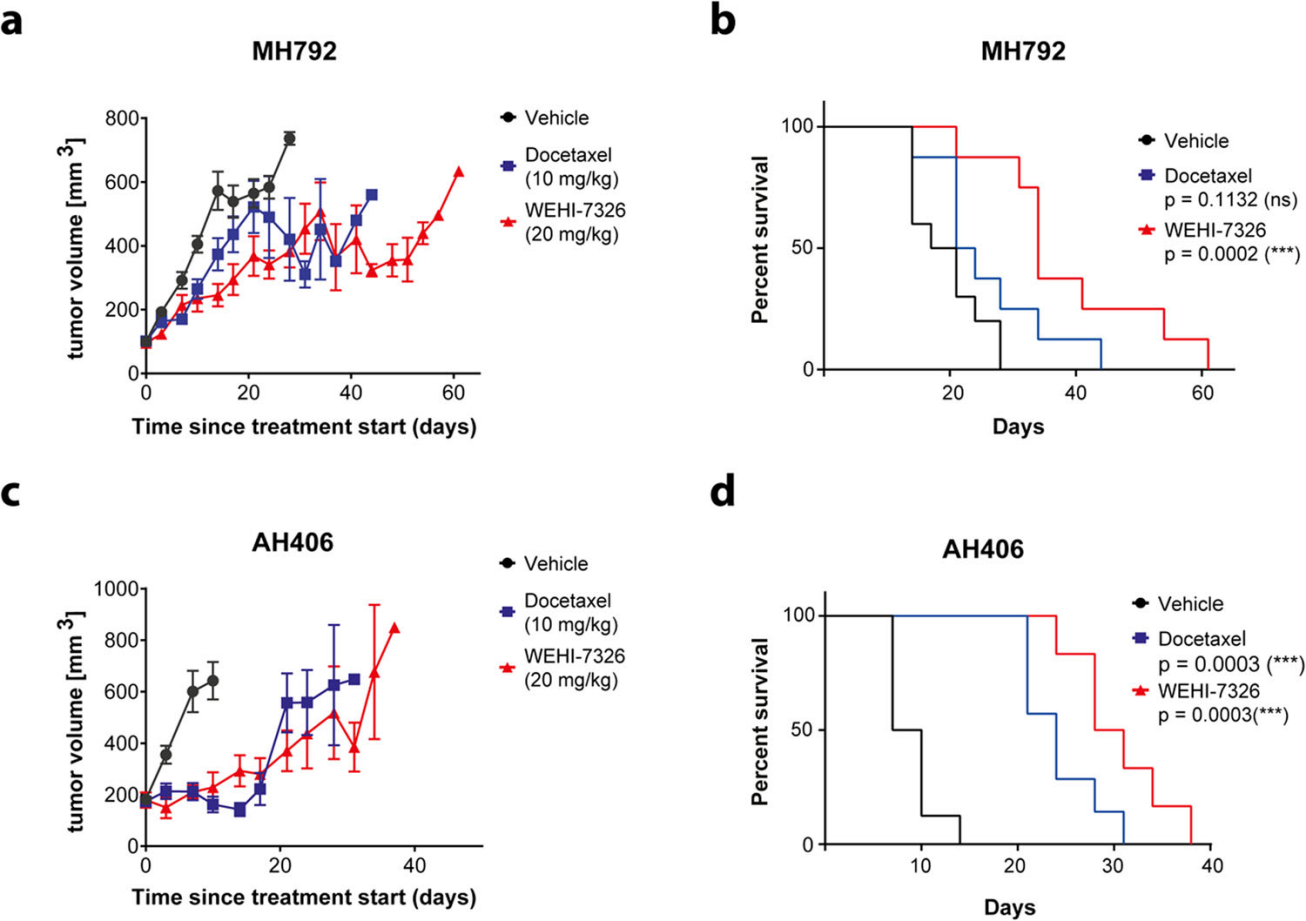
Efficacy of WEHI-7326 in cisplatin-refractory patient-derived xenograft mouse models of lung squamous cell carcinoma. **a**, **b** Tumor growth curve (**a**) and Kaplan–Meier survival plot (**b**) of MH792 LUSC PDX recipient male mice treated with either docetaxel (10 mg/kg, i.p.; once) or WEHI-7326 (20 mg/kg, i.v.; twice a week). *p*(docetaxel) = 0.1131 (ns), p(WEHI-7326) = 0.0002 (***) compared to vehicle control; p-values obtained through Log-rank (Mantel–Cox) test, *n* = 8–10 mice. Adverse effects in WEHI-7326 arm: 1 with hind limb paralysis after 4 weeks of treatment. **c**, **d** Tumor growth curve (**c**) and Kaplan–Meier survival plot (**d**) of AH406 LUSC PDX recipient male mice treated with either docetaxel (10 mg/kg, i.p.; once) or WEHI-7326 (20 mg/kg, i.v.; twice a week). Vehicle mice were administered the vehicle for WEHI-7326. p(docetaxel) = 0.0003 (***), p(WEHI-7326) = 0.0003 (***) compared to vehicle control; p-values obtained through Log-rank (Mantel-Cox) test, *n* = 6–8 mice.

WEHI-7326 was evaluated in a PDX model of human lung squamous cell carcinoma resistant to standard chemotherapy^25,26^. First-line therapy of these cancers involves treatment with platinum-based therapy followed by docetaxel or pemetrexed in platinum-resistant cancers. Two PDX models resistant to cisplatin therapy^25,26^ (Supplementary Fig. S13) were treated with either WEHI-7326 (20 mg/kg; i.v.; biweekly), docetaxel, or vehicle control for a period of four weeks. Both PDX models responded to WEHI-7326 with prolonged survival of animals compared to vehicle control or docetaxel for MH792 (Fig. 5). The median survival of the mice with MH792 tumors treated with WEHI-7326 was 34 days compared to 19 days with vehicle alone or 22 days with docetaxel. For the mice with AH406 tumors, treatment with WEHI-7326 showed a slight but significant (*p* = 0.05) improved survival over the docetaxel treatment group with a median survival of 29 days (WEHI-7326) versus 24 days (docetaxel). WEHI-7326 was well tolerated with little toxicity reported during the course of treatment and no change in animal body weight.

### High grade serous ovarian cancer PDX models respond to WEHI-7326

The in vivo efficacy and tolerability of WEHI-7326 was evaluated in a larger cohort of well annotated PDX models of high grade serous ovarian cancer (HGSOC) with defined in vivo responses to a platinum-based chemotherapy, cisplatin^42^. Despite good initial response to platinum-based chemotherapy, inevitably nearly all HGSOC will develop platinum resistance^43^. The PDX cohort consisted of four chemotherapy-naïve and three post-chemotherapy PDX models, which were generated from viable tumor fragments engrafted subcutaneously on the flank of NSG mice and expanded for treatment. The in vivo responses to platinum of each PDX model varies from sensitive, resistant to refractory, in keeping with the original patient’s response to chemotherapy as previously defined^27^ (Supplementary Figs. S14 and S15).

**Fig. 6:**
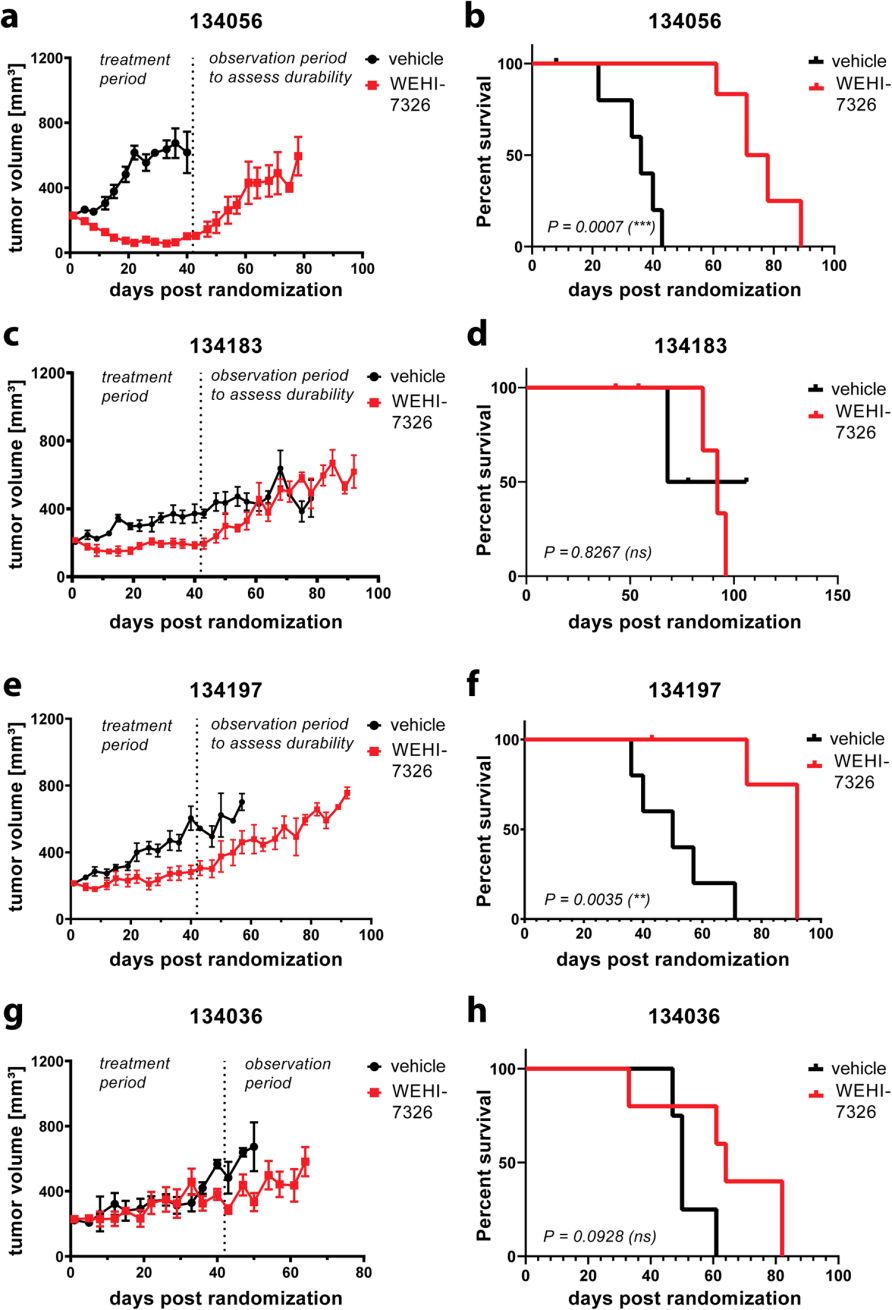
Response of chemo-naïve and chemo-resistant human high-grade serous ovarian cancer to WEHI-7326 in a PDX mouse model. PDX models of HG-SOC in recipient female mice: tumor growth curves and corresponding Kaplan–Meier survival plots for (**a**, **b**) chemo-naïve PDX tumor line 134056 (BRCA2 mutant), (**c**, **d**) the chemo-naïve PDX tumor line 134183 (Rad51C methylated), (**e**, **f**) 134197 (genotype unknown, platinum resistant PDX) and (**g**, **h**) 134036 (C5 PDX, platinum refractory). Each model was treated for 6 weeks with either vehicle or WEHI-7326 (20 mg/kg; twice a week) *via* tail vein injection, followed by an observational period. Ethical endpoint was tumor size >700 mm^3^; when mice had to be culled not due to tumor size (illness, weight loss or found dead) data from these mice were removed from the experiment. Displayed are *p*-values compared to vehicle obtained through Log-rank (Mantel–Cox) test.

WEHI-7326 (20 mg/kg i.v., biweekly) was administered for 6 weeks, followed by an observational period. Within the group of chemo-naïve PDX models - 134056 (gBRCA1 mutant), 134183 (RAD51C methylated), 134197 (C5 Tothill molecular subtype with HMGA2, MYCN and Lin28b expression) and 134036 (C5 Tothill molecular subtype) – 2 of 4 PDX showed statistically significant responses to WEHI-7326 (Fig. 6). In the platinum-sensitive 134056 PDX model, WEHI-7326 treatment resulted in an improvement of both time to progressive disease (PD) from 14 to 56 days and doubling of median time to harvest (TTH) from 36 to 78 days (p < 0.001) with evidence of tumor regression seen in all treated mice (Fig. 6a, b). In the cisplatin-resistant 134197 PDX model, WEHI-7326 treatment resulted in prolonged stabilization of tumor growth with no evidence of tumor regression leading to prolongation of time to PD from 11 to 35 days and improvement of median TTH from 50 to 92 days (*p* = 0.0033, as shown in Fig. 6e, f). In comparison to cisplatin, WEHI-7326 in vivo responses were inferior due to this PDX’s sensitivity to the platinum agent (Supplementary Fig. S14a). In the platinum-sensitive 134183 PDX model, WEHI-7326 treatment resulted in significant stabilization of disease leading to the improvement in the time to PD from 11 to 53 days but progression of disease was observed immediately upon cessation of treatment as demonstrated by lack of improvement of median TTH (Fig. 6c, d). Lastly, the 134036 C5 platinum refractory PDX was completely unresponsive to WEHI-7326. In all PDX models tested, WEHI-7326 was well tolerated over the 6 weeks treatment regimen and only minimal body weight losses were observed during this period (Supplementary Fig. S14c).

In the post-taxane treated and cisplatin-refractory PDX models, that were unresponsive to most conventional ovarian cancer therapies (PDX 134111, 134169 and 34931), WEHI-7326 treatment was also ineffective (Supplementary Fig. S15). In the 134111 (CCNE1 58x amplification), there was a trend towards improved outcome with WEHI-7326 treatment, however, due to individual outliers in the treatment arm, this failed to reach statistical significance. Both 134169^44^ and 34931 PDXs failed to respond to WEHI-7326. Interestingly, we observed very short-lived tumor regression in most tumors treated of the 34931 PDX model, leading to a small improvement in time to PD but no improvement in median TTH, as the tumor regression was not sustained.

### WEHI-7326 prolongs survival in mouse models of high-risk neuroblastoma

**Fig. 7:**
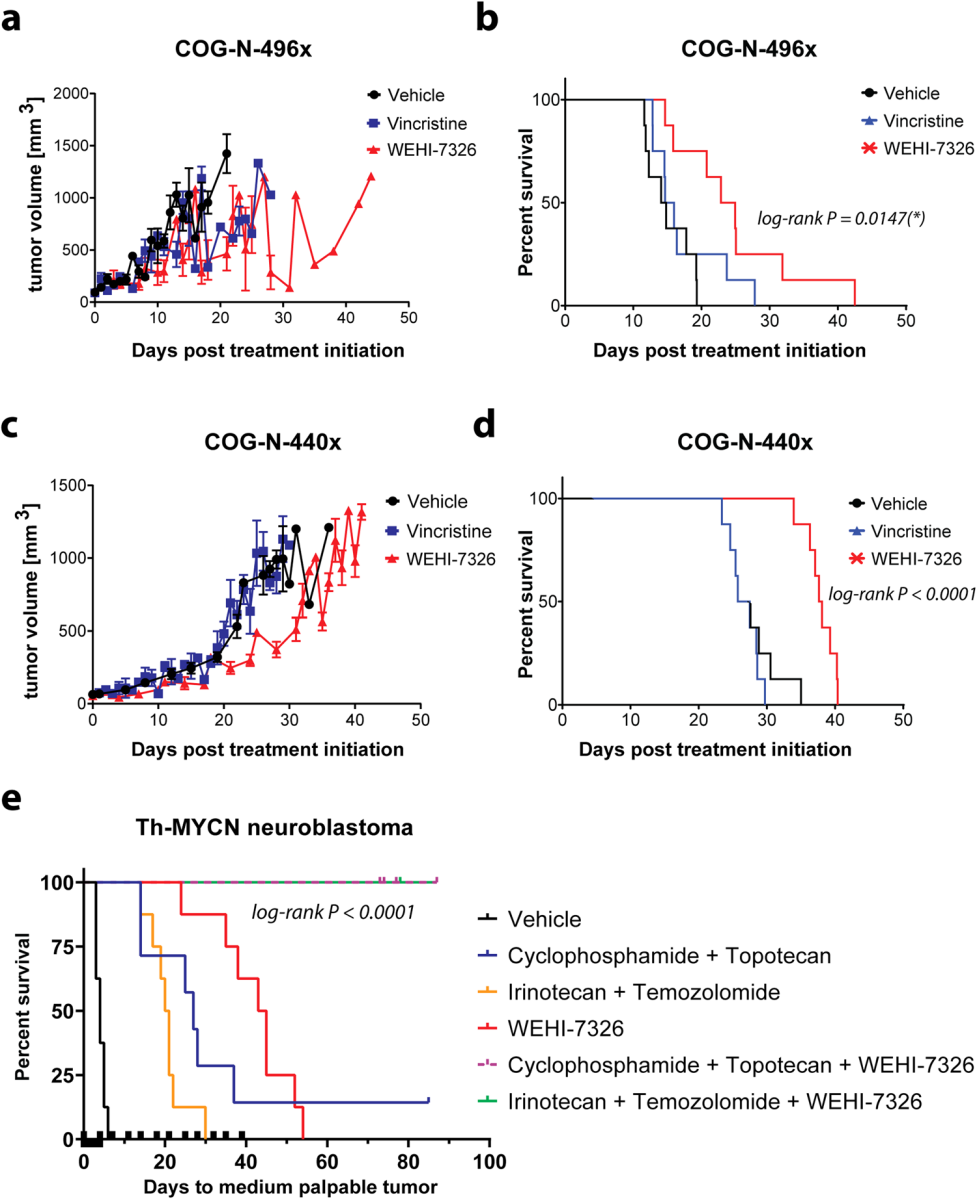
WEHI-7326 prolongs survival in mouse models of high-risk neuroblastoma and leads to complete tumor regression when used in combination with standard-of-care relapse therapies. Tumor growth curve (**a**) and Kaplan–Meier survival plot (**b**) of relapsed HR NB PDX model COG-N-496x treated with either vincristine (0.5 mg/kg, i.p.; once) or WEHI-7326 (20 mg/kg, i.v.; twice a week). *p*(vincristine) = 0.3741 (ns), p(WEHI-7326) = 0.0023 (**) compared to vehicle control; p-values obtained through Log-rank (Mantel-Cox) test, *n* = 8 mice. Tumor growth curve (**c**) and Kaplan–Meier survival plot (**d**) of refractory HR NB PDX COG-N-440x. *p*(vincristine) = 0.3602 (ns), *p*(WEHI-7326) <0.0001 (****) compared to vehicle control; *p*-values obtained through Log-rank (Mantel-Cox) test, *n* = 8 mice. **e** Kaplan–Meier survival plot of Th-MYCN transgenic model: spontaneous neuroblastoma driven by targeted expression of human MYCN in the developing mouse neural crest under control of the rat tyrosine hydroxylase (TH) promoter. Tumor growth was assessed by abdominal palpation and mice were euthanized at a medium palpable tumor of 1 cm^3^. Treatment schedules were vehicle control (saline); WEHI-7326 (20 mg/kg; twice weekly for 5 weeks; i.v.); cyclophosphamide (10 mg/kg, daily i.p.); topotecan (0.5 mg/kg, daily IP); irinotecan (2 mg/kg, daily, i.p.) and temozolomide (5 mg/kg, daily, i.p.). Mice that are surviving on this graph past 100 day showed no evidence of disease. Displayed *p*-value was obtained through Log-rank (Mantel-Cox) test.

We tested the efficacy of WEHI-7326 in two PDX models of relapsed or refractory high-risk neuroblastoma with MYCN amplification^28^, and in a Th-MYCN transgenic mouse model of high-risk neuroblastoma^29^. In the PDX model COG-N-496x^28^, a TP53 mutant model developed at diagnosis, WEHI-7326 (20 mg/kg i.v. twice weekly) slowed tumor growth and extended median survival from 15 days to 25 days (Fig. 7a, b). Similarly, in COG-N-440x^28^, a model established from a relapsed patient, WEHI-7326 extended median survival from 28 days to 39 days (Fig. 7c, d). For both models, the standard-of-care MTA vincristine (0.5 mg/kg i.p., once weekly) was entirely ineffective. In the Th-MYCN transgenic mouse model, where untreated tumors rapidly progress, WEHI-7326 (20 mg/kg i.v. twice weekly) extended median survival from 4 days to 44 days (Fig. 7e), outperforming the standard-of-care chemotherapy combinations of irinotecan-temozolomide (median survival 21 days) and cyclophosphamide-topotecan (median survival 27 days). As new agents for neuroblastoma therapy are typically trialed in relapsed or refractory patients in combination with standard relapse therapy, we assessed WEHI-7326 in combination with both irinotecan-temozolomide and with cyclophosphamide-topotecan. Remarkably, both triple drug combinations caused complete tumor regression, which was maintained up to 100 days with no evidence of disease at necroscopy. Both triple combinations were well tolerated (Supplementary Figs. S16 and S17).

## Discussion

In a search for anti-cancer drugs with new modes of action, we explored drug-like compounds combining aspects of the natural products naamidine A and myoseverin B. This led to the discovery of WEHI-7326, a potent G2/M blocker causing mitotic arrest and leading to apoptotic cell death. WEHI-7326 displays antiproliferative activity across a broad range of cancer cell lines comparable to clinically approved MTAs. Crucially in the context of a drug development program, WEHI-7326 is readily synthesized and exhibits very favorable in vitro and in vivo pharmacological properties.

Many common antimitotic drugs, such as taxanes, vinca alkaloids and epothilones, are MTAs and exert their anti-proliferative effect by altering microtubule dynamics, resulting in mitotic spindle defects and cell death^7–9^. Resistance to MTAs can develop through *β*-tubulin mutations or overexpression of *β*III-tubulin^14^. Although WEHI-7326 affected tubulin polymerization in vitro at high concentrations, this effect was much less pronounced than that observed with known MTAs with similar (paclitaxel) or even lower (nocodazole, myoseverin B) cellular potency. Notably, the morphology of WEHI-7326 treated cells differed from cells exposed to anti-cancer MTAs currently used in the clinic, suggesting a mitotic protein other than tubulin as target of this novel drug. In our preliminary experiments, WEHI-7326 remained efficacious in taxane and epothilone resistant cell lines dEpoB30 and dEpoB300, suggesting therapeutic potential use for WEHI-7326 in patients with acquired resistance to taxanes. At this stage, the precise molecular target(s) of WEHI-7326 remains unknown and further research into the mode-of-action is currently under way.

WEHI-7326 proved to be an effective in vivo single agent in reducing the growth of human breast, lung, prostate, brain and colon tumor xenografts in nude mice. Our pre-clinical data suggest that WEHI-7326 is a relatively safe drug, both as monotherapy and in combination with conventional cytotoxic agents. Some evidence of toxicity was observed in the nude mice xenograft models at the higher doses of WEHI-7326, but overall WEHI-7326 was well tolerated especially in comparison with anti-cancer drugs such as docetaxel. MTAs can adversely interfere with non-cancerous cells and lead to toxicities in patients (e.g., neuropathies, bone marrow suppression), thus lowering their therapeutic index. No evidence of bone marrow suppression was found in the toxicity studies with WEHI-7326. Peripheral neuropathy could not be observed after short time treatment but would need to be studied in longer treatment regimens. There was a noticeable and unexplained toxicity at longer treatments specifically in the TNBC PDX models and in one case of the LUSC PDX but not encountered with shorter treatments nor in the other PDX models tested with a similar treatment regimen of WEHI-7326. As a guide for further toxicology studies, a rat toxicity study determined a maximum tolerated dose of 10 mg/kg WEHI-7326 in this animal to be safe.

The efficacy and tolerability of WEHI-7326 in taxane-resistant triple-negative breast cancers were demonstrated in both cell line and patient derived xenograft models. The highly aggressive and docetaxel-resistant MDA-MB-231 LNA-DRE line xenograft regressed with WEHI-7326 treatment, while docetaxel treatment showed significant toxicity. Encouragingly, aggressive and docetaxel-refractory PDXs (24 T and 322 T), which represent clinically relevant and hard-to-treat TNBCs, responded very well to WEHI-7326 as a single agent. This finding highlights the therapeutic potential of WEHI-7326 for treatment of TNBC and it is expected that combination with standard-of-care therapies would prove highly beneficial. Additional xenograft experiments are required to investigate the role of WEHI-7326 in combination in other breast cancer subtypes such as ER + (with tamoxifen/fulvestrant or aromatase inhibitors) and HER-2 amplified (with Herceptin), but also in BRCA1 mutant BC (with PARP-inhibitor).

Despite the advances of immunotherapy in the treatment of lung cancer^45^, platinum-based doublet chemotherapy combined with radiotherapy remains the first line treatment for non-small cell lung cancer (NSCLC). However, disease progression is often seen in these patients, with resistance to therapy or relapse of the disease, resulting in a 5-year survival of only 15–30%^46^. Treatment with WEHI-7326 reduced tumor growth in vivo in two PDX models of NSCLC resistant to platinum therapy. Compared to docetaxel, treatment with WEHI-7326 also resulted in prolonged survival, further demonstrating the potential of this new compound in chemo-refractory tumors. Given the limited toxicity of WEHI-7326, further combination studies with targeting or immunotherapy drugs would need to be explored.

Combination therapy might also be important to improve the efficacy of WEHI-7326 for treatment of high-grade serous ovarian cancers in advanced stages. The HGSOC PDX models tested which had been derived from patients post-chemotherapy, did not respond to WEHI-7326 as a single agent. However, they represent especially highly refractory HGSOC in the clinical setting: 134111 is resistant to chemotherapy except MTAs; 134169 is resistant to both platinum and PARPi^44^; and 34931 is refractory to cisplatin. In two chemo-naïve HGSOC PDXs, an initial tumor response during WEHI-7326 treatment was observed, followed by progression of disease after cessation of treatment. The in vivo responses in chemo-naïve PDX models do not seem to correlate with platinum sensitivity, highlighting WEHI-7326 as a therapeutic alternative to platinum chemotherapy agents in certain types of HGSOC. Interestingly, the best HGSOC responder is 134056 PDX, which carries a gBRCA1 mutation and bears most molecular resemblance to TNBC. Therefore, the BRCA status of a tumor maybe a relevant biomarker for WEHI-7326 response and further strengthens the argument for combinatorial potential of WEHI-7326 with a PARP inhibitor in future studies. Olaparib has been shown to reduce the risk of ovarian cancer progression and death in carriers of gBRCA1/2 mutations by 70% and the addition of WEHI-7326 has the potential to improve outcomes for these women^47^.

WEHI-7326 was highly effective in a neuroblastoma transgenic mouse model when combined with two different standard-of-care regimens used to treat high-risk patients with relapsed or refractory disease. Both combinations elicited a complete response that was maintained for more than 100 days, with no evidence of disease at necroscopy. As combination with relapse chemotherapy regimens is a standard approach to assess new agents in this disease, studies of WEHI-7326 in additional models are warranted.

In conclusion, our pre-clinical data demonstrate that WEHI-7326 represents a new class of anti-cancer agents with good therapeutic window and developability. WEHI-7326 is both tolerable and efficacious in vivo and, notably, offers potential as alternative therapy for cancers hard to treat with current standard-of-care chemotherapy. Our ongoing research into the underlying mode-of-action of this compound aims to open new therapeutic avenues and opportunities in drug discovery for cancer treatment.

## Materials and methods

### Materials

Anti-mitotic drugs and reagents were purchased from Sigma-Aldrich (St. Louis, MS, USA): epothilone B (98%, E2656), paclitaxel (97%, T7191), docetaxel (97%, 01885), myoseverin B (97%, M3316), nocodazole (99%, M1404), DAPI (D9542), propidium iodide (P4864), RNAse (R6148), bovine serum albumin (A2153), DPX mounting medium (06522). Antibodies: Caspase-3/cleaved caspase-3 (CST9665, Cell Signaling Technology, MS, USA), phospho-Ser10 histone H3 (06-570, Merck Millipore, Darmstadt, Germany), anti-H2AX S139ph (CST9718, Cell Signaling Technology), β-actin antibody conjugated to HRP (ab49900), β-tubulin (ab6046), Alexa Fluor 488 (ab150077) from Abcam (Cambridge, UK), Alexa Fluor 647 (A28181, Invitrogen, Carslbad, CA, USA) and IRDye 800CW (926–32211) from Li-Cor (Lincoln, NB, US).

### Chemistry

For experimental details on synthesis of WEHI-7326 and related compounds, see supplemental information.

### IncuCyte® S3 live-cell analyses

Experimental details and videos of SW480-H2B-Scarlet-1 cells treated with WEHI-7326, see supplemental information.

### Cell lines and tissue culture

LIM1899 and LIM2537 (Ludwig Institute Melbourne)^39^, U87MG-Δ2–7^40^ and H1437^41^ cell lines were passaged in RPMI medium containing 10% fetal calf serum and supplemented with hydrocortisone (1 μg/ml), thioglycerol (0.01 μg/ml), and insulin (0.025 U/ml). SW480, MDA-MB-231 and LoVo cell lines (obtained from ATCC) were passaged in DME/F-12 medium with HEPES containing 10% FCS. PC3 (ATCC) cell lines were passaged in RPMI medium containing HEPES and 10% FCS and insulin (0.025 U/ml). MDA-MB-231-LNA cell line was derived from a metastatic lymph node of MDA-MB-231. MDA-MB-231-LNA-DRE cell line was derived from an MDA-MB-231-LNA primary tumor which continued to grow in a mouse undergoing docetaxel treatment. CCRF-CEM, CEM/dEpoB30 and CEM/dEpoB300 cells were maintained as previously described^38^.

All cell lines were confirmed mycoplasma negative prior to experimental use. Cell line authentication was performed by either Cell bank Australia (CEM lines) or AGRF Melbourne using short tandem repeat systems.

### Cell growth inhibition assays

Cells were plated at a density of between 500 to 5000 cells per well for adherent cell lines depending on doubling time, and 15,000 cells per well for lymphoblastic leukemia cell lines^48^, in a 96-well plate in 200 μl of growth media. Plates were incubated overnight at 37 °C before adding the inhibitors. Inhibitors were serially diluted from the stock solutions in complete medium and added to the cells. Incubation in the presence of inhibitors was continued for 72 days. Cell viability was assessed by the AlamarBlue (Invitrogen) or CellTiterBlue/Glo (Promega) method according to manufacturer’s instructions. The curve fits and IC_50_ values were generated in PRISM (GraphPad, San Diego, CA).

### Cell cycle analysis and Annexin V apoptosis assay

Cells in log growth were supplemented with fresh medium with either vehicle (control) or compound at the stated final concentrations. Cells were harvested after 16 h, fixed in ice-cold methanol/PBS (90:10) for 1 h at 4 °C, pelleted and resuspended in PBS containing propidium iodide (40 μg/ml) and RNAse (0.1 mg/ml) for the determination of DNA content. After 1 h incubation at room temperature in the dark, cells were analyzed on a FACScan (Becton and Dickinson) in CellQuest. The percentage of cells in each phase of the cell cycle was determined using ModFit software (Verity, http://www.vsh.com). Early and late apoptotic responses elicited by WEHI-7326 were assessed using Dead Cell Apoptosis Kit (ThermoFisher Scientific) with Annexin V FITC and PI following manufacturer’s instructions. Cells were incubated 24 h with 6-point, 4-fold titration of WEHI-7326, washed twice with cold 1X PBS, resuspended in 1X Annexin Binding Buffer, incubated with FITC Annexin V and PI for 15 mins at RT in the dark and subsequently analyzed by flow cytometry.

### Western blotting of phospho-Histone H3, caspase-3 and cleaved caspase-3

Cells were seeded and grown to 60% confluency on 6-well plates. For treatment, media was removed and cells were incubated with fresh media containing vehicle or drug at final concentration. Lysates were prepared in RIPA buffer (50 mmol/L Tris; pH 7.5, 150 mmol/L NaCl, 1% NP40, 0.5% sodium deoxycholate, 0.1% SDS) supplemented with a complete mini protease inhibitor cocktail tablet and phosphatase inhibitor PhosStop (Roche). Proteins were separated on Nu-PAGE precast gels (Invitrogen) and transferred onto nitrocellulose membranes using the iBlot transfer system (Invitrogen). Membranes were blocked in 5% skim milk in TTBS for 1 h at room temperature and probed with primary antibodies in 4% BSA in TTBS at 4 °C overnight. Immunoblots were washed with TTBS and probed with secondary antibody conjugated to horseradish peroxidase (Southern Biotech). Protein expression levels were visualized by Chemidoc Touch Imaging System (Bio-Rad) using enzyme-linked chemiluminescence (ECL; Immobilon Western, Millipore).

### High content imaging

Exponential growing MDA-MB-231 cells were cultured in 96-well black clear bottom microplates (#3603, Corning, NY USA). 24 h post cell seeding, cells were treated with 9-point, 4-fold titration of WEHI-7426 or nocodazol. Cells were blocked and permeabilized in 1X TBS with 5% goat serum and 0.3% Triton X100 for 1 h at RT. anti-H3S10ph and anti-H2AX S139ph antibodies were diluted in 1X TBS with 1% BSA and subsequently incubated with the cells after the removal of the blocking buffer overnight at 4 °C. Cells were then washed twice in 1X TBST for 10 mins each, followed by incubation using Alexa Fluor 488 or 647 conjugated secondary antibodies and Hoechst 33258 at 10 µM for 2 h at RT. Cells were washed three times in 1X TBST for 10 mins each and imaged using the OperaPhenix^TM^ High Content Screening System (PerkinElmer). Harmony High-Content Imaging and Analysis Software and Columbus Image Data Storage and Analysis System (Perkin Elmer) were used for data analysis.

### Immunofluorescence and confocal microscopy

Ibidi 8 well µ-slides (Ibidi cat# 80826) were coated with 0.01% poly-L-lysine. SW480 cells were plated onto the slides and cultured for 3 days or in case of synchronization, 2 days in complete medium before they were synchronized by a double thymidine block. Within an hour after release from the second block, vehicle or drugs were added at the stated final concentrations (0.1 or 1.0 μM). Cells were incubated with the drugs for 24 h, then fixed in 4% formaldehyde/PBS for 10 min at room temperature (RT), permeabilized with 0.2% Triton X-100 in PBS for 5 min at RT and incubated in PBS containing 0.2% Bovine Serum Albumin for 1 h at RT to block non-specific binding of antibodies. Cells were stained with the β-tubulin primary antibody (1 h at RT, 1:500), washed and incubated with AlexaFluor488-labeled secondary antibody (1:500) and Phalloidin (1:1000) for 1 h at RT, followed by a 5-min incubation with DAPI (0.1 µg/ml). Immunofluorescent staining of the cells was detected with an Olympus FV1000 Spectral Confocal attachment to an Olympus IX-81 microscope on a 60x water immersion lens. Images were collected using standard filter sets and laser lines (405 nm, 488 nm and 594 nm). The images were captured using Olympus FluoroView software (Version 1.7c). The images were imported into the ImageJ/Fiji^49^ software for processing and visualization using Bioformats^50^ plugins.

### Tubulin polymerization in cells

Cells incubated with control or with inhibitors were lysed in hypotonic buffer (1 mM MgCl_2_/2 mM EGTA/0.5% Nonidet P-40/20 mM Tris·HCl, pH 6.8 with the addition of complete protease inhibitor cocktail). The lysates were centrifuged at 14,000 rpm for 10 min at RT. Supernatants (containing soluble tubulin) and pellets (containing polymerized tubulin) were separated and each sample was brought to an equal volume with hypotonic buffer. Proteins were resolved by SDS/PAGE on 4–12% gels (Invitrogen, Carlsbad, CA), transferred to nitrocellulose and immunoblotted with an antibody against tubulin followed by fluorescent secondary antibody. Immunoreactive bands were detected using an Odyssey photometer (Li-Cor, Lincoln, NB).

### Tubulin in vitro polymerization assay

A commercial fluorescence-based tubulin polymerization assay (Cytoskeleton, Denver, CO; Cat. # BK011P) was used as per manufacturer’s instructions for detection of tubulin polymerization inhibitors. Briefly, porcine tubulin (3 mg/ml tubulin in 80 mM PIPES pH 7.0, 0.5 mM EGTA, 2 mM MgCl_2_, 1 mM GTP and 10% glycerol) was incubated at 37 °C with either vehicle control (DMSO), with Paclitaxel or antimitotic compound. Each condition was tested in duplicate. Polymerization was measured over time by excitation at 360 nm and emission at 420 nm in a temperature controlled 96-well plate fluorimeter (Envision 2105, Perkin-Elmer, Waltham, MS) equipped with filters for excitation at 340–360 nm and emission at 420–450 nm.

### Dose escalation and pharmacokinetics in mice

C57Bl/6 mice (aged 8–12 weeks) were injected subcutaneously with escalating doses of WEHI-7326 (0.8, 2.4, 7.2, 21.5, 64.5 mg/kg) in sterile water. Mice were monitored daily for general health and behavior, and were sacrificed and examined 4 days post-injection. Animal studies were conducted in line with the LICR/DoS Animal Ethics Committee guidelines (11/05 A).

The non-GLP pharmacokinetic studies were performed by the Centre for Drug Candidate Optimization, Monash University. All animal studies were conducted in accordance with the Australian Code of Practice for the Care and Use of Animals for Scientific Purposes, and the study protocols were reviewed and approved by the Monash Institute of Pharmaceutical Sciences Animal Ethics Committee.

Briefly, intravenous pharmacokinetics and systemic exposure of WEHI-7326 were assessed in non-fasted male Swiss Outbred mice. WEHI-7326 was administered via intravenous bolus injection (10 ml/kg dose volume) and blood samples were collected at 1, 2, 5, 15 and 30 min and 1, 2, 4, 7.5 and 24 h post-dose (*n* = 2 mice per time point). A maximum of two samples were obtained from each mouse, with samples being taken either via submandibular bleed (approximately 120 μl; conscious sampling) or terminal cardiac puncture (0.6 ml; while mice were anaesthetized using inhaled Isoflurane). Once collected, blood samples were centrifuged immediately, supernatant plasma was removed, and stored in −20 °C until analysis by LC-MS

Parameters were calculated with standard calculations:

$$Plasma\,CL = \frac{{\mathop {{Dose}}\nolimits_{IV} }}{{\mathop {{AUC}}\nolimits_{IV} }}\mathop {V}\nolimits_{{\mathrm{SS}}} = \frac{{\mathop {{AUMC}}\nolimits_{IV} }}{{\mathop {{AUC}}\nolimits_{IV} }} \ast Plasma\,CL$$

With plasma CL = plasma clearance, AUC_IV_ = area under the plasma concentration vs. time profile, V_SS_ = apparent volume of distribution at steady state, AUMC_IV_ = area under the first moment of the plasma concentration versus time profile from time zero to infinity after IV administration.

### Tumor xenografts in nude mice

Tumor cell lines LIM2537, U87MGΔ2–7EGFR or H1437 were collected by trypsinization, counted and injected at 5 × 10^6^ cells/ site subcutaneously on both flanks of BALB/c nude mice. Treatment with vehicle control or WEHI-7326 at given dose was initiated after 5 days. Drug or vehicle (100 μl) was injected intraperitoneally three times weekly. Control group and treatment group consisted of 8 mice each. Tumors were measured twice weekly with calipers. Tumor volumes were estimated using the modified ellipsoid formula V = 1/2 (length × width^2^). The experiments were terminated when tumors in the control group started ulcerating or exceeded 17 mm in length, in line with the LICR/DoS Animal Ethics Committee guidelines (11/05 A).

LoVo (4 × 10^6^), PC3 (3 × 10^6^) or MDA-MB-231 LNA DRE (10^6^) cells were collected by trypsination and injected with 50 μl 1:1 PBS:Matrigel (v/v) subcutaneously on the right flank of 18 female (LoVo), 40 male (PC3) or into 4^th^ mammary fat pad of 40 female (MDA231) Balb/c nude mice. Tumor growth was monitored and average tumor size at commencement of dosing was 100 mm^3^. Mice were randomized into 3 arms of each 5 (LoVo), 10 (PC3) or 12 (MDA23) mice respectively and treatment with vehicle (saline), WEHI-7326 (biweekly) or docetaxel (weekly) was administered via tail vein injection. Tumor volumes were measured at least twice weekly with calipers and the experiments were terminated when tumors in the control group started exceeding 1500 mm^3^. For LoVo, both vehicle and 25 mg/kg WEHI-7326 groups were harvested 24 h, but 20 mg/kg WEHI-7326 group harvested 1 h post final dose. For PC3, docetaxel group was not harvested in an attempt to grow out resistant tumors.

### Ethics approvals—human breast, lung and ovarian cancer PDX models

Written informed consent was obtained from all patients by the Royal Melbourne Hospital Tissue Bank and the Victorian Cancer Biobank prior to inclusion in the study, according to protocols relevant institutional review board approval. Human ovarian cancer tissue was obtained from informed and consenting patients enrolled in the Australian Ovarian Cancer Study. Human Ethics approval was obtained from the Human Research Ethics Committee at the Walter and Eliza Hall Institute (05/06, 10/05, 10/04) and the Royal Women’s Hospital (10–57). NOD-SCID-IL2Rγc–/– mice were bred and maintained according to institutional guidelines. Animal experiments were approved by the WEHI Animal Ethics Committee (2017.002, 2016.024, 2016.023, 2013.028) or the Peter MacCallum Cancer Centre Animal Ethics Committee (E476).

### Breast cancer PDX models and in vivo experiments

Cohorts of about 30 female mice were seeded with thawed single cell suspensions of early passage human breast tumors (passage 2 or 3). Briefly, 150,000–250,000 cells were resuspended in 10 µl of transplantation buffer (50% fetal calf serum, 10% of a 0.04% trypan blue solution and 40% PBS) and growth-factor-reduced Matrigel [BD] at a ratio of 3:1, and injected into the cleared mammary fat pads of 3- or 4-week-old NOD-SCID-IL2Rγc–/– female mice. Mice were monitored for tumor development three times weekly and tumor size measured using digital Vernier calipers. Tumor volume was estimated by measuring the minimum and maximum tumor diameters using the formula: (minimum diameter)^2^ x (maximum diameter)/2. Once tumors arose, mice were randomized into treatment arms. Treatment was initiated when the tumor volume reached 80–120 mm^3^. Randomization and tumor measurements were managed using the Study Director software (v 3.0, studylog). Mice were sacrificed at the first measurement where tumor volume exceeded 600 mm^3^, or if their health deteriorated for reasons other than disease progression or drug toxicity (censored event).

### Breast cancer PDX models—in vivo treatment

Mice were treated with either docetaxel (10 mg/kg; i.p.; once), cisplatin (4 mg/kg; i.p.; once) or WEHI-7326 (20 mg/kg; twice a week (Monday and Thursday); i.v.). Vehicle treated mice were administered with the vehicle for WEHI-7326. Docetaxel was resuspended at 2 mg/ml in PBS:Tween-80:ethanol = 90:5:5 (v/v/v). WEHI-7326 was resuspended at 2 mg/ml in saline.

### Breast cancer PDX models—in vivo proliferation and apoptosis

Human xenografted tumors were collected, fixed in neutral buffered formalin before embedding in paraffin. Sections were subjected to antigen retrieval and then incubated with antibodies against Ki-67 (BD Pharmingen) and cleaved caspase-3 (Cell Signaling) for 30 min at room temperature, followed by biotinylated anti-IgG secondary antibodies (Vector Labs). Signal detection was performed using ABC Elite (Vector Labs) for 20 min and 3,3′-diaminobenzidine (Dako) 5 min at room temperature. BrdU (Cell Signaling) staining was performed according to the manufacturer’s instructions. BrdU positive cells were counted using Fiji software while cleaved caspase-3 were counted manually on 2-3 fields from three independent tumors.

### Lung cancer PDX models and in vivo experiments

LUSC PDXs were generated as described in^25^. PDXs at passage 4 were defrosted and washed with PBS before counting. 200,000 to 500,000 cells were then injected in a 50:50 PBS:Matrigel mix into the flanks of NOD-SCID-IL2Rγc–/– male mice. Tumors were measured twice weekly with calipers and treatment started when tumors were between 70–120 mm^3^. Mice were assigned to treatment groups based on average tumor volume per group. Mice were culled when tumors reached a volume of 600 mm^3^.

### Ovarian cancer PDX models and in vivo experiments

Two to three mm ovarian tumor fragments were subcutaneously engrafted in the left flank of NSG mice and monitored by physical palpation for tumor growth and engraftment. Engrafted tumors were measured twice a week using digital Vernier calipers and maximum tumor diameter + perpendicular tumor length were digitally recorded into the Study Director software (v 3.0/4.0 StudyLog). Tumor volume was calculated automatically (tumor volume (mm^3^) = 0.5236 x [larger diameter (mm) x smaller diameter (mm)^2^]). Mice were randomized to treatment once their tumor volume reach 180–300 mm^3^ and tumor measurements were performed twice a week up to 120 days or when the tumor volume reaches 700 mm^3^.

Time to progressive disease (PD): Defined as the time (in days) from beginning of treatment to an increase in average tumor volume of >20% from the nadir (taken as the smallest average tumor volume recorded since treatment started or 200 mm^3^ if nadir was <200 mm^3^ as lesions smaller than this are difficult to measure with accuracy). Median time to harvest (TTH): Defined as the time (in days) from the beginning of treatment to day of harvest at 700 mm^3^ and the median TTH was calculated and plotted using Kaplan-Meier curves. Based on ref. ^27^.

Each PDX’s platinum sensitivities had been established by randomizing tumors to cisplatin treatment at 4 mg/kg IP injection given on day 1, day 8 and day 18 (MTD) or vehicle. A cisplatin-sensitive PDX demonstrates prolonged tumor response to treatment with no evidence of progressive disease (PD) before 100 days. The 100-day period has been chosen to differentiate between a platinum-resistant and a platinum-sensitive HGSOC PDX tumor. Platinum resistance was defined as a PDX which undergoes initial tumor response/regression to cisplatin treatment followed by PD observed <100 days from treatment. Lastly, a platinum-refractory PDX is defined as a tumor which continues to grow during treatment and has a time to PD of less than 50 days. These PDX cisplatin responses have been correlated with the patients’ responses to first-line platinum-based chemotherapy in the clinic^27^.

### High-risk neuroblastoma models and in vivo experiments

All animal studies were performed in accordance with the guidelines approved by University of New South Wales Animal Care and Ethics Committee (ACEC 17/53B) and in accordance with the requirements of the Australian Code for the Care and Use of Animals for Scientific Purposes. PDX models COG-N-440x and COG-N-496x were obtained from the Childhood Cancer Repository, Texas Tech University Health Sciences Center in Lubbock, TX, USA. 6–8 week old female NSG mice were engrafted by subcutaneous injection with 1 × 10^6^ freshly thawed PDX tumor cells into one flank in a mixture of 100 μl RPMI-1640 and 100 μl Matrigel. Mice were monitored for tumor development three times weekly and tumor size measured using digital Vernier calipers. Treatments commenced when tumors reached 50 mm^3^, following randomization into treatment arms. Once tumors reach 1000 mm^3^ or if the mice showed signs of poor health or weight loss, mice were humanely killed by CO_2_ asphyxiation. Mice were treated with either vincristine (0.5 mg/kg, once weekly i.p. in sterile saline for up to 5 weeks), WEHI-7326 (20 mg/kg; twice weekly i.v. in sterile saline for up to 5 weeks) or vehicle (as for WEHI-7326). For the Th-MYCN transgenic mouse model, spontaneous tumor growth in mice homozygous for the MYCN transgene was assessed by abdominal palpation at least twice a week. Treatments commenced once a small palpable tumor (~5 mm) was detected, following randomization to treatment arms. Mice were euthanized upon detection of an abdominal tumor of ~1 cm in diameter. Mice were also euthanized if showing signs of poor health, weight loss equal to or greater than 20% or if they displayed signs of a thoracic tumor not detectable by abdominal palpation (hind limb paralysis, bulging eyes or breathing difficulties, depending on where the tumor is located). Mice were treated with WEHI-7326 (20 mg/kg; twice weekly i.v. in sterile saline for up to 5 weeks), vehicle (as for WEHI-7326), cyclophosphamide (10 mg/kg, daily i.p. in sterile saline for 5 days) in combination with topotecan (0.5 mg/kg, daily i.p. in sterile saline for 5 days), irinotecan (2 mg/kg, daily i.p. in 5% glucose for 5 days) in combination with temozolomide (5 mg/kg, daily i.p. in 5% glucose for 5 days), WEHI-7326 in combination with cyclophosphamide/topotecan, or WEHI-7326 in combination with irinotecan/temozolomide (schedules as above).

### Statistical analysis

All in vitro experiments were repeated in at least three biological replicates. The details of experiments, including statistical tests used, number of experiments, and dispersion and precision measures, are stated in the figure legends. Sample size estimate for in vivo studies was based on past experience. Randomization and tumor measurements were managed using the Study Director software (v 3.0, studylog). All statistical analysis was done in GraphPad Prism, Version 8.3.1. Kaplan-Meier (log-rank test) was used to test for significant differences in the survival of mice (using the ethical end point for tumor size as a surrogate for death). Unpaired t tests were used to test the significance of differences in column means between treatments.

## Supplementary information

Figure S1

Figure S2

Figure S3

Figure S4

Figure S5

Figure S6

Figure S7

Figure S8

Figure S9

Figure S10

Figure S11

Figure S12

Figure S13

Figure S14

Figure S15

Figure S16

Figure S17

Figure S18

Table S1

Table S2

Table S3

Table S4

Table S5

Table S6

Table S7

Supplementary Figures Legends

Supplementary Information - In vivo toxicity

Supplementary Experimental Information

Supp Video S1: SW480 cells vehicle-treated

Supp Video S2: SW480 cells WEHI-7326-treated
