# Supplementary figures and images for: Preclinical small molecule WEHI-7326 overcomes drug resistance and elicits response in patient-derived xenograft models of human treatment-refractory tumors
Source: Cell Death Dis. 2021 Mar 12;12(3):268. doi: 10.1038/s41419-020-03269-0 (PMC7955127; doi:10.1038/s41419-020-03269-0)

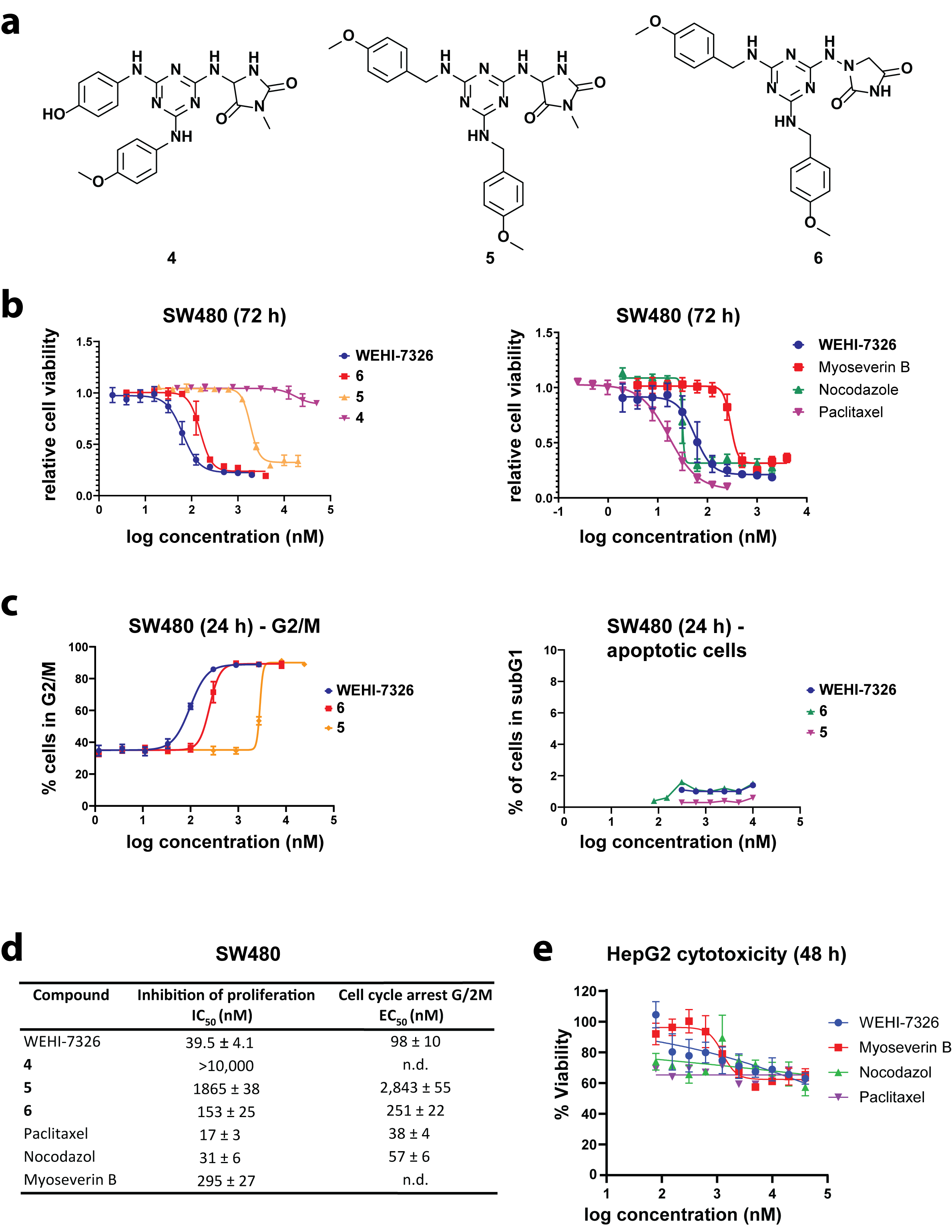

Supplement: Supplementary file 1 — Figure S1 [file 41419_2020_3269_MOESM1_ESM.png]

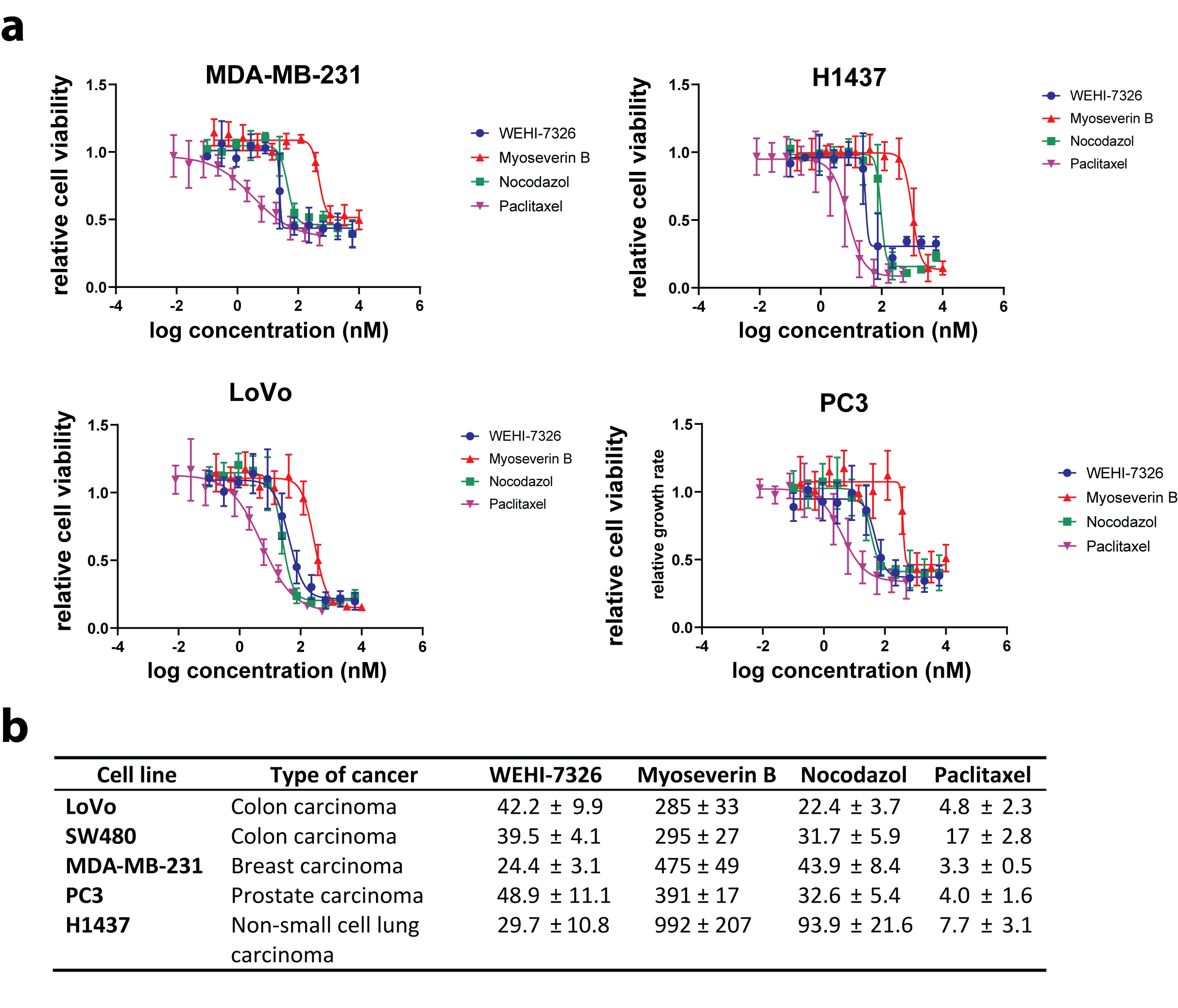

Supplement: Supplementary file 2 — Figure S2 [file 41419_2020_3269_MOESM2_ESM.png]

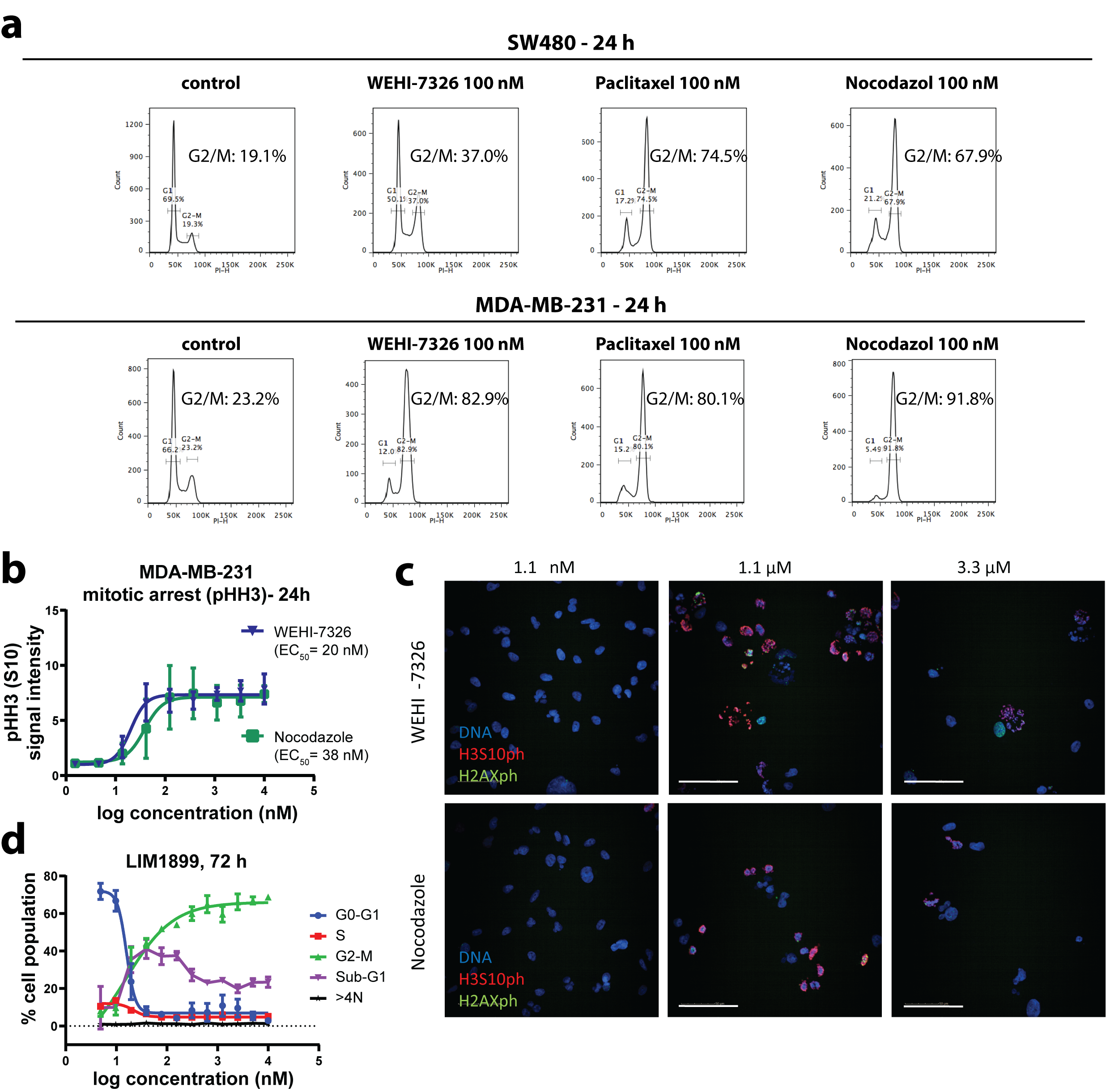

Supplement: Supplementary file 3 — Figure S3 [file 41419_2020_3269_MOESM3_ESM.png]

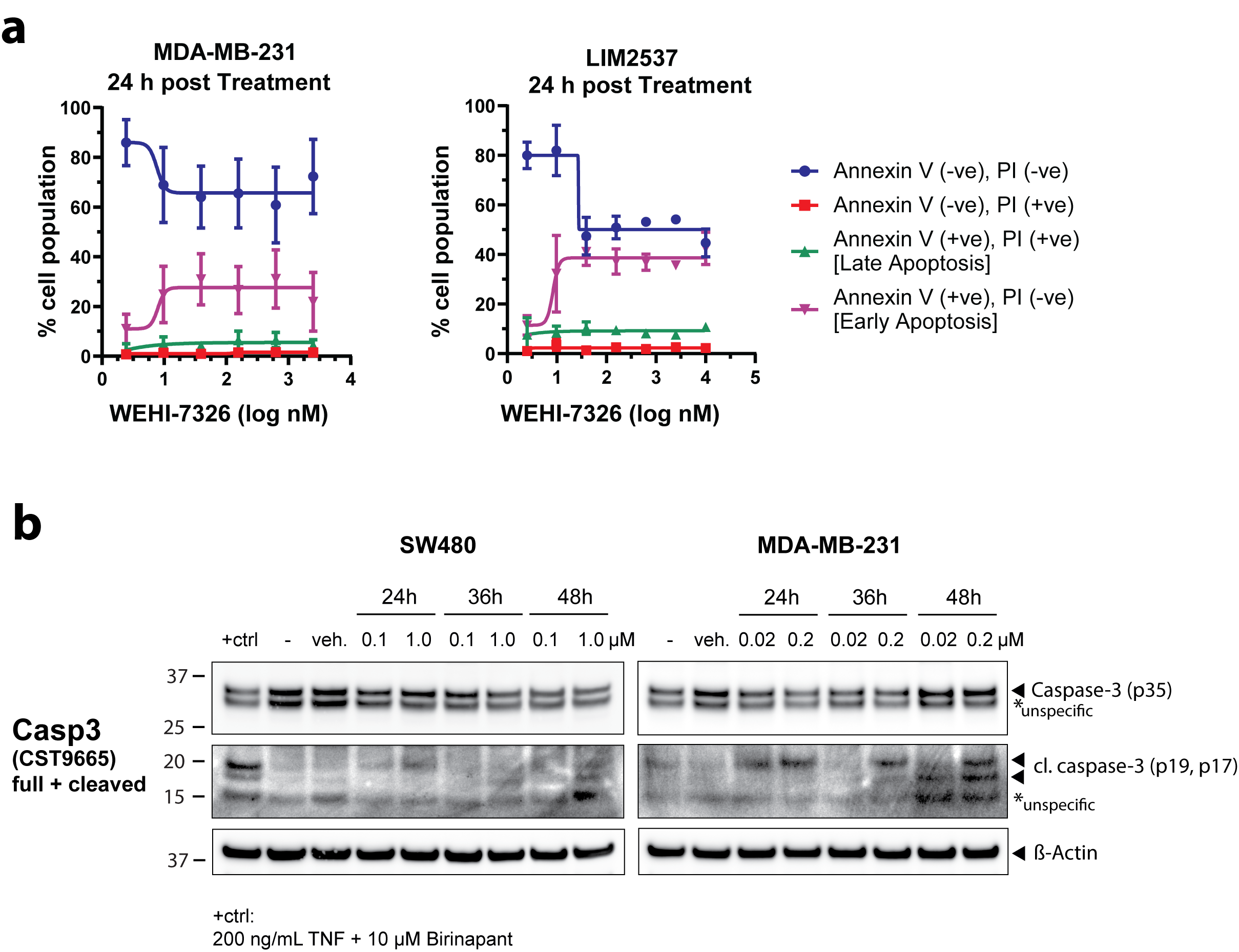

Supplement: Supplementary file 4 — Figure S4 [file 41419_2020_3269_MOESM4_ESM.png]

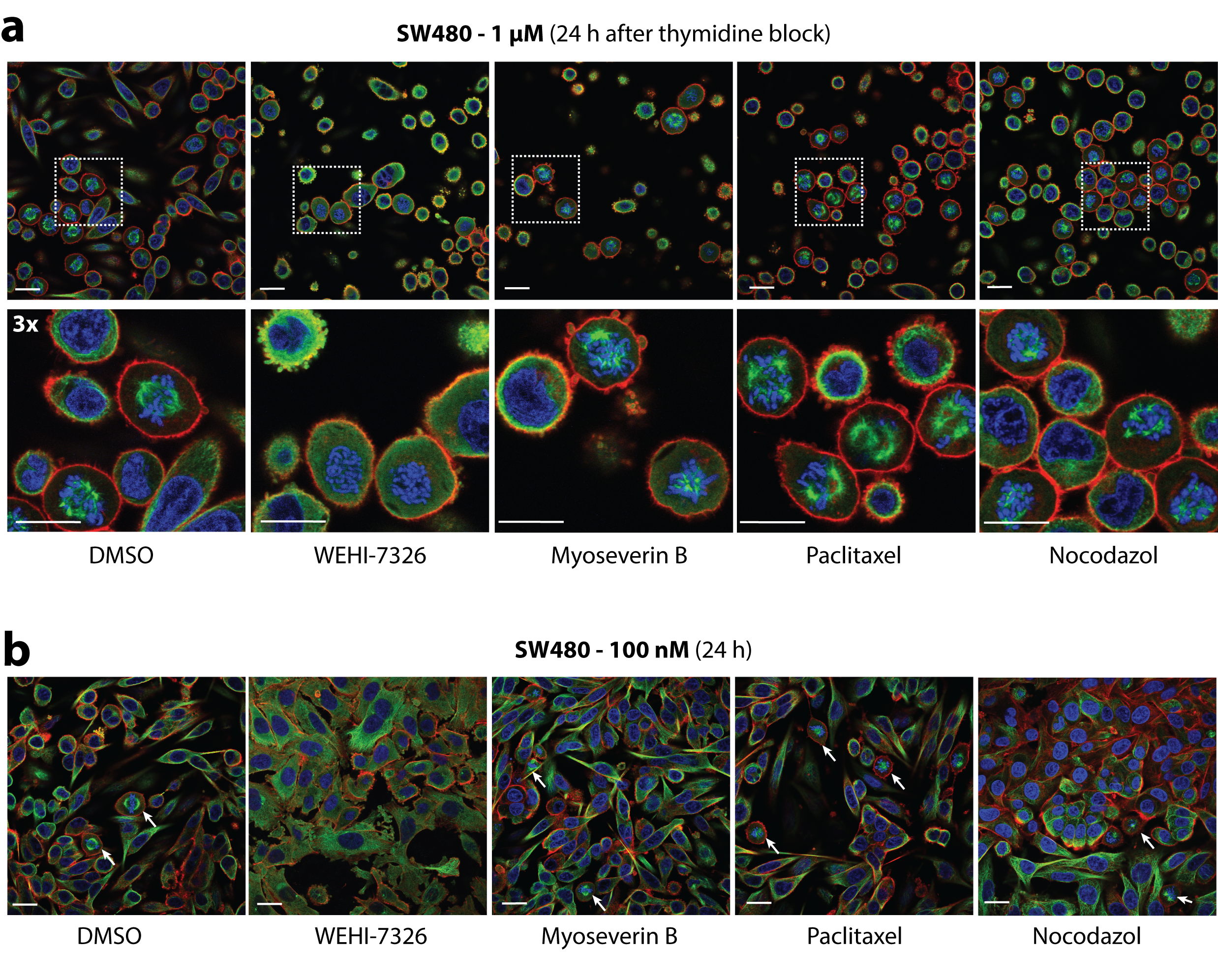

Supplement: Supplementary file 5 — Figure S5 [file 41419_2020_3269_MOESM5_ESM.png]

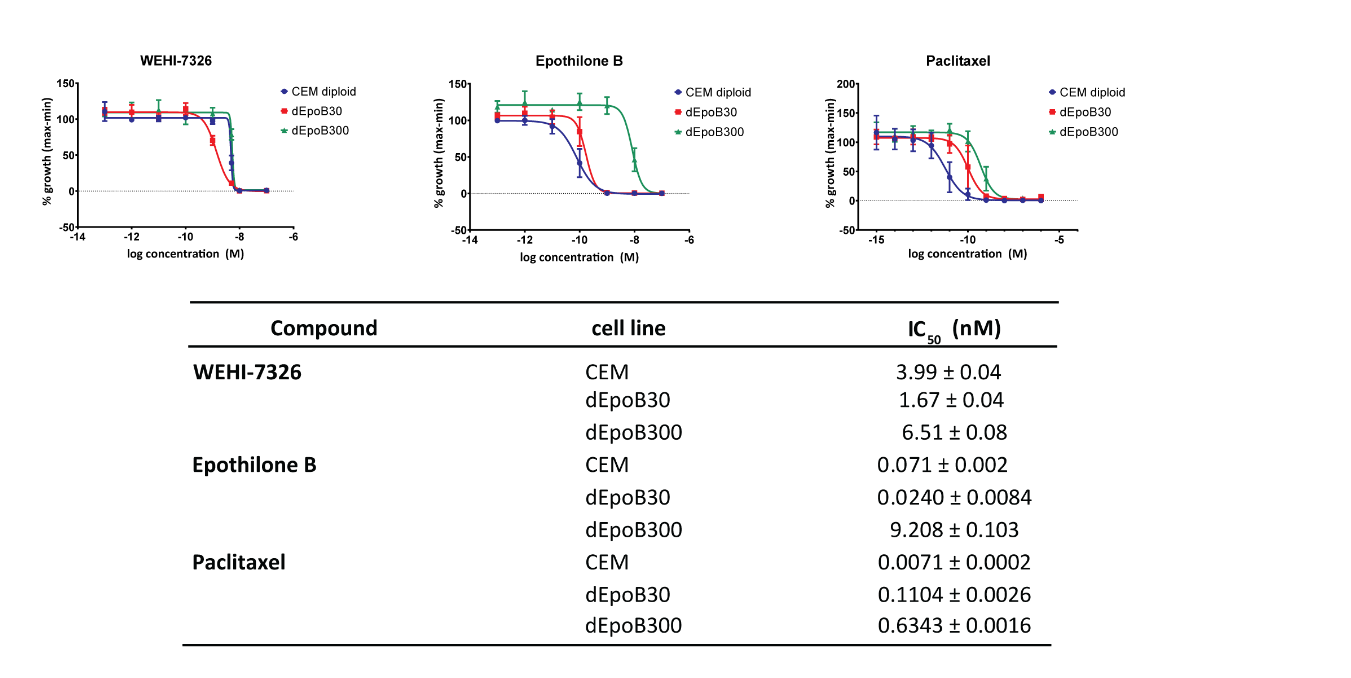

Supplement: Supplementary file 6 — Figure S6 [file 41419_2020_3269_MOESM6_ESM.png]

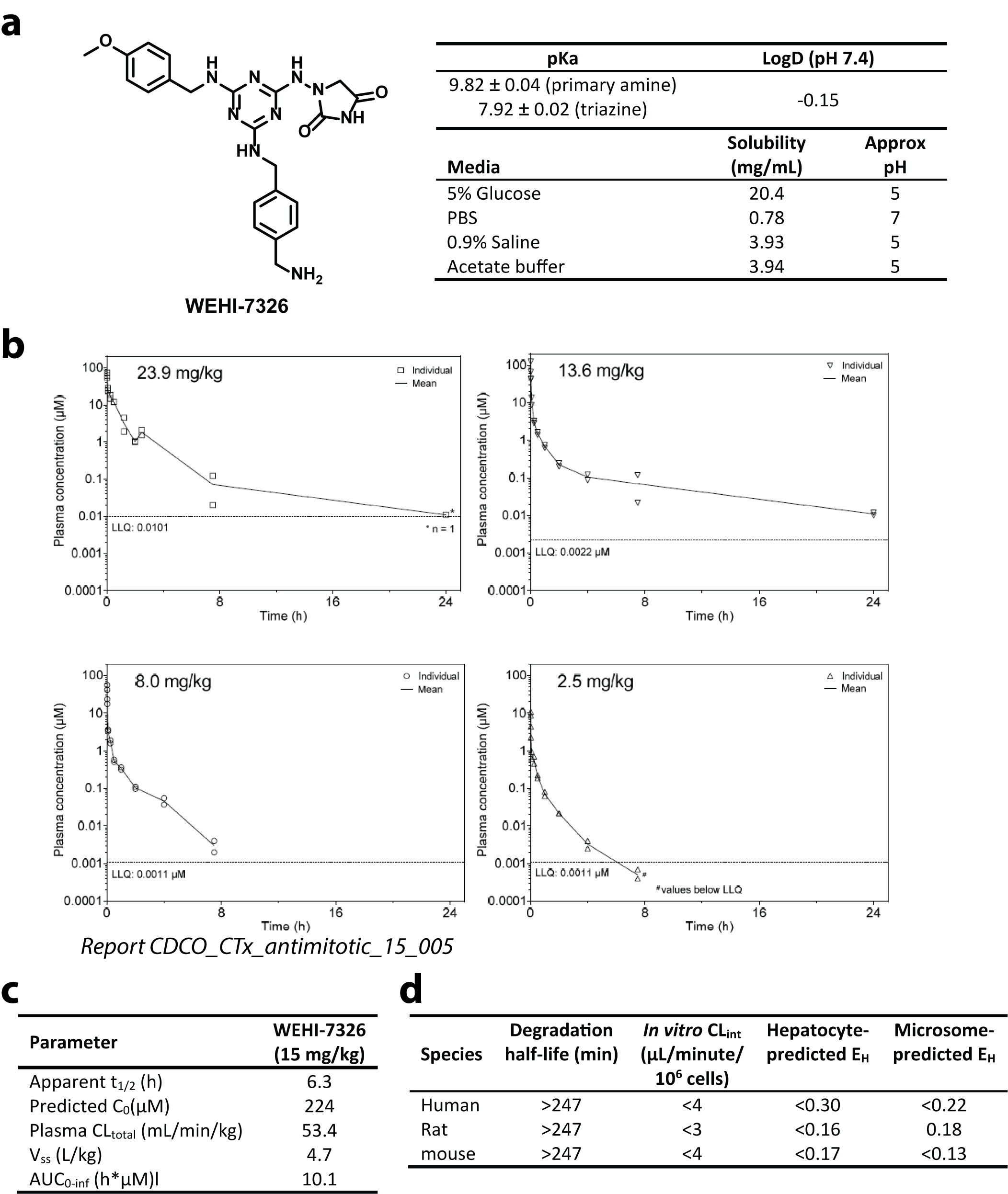

Supplement: Supplementary file 7 — Figure S7 [file 41419_2020_3269_MOESM7_ESM.png]

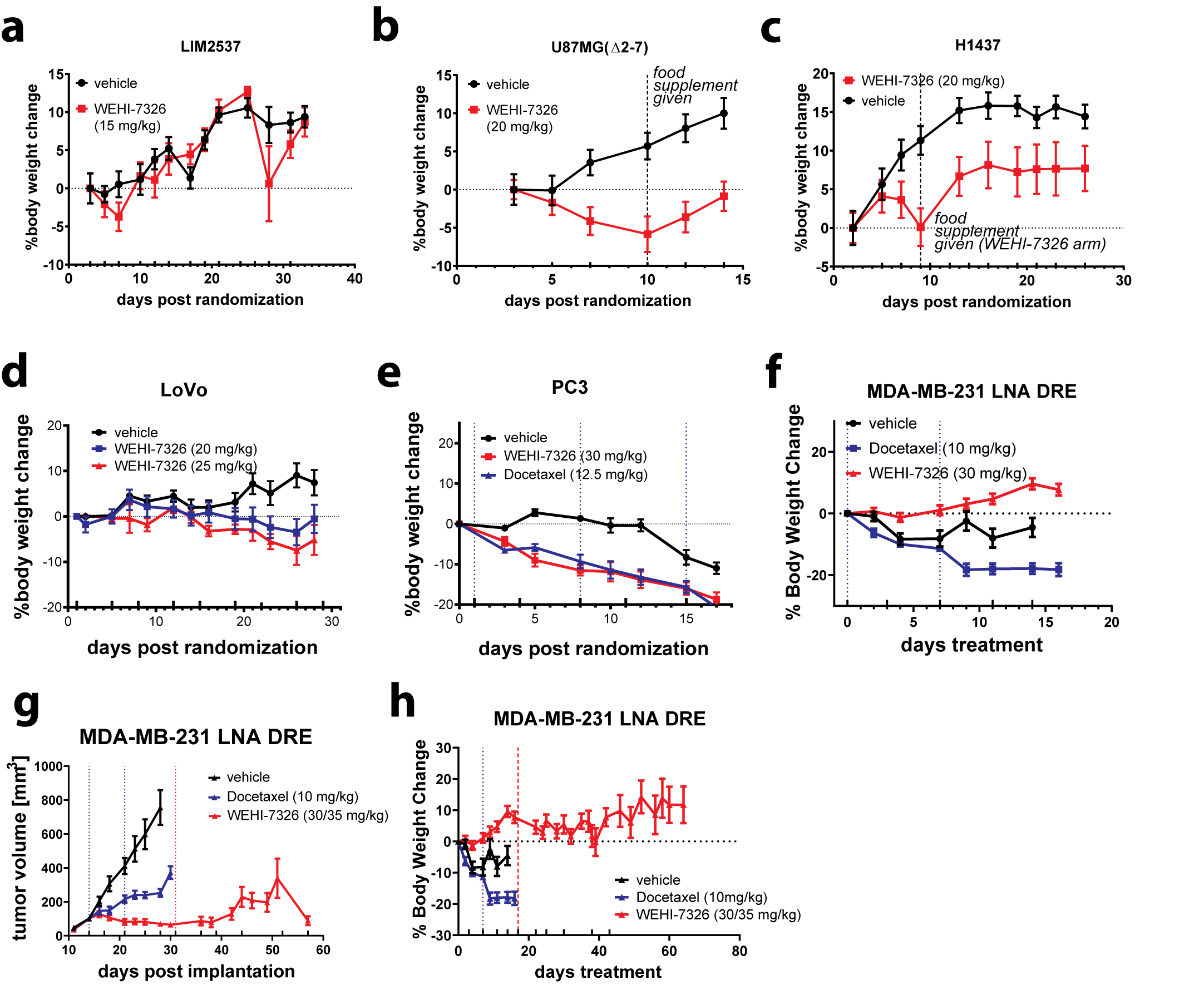

Supplement: Supplementary file 8 — Figure S8 [file 41419_2020_3269_MOESM8_ESM.png]

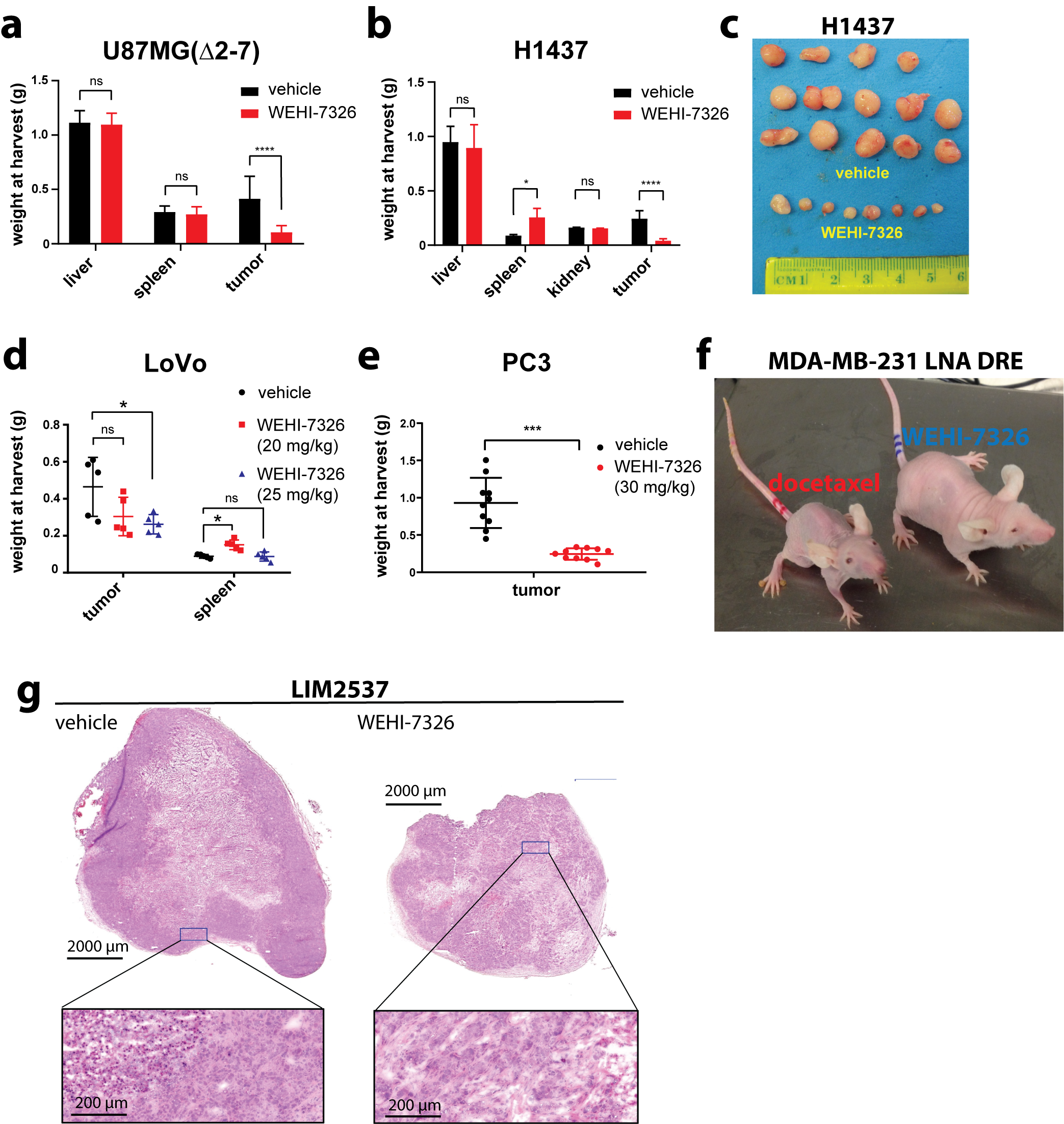

Supplement: Supplementary file 9 — Figure S9 [file 41419_2020_3269_MOESM9_ESM.png]

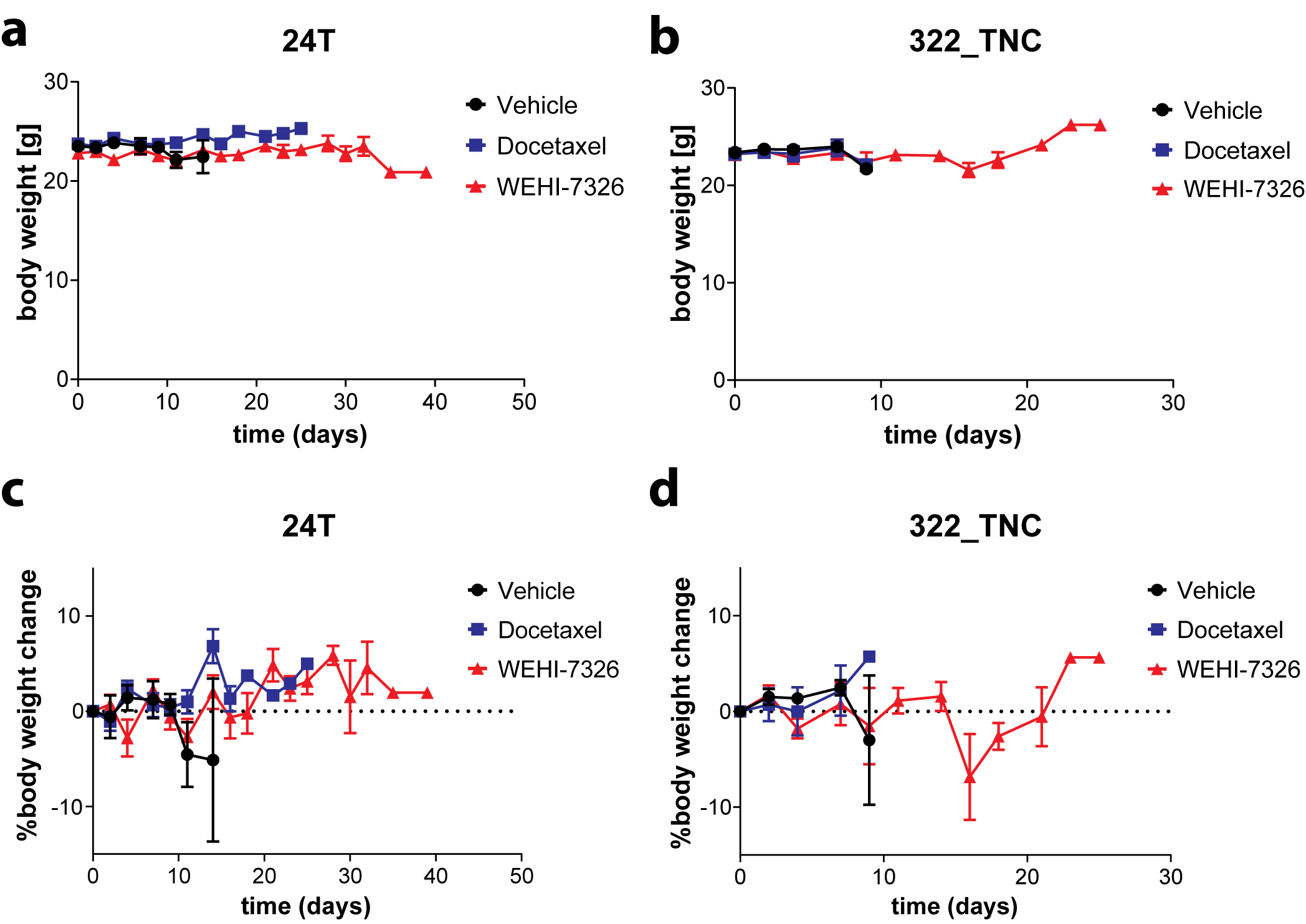

Supplement: Supplementary file 10 — Figure S10 [file 41419_2020_3269_MOESM10_ESM.png]

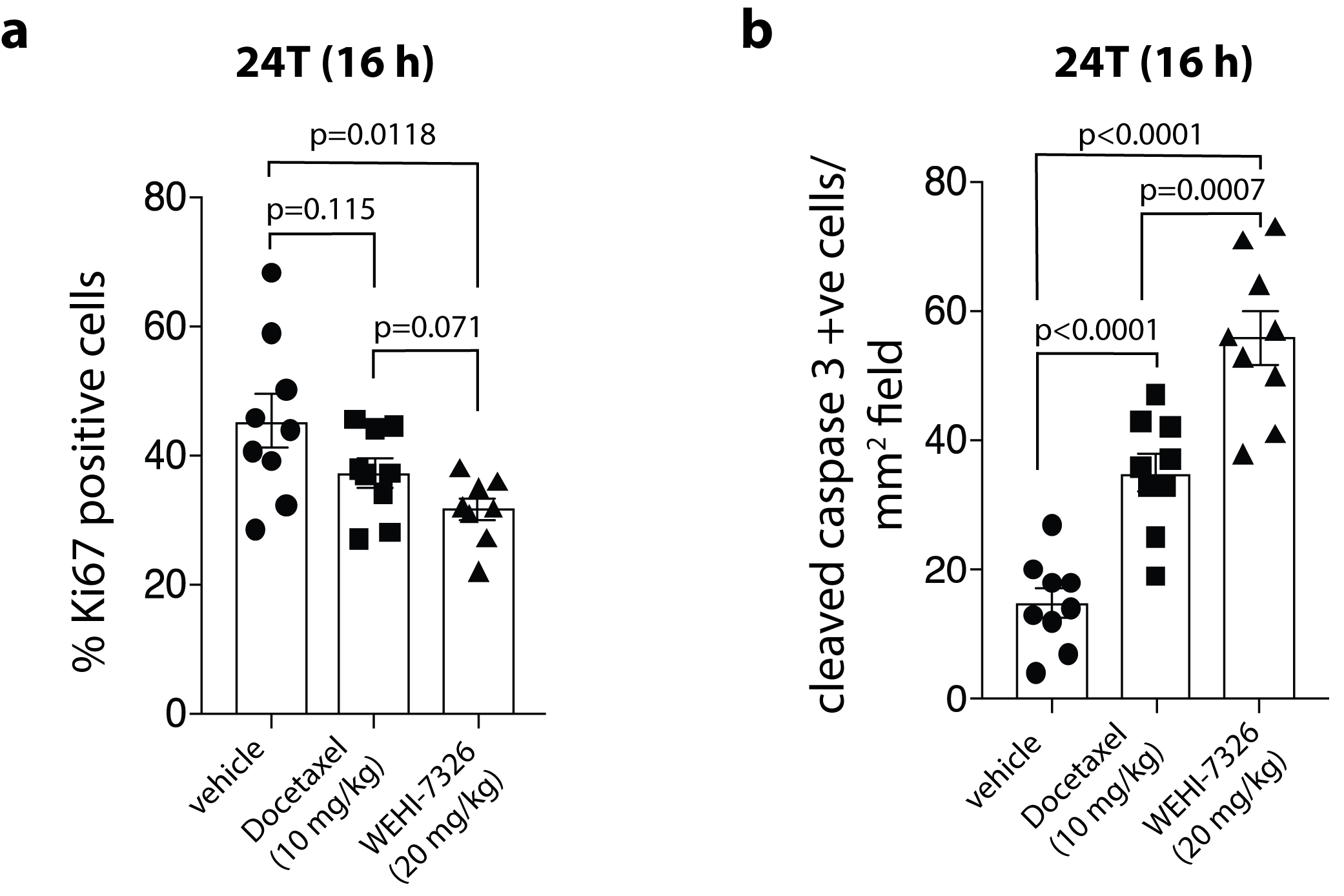

Supplement: Supplementary file 11 — Figure S11 [file 41419_2020_3269_MOESM11_ESM.png]

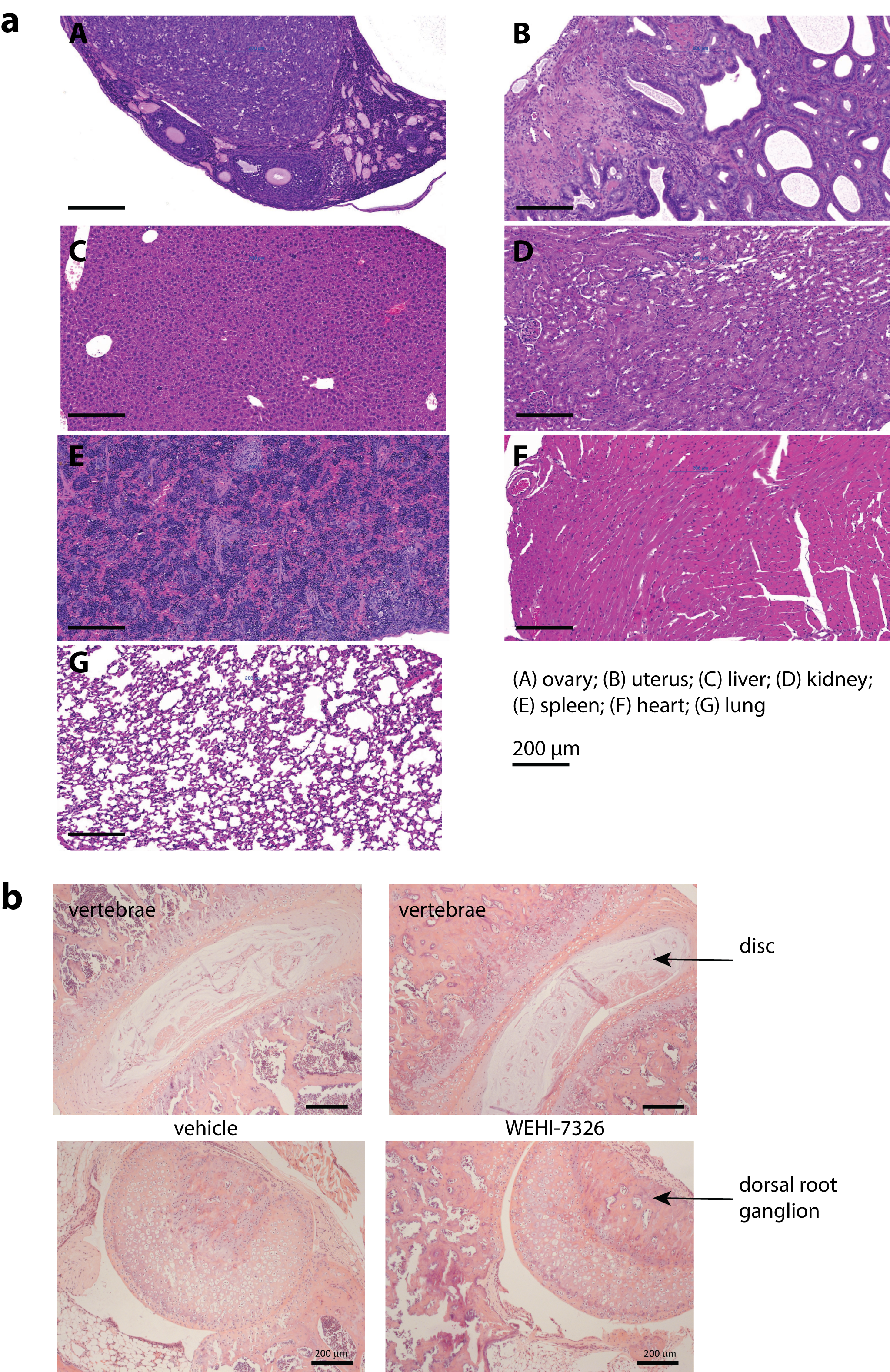

Supplement: Supplementary file 12 — Figure S12 [file 41419_2020_3269_MOESM12_ESM.png]

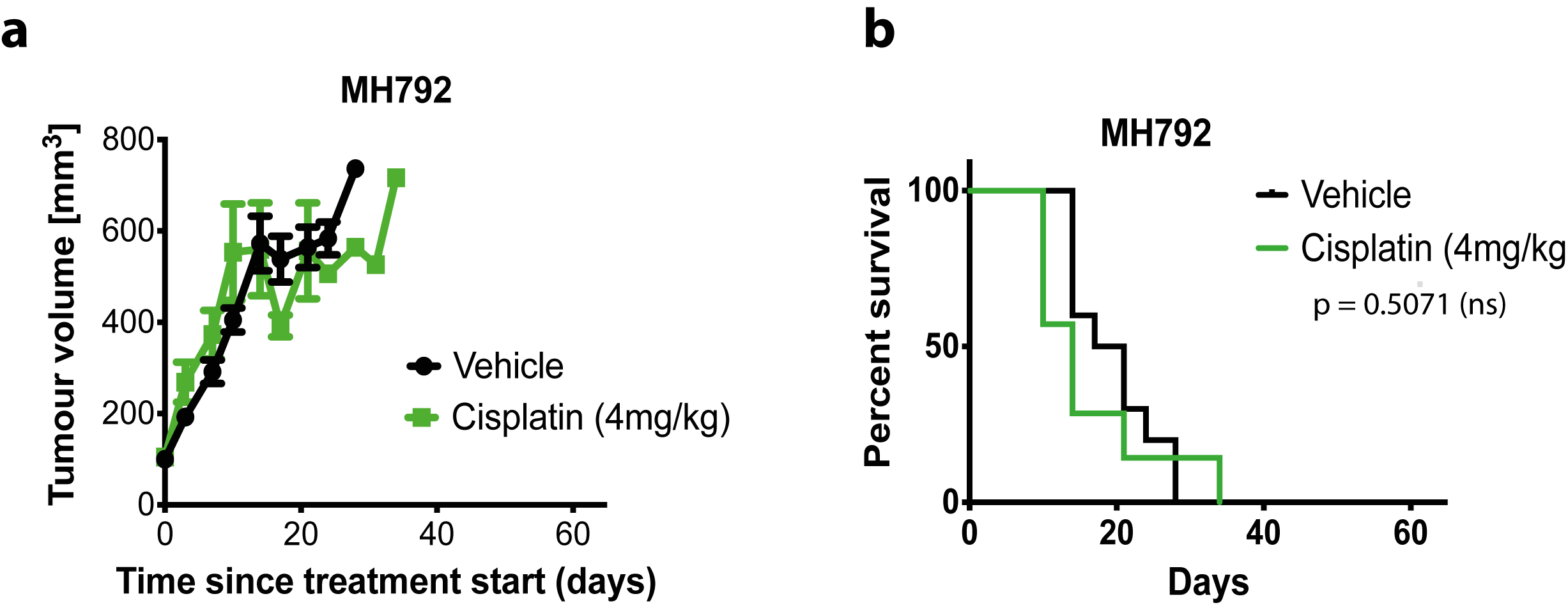

Supplement: Supplementary file 13 — Figure S13 [file 41419_2020_3269_MOESM13_ESM.png]

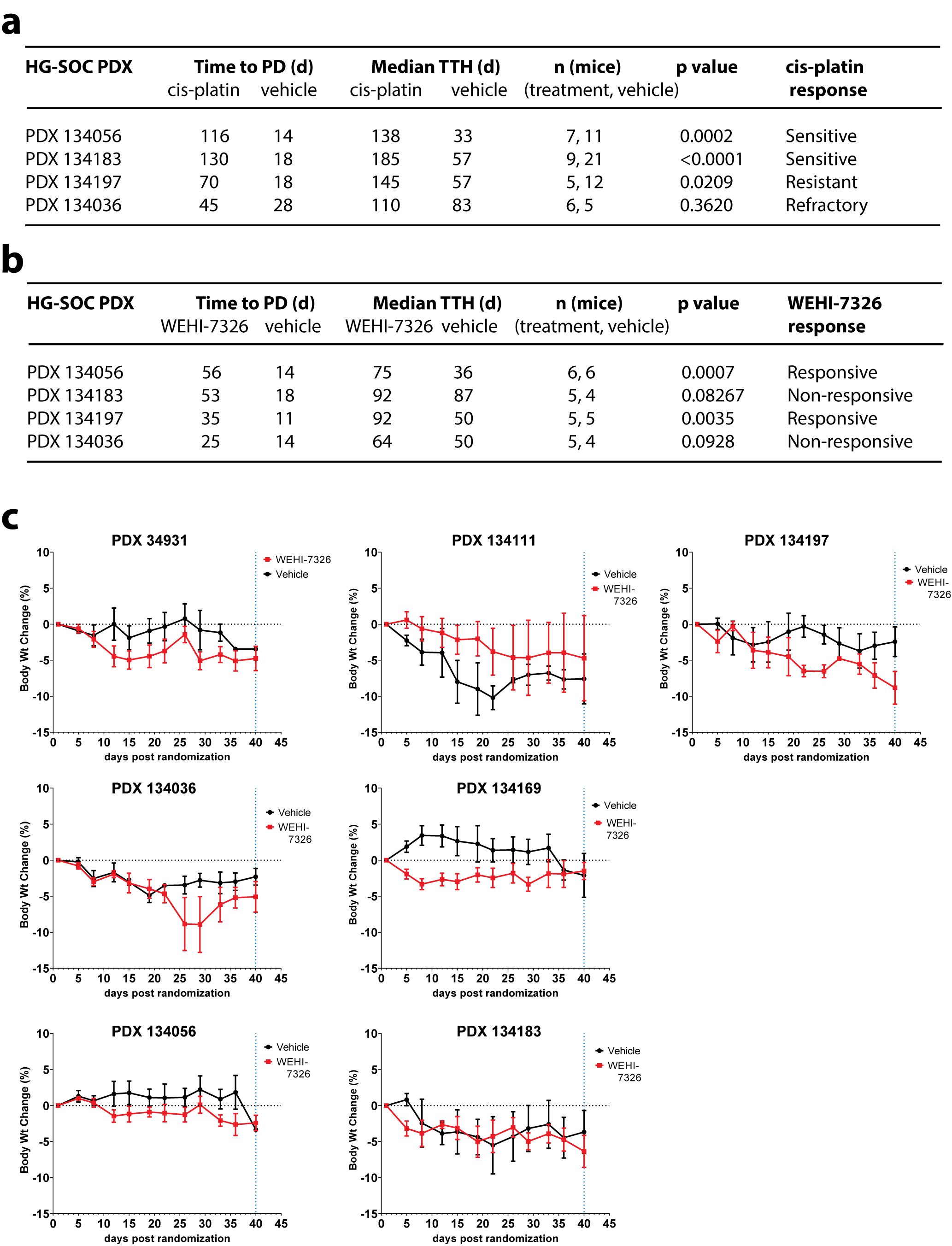

Supplement: Supplementary file 14 — Figure S14 [file 41419_2020_3269_MOESM14_ESM.png]

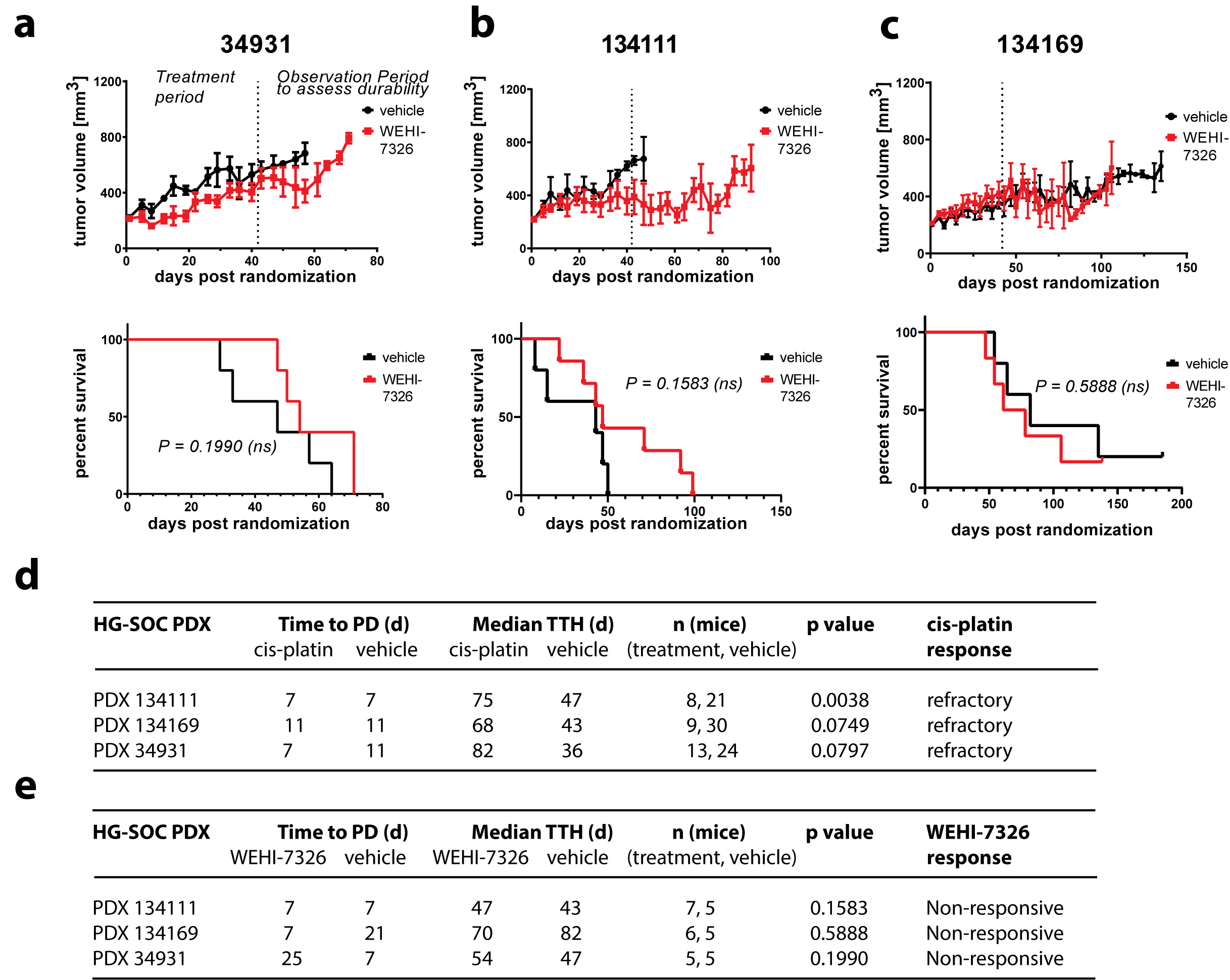

Supplement: Supplementary file 15 — Figure S15 [file 41419_2020_3269_MOESM15_ESM.png]

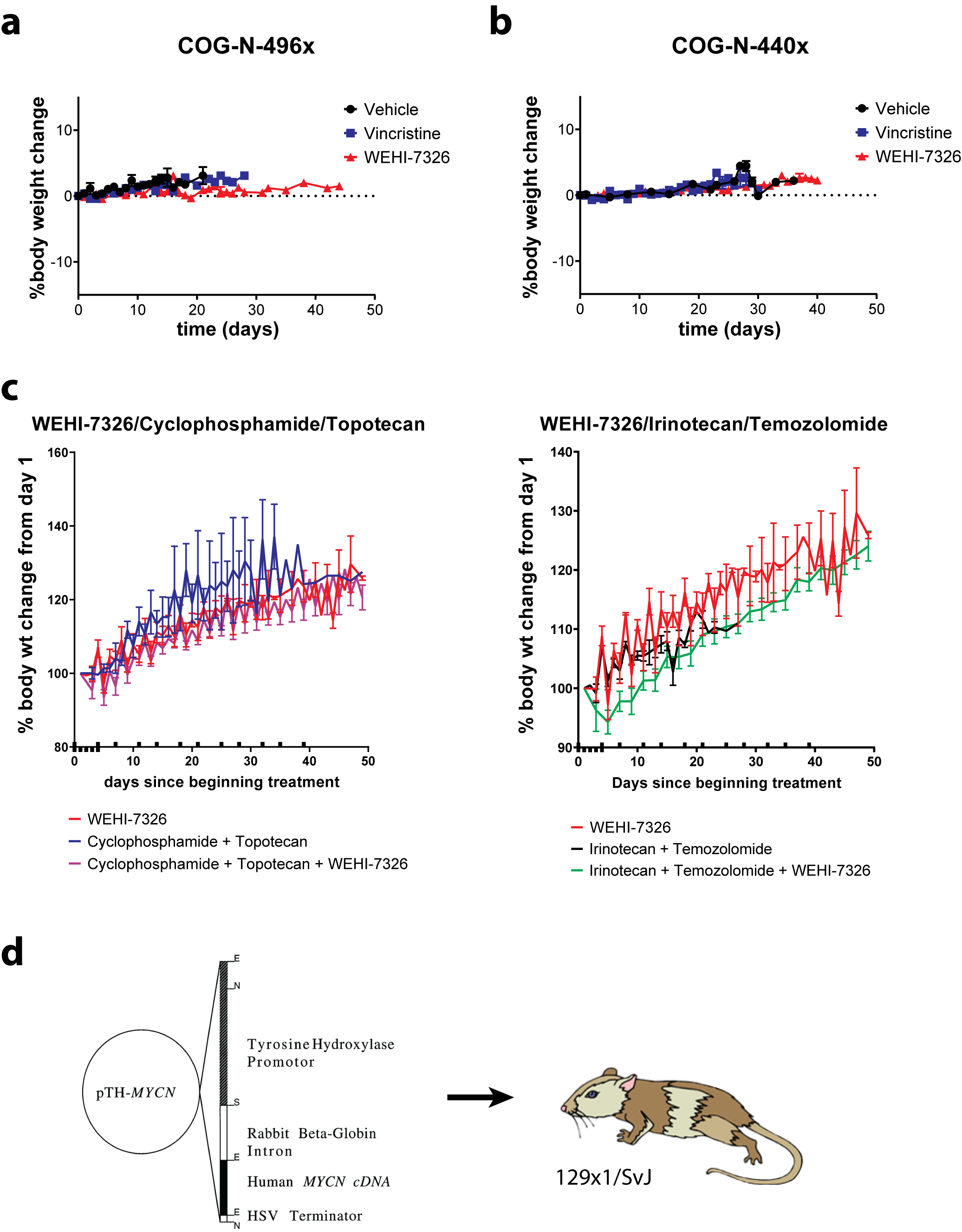

Supplement: Supplementary file 16 — Figure S16 [file 41419_2020_3269_MOESM16_ESM.png]

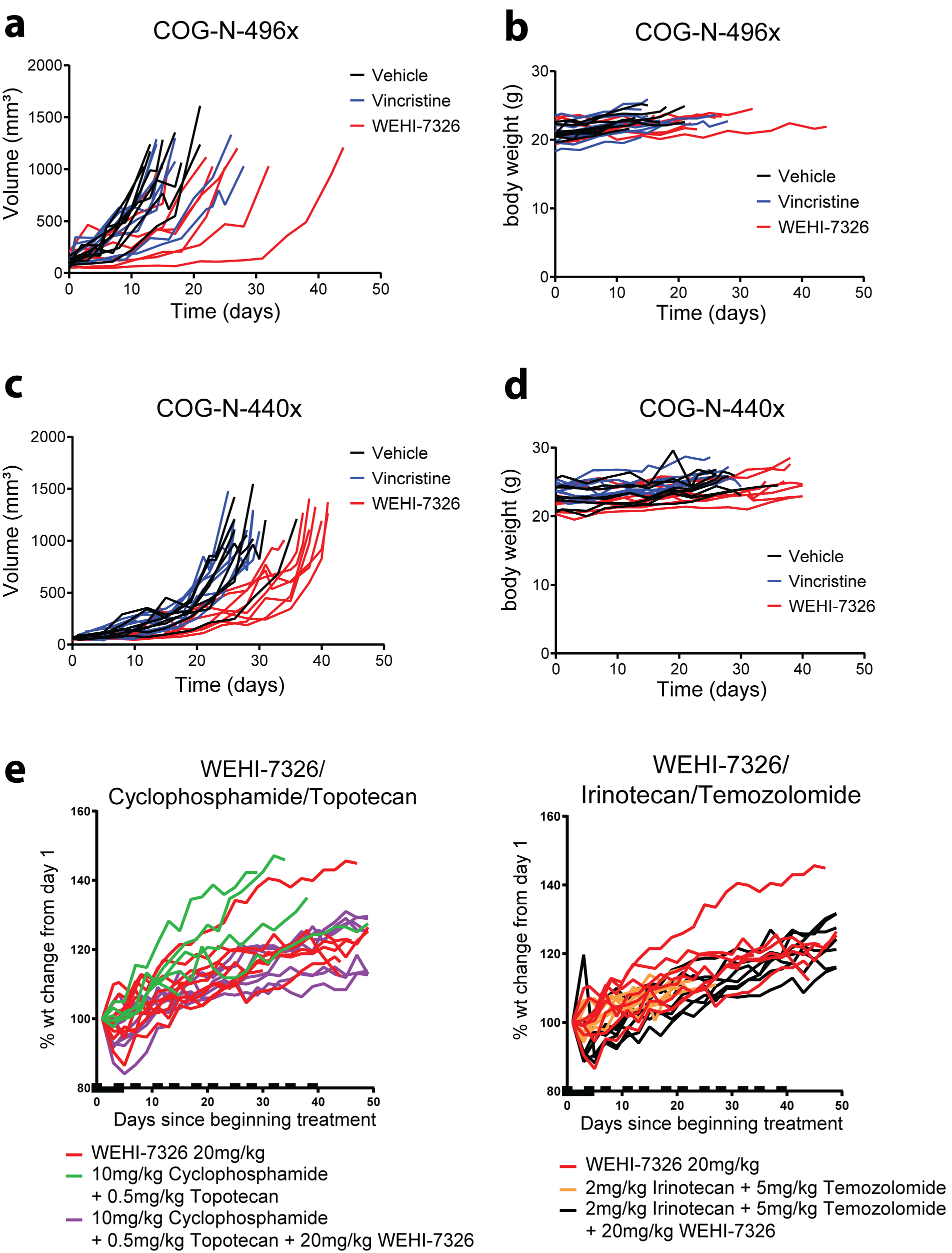

Supplement: Supplementary file 17 — Figure S17 [file 41419_2020_3269_MOESM17_ESM.png]

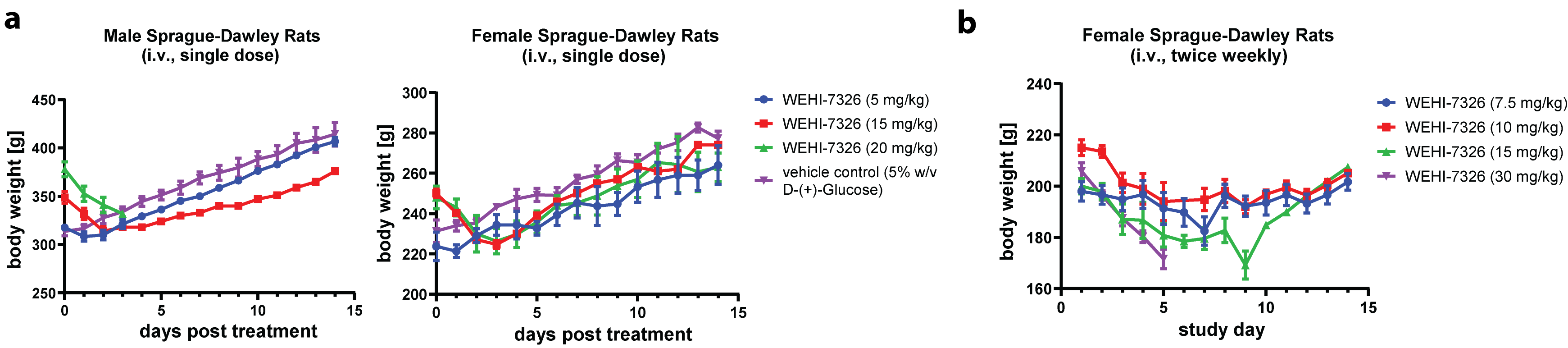

Supplement: Supplementary file 18 — Figure S18 [file 41419_2020_3269_MOESM18_ESM.png]
