# Supplementary material for: Preclinical small molecule WEHI-7326 overcomes drug resistance and elicits response in patient-derived xenograft models of human treatment-refractory tumors
Source: Cell Death Dis. 2021 Mar 12;12(3):268. doi: 10.1038/s41419-020-03269-0 (PMC7955127; doi:10.1038/s41419-020-03269-0)
Supplement: Supplementary file 19 — Table S1 [file 41419_2020_3269_MOESM19_ESM.docx]

**Table S1: Summary of Clinical Observations and Adverse Events – WEHI-7326 acute toxicity study following single intravenous dose administration in male and female Sprague-Dawley rats**

| **Group** | **Findings** | **Group 1**  **Vehicle control** | **Group 2**  **WEHI-7326**  **(5 mg/kg)** | **Group 3***  **WEHI-7326**  **(15 mg/kg)** | **Group 4**  **WEHI-7326**  **(20 mg/kg)** |
| --- | --- | --- | --- | --- | --- |
| **Males** | No remarkable observations. | 3/3 | 3/3 | 1/4 | 0/3 |
|  | Discharge from nose on study day 2 | 0/3 | 0/3 | 0/4 | 3/3 |
|  | Red stained right eye on study day 2 and 3 | 0/3 | 0/3 | 1/4 | 0/3 |
|  | Hunched posture with half shut eyes on Study Day 3. | 0/3 | 0/3 | 0/4 | 3/3 |
|  | Mild impairment of gait and lack of response to stimuli postdose, resolved 2 hours post treatment. | 0/3 | 0/3 | 0/4 | 3/3 |
|  | Labored breathing, severely affected gait and lack of response to stimuli post-dose (termination required) | 0/3 | 0/3 | 1/4 | 0/3 |
|  | Body weight loss >10% (termination required). | 0/3 | 0/3 | 2/4 | 3/3 |
| **Female** | No remarkable observations. | 3/3 | 3/3 | 1/3 | 2/3 |
|  | Mild impairment of gait and lack of response to stimuli up to 10 minutes post-dose. | 0/3 | 0/3 | 0/3 | 1/3 |
|  | Mild fur loss around both eyes from Study Day 10 to 15. | 0/3 | 0/3 | 0/3 | 1/3 |
|  | Body weight loss >10% (termination required). | 0/3 | 0/3 | 2/3 | 0/3 |
