# Supplementary material for: Preclinical small molecule WEHI-7326 overcomes drug resistance and elicits response in patient-derived xenograft models of human treatment-refractory tumors
Source: Cell Death Dis. 2021 Mar 12;12(3):268. doi: 10.1038/s41419-020-03269-0 (PMC7955127; doi:10.1038/s41419-020-03269-0)
Supplement: Supplementary file 20 — Table S2 [file 41419_2020_3269_MOESM20_ESM.docx]

**Table S2: Macroscopic Pathology Summary– Toxicity study**

| **Group** | **Findings** | **Group 1**  **Vehicle control** | **Group 2**  **WEHI-7326**  **(5 mg/kg)** | **Group 3***  **WEHI-7326**  **(15 mg/kg)** | **Group 4**  **WEHI-7326**  **(20 mg/kg)** |
| --- | --- | --- | --- | --- | --- |
| **Males** | Multifocal red petechiae on all lung lobes | 1/3 | 0/3 | 0/1 | 0/0 |
|  | White streaks though one or both testes | 0/3 | 3/3 | 1/1 | 0/0 |
|  | Smaller right teste | 0/3 | 1/3 | 0/1 | 0/0 |
|  | Testes noted to have a soft consistency | 0/3 | 0/3 | 1/1 | 0/0 |
| **Female** | Multifocal red petechiae on all lung lobes | 0/3 | 1/3 | 0/1 | 0/3 |
|  | Pale yellow liver (minimal) | 0/3 | 1/3 | 0/1 | 0/3 |
|  | Lung - haemorrhagic area right caudal lobe (1mm diameter) | 0/3 | 0/3 | 0/1 | 1/3 |
|  | Lung - focal red areas medial right lobe (2 mm diameter) | 0/3 | 0/3 | 0/1 | 1/3 |
