# Supplementary material for: Preclinical small molecule WEHI-7326 overcomes drug resistance and elicits response in patient-derived xenograft models of human treatment-refractory tumors
Source: Cell Death Dis. 2021 Mar 12;12(3):268. doi: 10.1038/s41419-020-03269-0 (PMC7955127; doi:10.1038/s41419-020-03269-0)
Supplement: Supplementary file 21 — Table S3 [file 41419_2020_3269_MOESM21_ESM.docx]

**Table S3: Haematology and Coagulation – WEHI-7326 acute toxicity study (day 15).**

|  | | |  | | **Males** | |  | |
| --- | --- | --- | --- | --- | --- | --- | --- | --- |
| **Analyte** | **Unit** | **Group 1 (D-(+)-Glucose, 10 mL/kg)** | | **Group 2 (WEHI-7326,**  **5 mg/kg)** | | **Group 3 (WEHI-7326,**  **15 mg/kg)** | |  |
| WBC | 10^9^/L | | 12.95 ± 2.94 | | 14.83 ± 1.08 | | 10.89 ± 0.00 | |
| RBC | 10^12^/L | | 8.14 ± 0.54 | | 7.71 ± 0.45 | | 7.68 ± 0.00 | |
| HGB | g/L | | 156.7 ± 12.4 | | 151.7 ± 3.1 | | 138.0 ± 0.0 | |
| HCT | L/L | | 0.53 ± 0.04 | | 0.52 ± 0.01 | | 0.46 ± 0.00 | |
| MCV | fL | | 65.5 ± 0.7 | | 67.2 ± 3.5 | | 60.40 ± 0.0 | |
| MCH | pg | | 19.23 ± 0.40 | | 19.70 ± 0.85 | | 18.00 ± 0.00 | |
| MCHC | g/L | | 293.7 ± 3.1 | | 294.0 ± 5.0 | | 297.0 ± 0.0 | |
| PLT | 10^9^/L | | 826.0 ± 47.0 | | 1025.0 ± 167.5 | | 1075 ± 0.0 | |
| Retc. | % | | 3.86 ± 0.61 | | 4.57 ± 1.00 | | 2.70 ± 0.00 | |
| Neut. | 10^9^/L | | 3.08 ± 1.29 | | 2.91 ± 0.44 | | 1.89 ± 0.00 | |
| Lymph. | 10^9^/L | | 9.21 ± 2.06 | | 11.13 ± 0.76 | | 8.47 ± 0.00 | |
| Mono. | 10^9^/L | | 0.30 ± 0.07 | | 0.35 ± 0.13 | | 0.18 ± 0.00 | |
| Eos. | 10^9^/L | | 0.09 ± 0.03 | | 0.21 ± 0.05* | | 0.22 ± 0.00 | |
| Baso. | 10^9^/L | | 0.07 ± 0.03 | | 0.07 ± 0.03 | | 0.06 ± 0.00 | |
| LUC | 10^9^/L | | 0.20 ± 0.02 | | 0.17 ± 0.05 | | 0.07 ± 0.00 | |
| Neut. | % | | 23.40 ± 7.33 | | 19.60 ± 2.38 | | 17.30 ± 0.00 | |
| Lymph. | % | | 71.57 ± 7.29 | | 75.03 ± 2.95 | | 77.80 ± 0.00 | |
| Mono. | % | | 2.27 ± 0.12 | | 2.33 ± 0.67 | | 1.70 ± 0.00 | |
| Eos. | % | | 0.63 ± 0.12 | | 1.40 ± 0.26* | | 2.00 ± 0.00 | |
| Baso. | % | | 0.53 ± 0.06 | | 0.53 ± 0.15 | | 0.50 ± 0.00 | |
| LUC | % | | 1.60 ± 0.26 | | 1.13 ± 0.25 | | 0.70 ± 0.00 | |
| PT | seconds | | 21.1 ± 1.6 | | 19.5 ± 0.3 | | 18.4 ± 0.0 | |
| APTT | seconds | | 19.1 ± 2.0 | | 16.8 ± 1.3 | | 20.2 ± 0.0 | |
