# Supplementary material for: Preclinical small molecule WEHI-7326 overcomes drug resistance and elicits response in patient-derived xenograft models of human treatment-refractory tumors
Source: Cell Death Dis. 2021 Mar 12;12(3):268. doi: 10.1038/s41419-020-03269-0 (PMC7955127; doi:10.1038/s41419-020-03269-0)
Supplement: Supplementary file 22 — Table S4 [file 41419_2020_3269_MOESM22_ESM.docx]

**Table S4: Haematology and Coagulation – WEHI-7326 acute toxicity study (day 15, females).**

|  |  | |  | **Females** |  |  |
| --- | --- | --- | --- | --- | --- | --- |
| **Analyte** | **Unit** | | **Group 1 (D-(+)-Glucose, 10mL/kg)** | **Group 2 (WEHI-7326,**  **5mg/kg)** | **Group 3 (WEHI-7326,**  **15mg/kg)** | **Group 3 (WEHI-7326,**  **20mg/kg)** |
| WBC | | 10^9^/L | 8.21 ± 0.59 | 11.28 ± 4.18 | 10.28 ± 0.00 | 7.85 ± 1.07 |
| RBC | | 10^12^/L | 7.32 ± 0.09 | 7.27 ± 0.14 | 7.31 ± 0.00 | 7.27 ± 0.15 |
| HGB | | g/L | 140.7 ± 0.58 | 137.3 ± 4.9 | 136.0 ± 0.0 | 134.3 ± 3.2 |
| HCT | | L/L | 0.46 ± 0.01 | 0.45 ± 0.01 | 0.45 ± 0.00 | 0.46 ± 0.01 |
| MCV | | fL | 62.5 ± 0.8 | 62.3 ± 0.9 | 60.90 ± 0.0 | 63.7 ± 2.5 |
| MCH | | pg | 19.23 ± 0.21 | 18.87 ± 0.45 | 18.70 ± 0.00 | 18.53 ± 0.84 |
| MCHC | | g/L | 307.7 ± 4.7 | 303.0 ± 4.4 | 306.0 ± 0.0 | 290.7 ± 1.5 |
| PLT | | 10^9^/L | 939.3 ± 45.8 | 1080.0 ± 206.7 | 970.0 ± 0.0 | 957.0 ± 63.5 |
| Retc. | | % | 2.94 ± 0.15 | 3.76 ± 0.62 | 4.52 ± 0.00 | 4.75 ± 1.82 |
| Neut. | | 10^9^/L | 1.33 ± 0.24 | 1.29 ± 0.77 | 2.42 ± 0.00 | 0.97 ± 0.12 |
| Lymph. | | 10^9^/L | 6.54 ± 0.45 | 9.38 ± 3.37 | 7.40 ± 0.00 | 6.38 ± 0.87 |
| Mono. | | 10^9^/L | 0.12 ± 0.02 | 0.27 ± 0.04** | 0.15 ± 0.00 | 0.20 ± 0.06 |
| Eos. | | 10^9^/L | 0.08 ± 0.02 | 0.10 ± 0.02 | 0.11 ± 0.00 | 0.15 ± 0.05 |
| Baso. | | 10^9^/L | 0.04 ± 0.0 | 0.07 ± 0.03 | 0.06 ± 0.00 | 0.05 ± 0.03 |
| LUC | | 10^9^/L | 0.10 ± 0.01 | 0.17 ± 0.02** | 0.14 ± 0.00 | 0.11 ± 0.02 |
| Neut. | | % | 16.17 ± 1.99 | 10.80 ± 3.92 | 23.50 ± 0.00 | 12.37 ± 0.32 |
| Lymph. | | % | 79.63 ± 1.95 | 83.53 ± 3.00 | 72.00 ± 0.00 | 81.27 ± 1.63 |
| Mono. | | % | 1.50 ± 0.26 | 2.53 ± 0.65 | 1.50 ± 0.00 | 2.53 ± 0.45 |
| Eos. | | % | 1.07 ± 0.38 | 0.97 ± 0.15 | 1.10 ± 0.00 | 1.80 ± 0.56 |
| Baso. | | % | 0.47 ± 0.12 | 0.60 ± 0.10 | 0.60 ± 0.00 | 0.63 ± 0.25 |
| LUC | | % | 0.90 ± 0.70 | 1.67 ± 0.45 | 1.30 ± 0.00 | 1.37 ± 0.21 |
| PT | | seconds | 24.1 ± 0.7 | 21.9 ± 1.0 | 19.9 ± 0.0 | 21.8 ± 0.8 |
| APTT | | seconds | 11.4 ± 6.9 | 17.2 ± 1.9* | 18.6 ± 0.0 | 17.3 ± 1.0* |
