# Supplementary material for: Preclinical small molecule WEHI-7326 overcomes drug resistance and elicits response in patient-derived xenograft models of human treatment-refractory tumors
Source: Cell Death Dis. 2021 Mar 12;12(3):268. doi: 10.1038/s41419-020-03269-0 (PMC7955127; doi:10.1038/s41419-020-03269-0)
Supplement: Supplementary file 23 — Table S5 [file 41419_2020_3269_MOESM23_ESM.docx]

**Table S5: Biochemistry – WEHI-7326 acute toxicity study (day 15, males).**

|  | |  | **Males** |  |
| --- | --- | --- | --- | --- |
| **Analyte** | **Unit** | **Group 1 (D-(+)-Glucose, 10mL/kg)** | **Group 2 (WEHI-7326,**  **5mg/kg)** | **Group 3 (WEHI-7326,**  **15mg/kg)** |
| ALT | (U/L) | 63 ± 5 | 66 ± 17 | 61 ± 0 |
| Albumin | (g/L) | 34.5 ± 1.5 | 36.6 ± 4.1 | 32.3 ± 0 |
| Albumin/Globulin | Ratio | 1.4 ± 0.2 | 1.4 ± 0.2 | 1.3 ± 0 |
| ALP | (U/L) | 463 ± 53 | 528 ± 112 | 306 ± 0 |
| AST | (U/L) | 106 ± 16 | 114 ± 16 | 108 ± 0 |
| Bilirubin (Total) | (µM) | 1.0 ± 0.4 | 0.4 ± 0.1 | 0.9 ± 0 |
| Calcium | (mM) | 2.53 ± 0.09 | 2.67 ± 0.12 | 2.50 ± 0 |
| Chloride | (mM) | 99.6 ± 1.1 | 102.9 ± 5.3 | 100.4 ± 0 |
| Cholesterol | (mM) | 2.17 ± 0.61 | 1.81 ± 0.18 | 1.65 ± 0 |
| Creatinine | (µM) | 25 ± 2 | 25 ± 5 | 23 ± 0 |
| Globulin | (g/L) | 24.7 ± 2.7 | 25.3 ± 1.6 | 25.8 ± 0 |
| Glucose | (mM) | 7.79 ± 0.27 | 7.70 ± 0.82 | 7.13 ± 0 |
| Phosphate | (mM) | 1.93 ± 0.07 | 2.07 ± 0.24 | 2.07 ± 0 |
| Potassium | (mM) | 5.19 ± 0.05 | 5.23 ± 0.33 | 4.78 ± 0 |
| Protein (Total) | (g/L) | 59.3 ± 2.1 | 61.9 ± 5.8 | 58.1 ± 0 |
| Sodium | (mM) | 138 ± 0 | 143 ± 10 | 138 ± 0 |
| Triglycerides | (mM) | 1.51 ± 1.12 | 1.65 ± 0.18 | 0.97 ± 0 |
| Urea | (mM) | 6.17 ± 1.04 | 6.11 ± 0.96 | 4.02 ± 0 |
