# Supplementary material for: Preclinical small molecule WEHI-7326 overcomes drug resistance and elicits response in patient-derived xenograft models of human treatment-refractory tumors
Source: Cell Death Dis. 2021 Mar 12;12(3):268. doi: 10.1038/s41419-020-03269-0 (PMC7955127; doi:10.1038/s41419-020-03269-0)
Supplement: Supplementary file 24 — Table S6 [file 41419_2020_3269_MOESM24_ESM.docx]

**Table S6: Biochemistry – WEHI-7326 acute toxicity study (day 15, females).**

|  |  |  | **Females** |  |  |
| --- | --- | --- | --- | --- | --- |
| **Analyte** | **Unit** | **Group 1 (D-(+)-Glucose, 10mL/kg)** | **Group 2 (WEHI-7326,**  **5mg/kg)** | **Group 3 (WEHI-7326,**  **15mg/kg)** | **Group 3 (WEHI-7326,**  **20mg/kg)** |
| ALT | (U/L) | 48 ± 8 | 54 ± 11 | 55 ± 0 | 70 ± 9 |
| Albumin | (g/L) | 38.0 ± 0.8 | 37.3 ± 3.1 | 37.7 ± 0 | 36.5 ± 1.1 |
| Albumin/Globulin | Ratio | 1.6 ± 0.1 | 1.6 ± 0.4 | 1.3 ± 0 | 1.3 ± 0.1 |
| ALP | (U/L) | 273 ± 23 | 286 ± 65 | 218 ± 0 | 171 ± 38 |
| AST | (U/L) | 93 ± 12 | 93 ± 13 | 73 ± 0 | 93 ± 14 |
| Bilirubin (Total) | (µM) | 1.0 ± 0.4 | 0.8 ± 0.1 | 1.0 ± 0 | 1.2 ± 0.3 |
| Calcium | (mM) | 2.46 ± 0.04 | 2.59 ± 0.07 | 2.48 ± 0 | 2.71 ± 0.12 |
| Chloride | (mM) | 102.4 ± 1.4 | 102.4 ± 0.5 | 102.5 ± 0 | 100.5 ± 0.7 |
| Cholesterol | (mM) | 1.70 ± 0.18 | 1.98 ± 0.21 | 1.59 ± 0 | 2.16 ± 0.31 |
| Creatinine | (µM) | 25 ± 3 | 30 ± 3 | 27 ± 0 | 38 ± 5** |
| Globulin | (g/L) | 24.0 ± 1.1 | 23.7 ± 3.4 | 28.8 ± 0 | 27.9 ± 1.2 |
| Glucose | (mM) | 7.85 ± 0.59 | 7.57 ± 0.07 | 7.31 ± 0 | 7.29 ± 0.68 |
| Phosphate | (mM) | 1.69 ± 0.02 | 1.75 ± 0.14 | 1.53 ± 0 | 1.85 ± 0.15 |
| Potassium | (mM) | 5.21 ± 0.18 | 5.30 ± 0.14 | 4.53 ± 0 | 4.72 ± 0.20 |
| Protein (Total) | (g/L) | 62.0 ± 0.6 | 61.0 ± 0.4 | 66.5 ± 0 | 64.3 ± 1.2* |
| Sodium | (mM) | 138 ± 2 | 138 ± 1 | 140 ± 0 | 139 ± 1 |
| Triglycerides | (mM) | 1.19 ± 0.45 | 0.86 ± 0.29 | 0.76 ± 0 | 0.96 ± 0.50 |
| Urea | (mM) | 6.46 ± 0.64 | 6.56 ± 1.18 | 6.91 ± 0 | 6.37 ± 0.39 |
