# Supplementary material for: Preclinical small molecule WEHI-7326 overcomes drug resistance and elicits response in patient-derived xenograft models of human treatment-refractory tumors
Source: Cell Death Dis. 2021 Mar 12;12(3):268. doi: 10.1038/s41419-020-03269-0 (PMC7955127; doi:10.1038/s41419-020-03269-0)
Supplement: Supplementary file 25 — Table S7 [file 41419_2020_3269_MOESM25_ESM.docx]

**Table S7: Summary of Clinical Observations and Adverse Events***

| **Group** | **Treatment** | **Clinical Observations** | **Number of animals** |
| --- | --- | --- | --- |
| **1** | **WEHI-7326,**  **7.5 mg/kg** | No remarkable observation | 3/4 |
|  |  | Mild red staining right fore limb | 1/4 |
| **2** | **WEHI-7326,**  **10 mg/kg** | Mild piloerection | 3/3 |
|  |  | Mild red staining of snout, fore limbs and/or head | 3/3 |
|  |  | Urine-stained fur | 1/3 |
| **3** | **WEHI-7326,**  **15 mg/kg** | Mild/moderate piloerection | 4/4 |
|  |  | Mild/moderate hunched posture | 4/4 |
|  |  | Mild red staining of eyes and or head | 2/4 |
|  |  | Discharge both eyes | 1/4 |
|  |  | Urine-stained fur | 1/4 |
|  |  | Mild squinting both eyes | 1/4 |
|  |  | Early termination (StudyDay 9) due to adverse clinical signs and excess body weight loss | 3/4 |
| **4** | **WEHI-7326,**  **30 mg/kg** | Mild piloerection | 4/4 |
|  |  | Mild/moderate hunched posture | 4/4 |
|  |  | Mild/moderate lethargy | 4/4 |
|  |  | Mild/moderate labored breathing | 4/4 |
|  |  | Mild red staining of limbs, eyes, snout and or head | 4/4 |
|  |  | Mild squinting both eyes | 4/4 |
|  |  | Urine-stained fur | 4/4 |
|  |  | Soft feces/diarrhea | 3/4 |
|  |  | Mild excess salivation | 2/4 |
|  |  | Early termination (StudyDay 5) due to adverse clinical signs and excess body weight loss | 4/4 |
| **2** | **WEHI-7326,**  **60 mg/kg** | Found dead immediately after administration | 1/1 |
