# Supplementary material for: Preclinical small molecule WEHI-7326 overcomes drug resistance and elicits response in patient-derived xenograft models of human treatment-refractory tumors
Source: Cell Death Dis. 2021 Mar 12;12(3):268. doi: 10.1038/s41419-020-03269-0 (PMC7955127; doi:10.1038/s41419-020-03269-0)
Supplement: Supplementary file 26 — Supplementary Figures Legends [file 41419_2020_3269_MOESM26_ESM.docx]

**Supplementary Data – Legends for supporting figures, tables and videos**

**Preclinical small molecule WEHI-7326 overcomes drug resistance and elicits response in patient-derived xenograft models of human treatment-refractory tumors**

Christoph Grohmann^1,2^*, Francesca Walker^1,2,3^*, Mark Devlin^4,5^, Meng-Xiao Luo^1,2^, Anderly C. Chüeh^1,2,5^, Judy Doherty^4,5^, François Vaillant^1,2^, Gwo-Yaw Ho^1,2^, Matthew J. Wakefield^1,2,6^, Clare E. Weeden^1,2^, Alvin Kamili^8,9^, Jayne Murray^8^, Sela T Po’uha^8^, Janet Weinstock^1,2,3^, Serena R. Kane^1,2^, Maree C.Faux^1,2^, Esmee Broekhuizen^1,2^, Ye Zheng^1,2^, Kristy Shield-Artin^1,2^, Nadia J. Kershaw^1,2,3^, Chin Wee Tan^1,2^, Helen M. Witchard^1^, Gregor Ebert^1,2^, Susan A. Charman^7^, Ian Street^1,5^, Maria Kavallaris^8,10^, Michelle Haber^8^, Jamie I. Fletcher^8,9^, Marie-Liesse Asselin-Labat^1,2^, Clare L. Scott^1,2,4,6^, Jane E. Visvader^1,2^, Geoffrey J. Lindeman^1,2,4,11^, Keith G. Watson^1,2^, Antony W. Burgess^1,2,3,‡^, Guillaume Lessene^1,2,12,‡^

*: Joint first; ^‡^: joint senior authors

1. Walter and Eliza Hall Institute, Parkville, Victoria 3052, Australia

2. The University of Melbourne, Department of Medical Biology, Parkville, Victoria 3050, Australia

3. Ludwig Institute for Cancer Research, Melbourne, Victoria 3000, Australia

4. Peter MacCallum Cancer Centre, Victorian Comprehensive Cancer Centre building, Melbourne, Australia 3000

5. Cancer Therapeutics CRC, Melbourne, Victoria 3000, Australia

6. The University of Melbourne, Department of Obstetrics and Gynaecology, Parkville, Victoria 3050, Australia

7. Centre for Drug Candidate Optimisation, Monash Institute of Pharmaceutical Sciences, Monash University, Victoria 3052, Australia

8. Children’s Cancer Institute, Lowy Cancer Research Centre, UNSW Sydney, NSW 2052, Australia

9. School of Women’s and Children’s Health, UNSW Sydney, NSW 2052, Australia

10. ARC Centre of Excellence in Convergent Bionano Science and Technology, Australian Centre for Nanomedicine, UNSW Sydney, NSW 2052, Australia

11. The University of Melbourne, Department of Medicine, Parkville, Victoria 3000

12. The University of Melbourne, Department of Pharmacology and Therapeutics, Parkville, Victoria 3050, Australia

**Supporting Figures Legends**

# Figure S1 (related to Figure 1): Optimization of novel class of mitotic inhibitors.

**a** Chemical structures of the triazine-based anti-mitotics used in this study. **b** Dose-dependent antiproliferative activity of key derivatives and comparison with commonly used anti-mitotics in SW480 cell line: Data shown is cell viability (as measured by CellTiterGlo) after 72 h relative to untreated control cells. Shown is mean of 3 independent experiments performed in duplicate +/- SEM. **c** Dose-dependent cell cycle analysis of key derivatives of WEHI-7326 development in SW480 cell line. Data are presented as percentage of cells in G2/M or subG0 after 24 h incubation with titration of inhibitors as assessed by PI staining and flow cytometry. Mean average of 3 experiments +/- SEM. **d** Comparison of IC_50_ and EC_50_ values of triazine compounds and anti-mitotic drugs in SW480 cell line: inhibition of cellular proliferation and cell cycle arrest in G2/M. IC_50_ and EC_50_ values are the mean of 3 independent experiments run in duplicates +/- SEM (n.d. = not determined). **e** HepG2 cytotoxic assay: cell viability assay after 48 h, normalised to 0.4% DMSO (100% viability) and 2 *μ*M bortezomib (0% viability). The data is the mean average of 2 experiments +/- SD.

**Figure S2 (related to Table 1)**: **Comparison of IC_50_ values (nM) for inhibition of cell proliferation.**

WEHI-7326, Paclitaxel, Nocodazol and Myoseverin B were assayed for their anti-proliferative activity in different tumor cell lines: **a** Titration curves for the 4 compounds in MDA-MB-231, H1437, LoVo and PC3 cells in a viability assay using CellTiterGlo reagent as read out at 72 h; curves represent the mean of 3 independent experiments run in duplicates, error bars represent SEM. **b** Summary of IC_50_ values for the data presented in a (IC_50_ +/- SEM).

**Figure S3 (related to Figure 2): Impact of WEHI-7326 on cell cycle distribution and mitotic arrest.**

**a** Cell cycle distribution of SW480 and MDA-MB-231 cancer cells after 24 h treatment with the indicated drugs, as assessed by PI staining and flow cytometry. **b,c** Induction of mitotic arrest by WEHI-7326 in MDA-MB-231 tumour cells measured using high content imaging. **b** Dose-response curve depicting anti-mitotic drug dose-dependent phospho-Histone 3 (pHH3) expression: cells were treated with 9-point 4 fold titration of WEHI-7426 or Nocodazol for 24 hr. **c** Confocal images of cells immune-stained using specific anti-H3S10ph or anti-H2AXph antibodies, followed by fluorescence-tagged secondary antibodies and DAPI (DNA) stain. Shown are representative images of at least 2 independent experiments. Scale bar = 50 *μ*m. **d** Cell cycle analysis of LIM1899 cells 72 h post treatment with increasing concentrations of WEHI-7326 as measured by PI staining for DNA content followed by flow cytometry analysis. Shown are average mean +/- SD of two independent experiment performed in duplicates.

**Figure S4 (related to Figure 2): Induction of apoptosis elicited by WEHI-7326 in cancer cell lines following mitotic arrest.**

**a** Proapoptotic activity elicited by WEHI-7326 in MDA-MB-231 or LIM2537 tumour cells as measured using Annexin V and PI staining, followed by flow cytometry analysis. Cells were treated for 24 h with 6-point 4-fold dose titration of WEHI-7326. Shown are averages of 2 experiments performed in duplicate +/- SD. **b** Western blot analysis of activated (cleaved) caspase-3 in SW480 and MDA-MB-231 cells treated with EC_50_ or 10-fold EC_50_ concentrations of WEHI-7326, at different timepoints post treatment as indicated. Expression of *β*-actin used as a loading control. Positive control: 200 ng/mL tumor necrosis factor (TNF) and 10 *μ*M Birinapant (Smac mimetic).

**Figure S5 (related to Figure 2): Impact of WEHI-7326 on microtubule network in SW480 cells.**

Effect of WEHI-7326 and different anti-mitotic drugs on microtubule network during mitosis: **a** SW480 colon cancer cells were synchronized using a thymidine-block and released, then treated with 1 *μ*M doses of WEHI-7326, myoseverin B, paclitaxel or nocodazol and incubated for 24 h to capture mitotic cells. Scale bar = 20 *μ*m. **b** Asynchronous SW480 cells were incubated for 24 h with the microtubule destabilizing agents myoseverin B or nocodazole, the microtubule stabilizing agent paclitaxel, or WEHI-7326 at 100 nM. Cells were fixed and stained with anti-tubulin antibodies followed by an Alexa 488-coupled secondary antibody (green); DNA was stained with DAPI (blue) and actin cytoskeleton with phalloidin (red). Shown are representative images of single confocal sections and enlarged areas (3x zoom) to highlight changes in microtubule network. Arrows highlight mitotic cells. Scale bar = 20 *μ*m.

**Figure S6 (related to Table 1): Sensitivity of epothilone and paclitaxel cross-resistant cell lines to WEHI-7326.**

Parental human lymphoblastic leukemia cells (CEM) and their drug resistant derivatives (dEpoB30 and dEpoB300) were cultured in the presence of increasing amounts of WEHI-7326, paclitaxel or epothilone B. Cell survival was determined as described in Experimental Procedures. Shown are mean of 3 experiments +/- SEM. Dose response curves were fitted in PRISM using non-linear regression (4 parameter logistic) to calculate IC_50_ values.

**Figure S7:** **Physicochemical and pharmacokinetic characteristics of WEHI-7326.**

**a** Measured physicochemical and solubility data for WEHI-7326. WEHI-7326 is soluble in common dosing vehicles (5% [w/v] glucose, 0.9% [w/v] saline and isotonic acetate buffer pH 5) and stable under these conditions for prolonged period of time with negligible degradation observed during storage. **b** Plasma concentrations in male Swiss outbred mice following i.v. administration via tail vein injection across four dose levels (3, 10, 15 and 30 mg/kg), determined via HPLC-MS (LLQ = lower limit of quantitation). There were no adverse reactions or compound-related side effects at the doses studied. **c** Pharmacokinetic parameters for WEHI-7326 in male Swiss outbred mice following i.v. administration at 15 mg/kg, WEHI-7326 exhibited a half-life of 6.3 h, the apparent volume of distribution was 4.7 L/kg, and the plasma clearance was 53 mL/min/kg. **d** Calculated metabolic stability parameters for WEHI-7326 based on degradation profiles in human, rat and mouse cryopreserved hepatocytes. Microsomal-predicted E_H_ values are included. The extent of protein binding for WEHI-7326 in mouse plasma was determined to be low (61% bound) and WEHI-7326 exhibited comparable low rates of metabolism in human, rat and mouse liver microsomes and cryopreserved hepatocytes, suggesting a low hepatic-mediated clearance *in vivo* of WEHI-7326.

**Figure S8 (related to figure 3): Body weight changes in nude mice carrying cancer cell line xenografts and treated with WEHI-7326.**

**a – c** Cell line xenografts: LIM2537 (colon carcinoma:), U87MG(Δ2-7) (glioblastoma) or H1437 (non-small cell lung carcinoma): Body weights were measured thrice weekly. Data for each point are the mean +/- SEM of eight individual tumors. Black dotted vertical lines indicate start of administration of food ‘mash’ supplement to WEHI-7326 treatment arm to control the weight loss observed in these mice. **d** Cell line xenograft: LoVo (colon carcinoma): Body weights were measured thrice weekly. Data for each point are the mean +/- SEM of five mice. WEHI-7326 was well tolerated over course of the experiment (9 doses in total) and showed only ~6% body weight loss at 25 mg/kg of WEHI-7326. **e** Cell line xenograft: PC3 (prostate carcinoma): Body weights were measured thrice weekly. Data for each point are the mean +/- SEM of ten mice. The PC3 model is of cachexic nature. Vertical blue dotted line in graph show docetaxel treatment. Docetaxel was toxic after 3 doses and mice did not recover from their weight loss until 1 week after last dose (culled). **f** Cell line xenograft: MDA-MB-231 LNA DRE (docetaxel resistant breast): Body weights were measured thrice weekly. Data for each point are the mean +/- SEM of all individual mice (n = 12) in each group. Vertical blue dotted line in graph show docetaxel treatment. Mice treated with docetaxel had to be culled after 2^nd^ dose due to toxicity as demonstrated by drastic weight loss reaching ethical endpoint (20% loss in body wt). All docetaxel and remaining vehicle mice were harvested on day 16 of treatment. WEHI-7326 treated mice showed no signs of toxicity and appeared healthy and put on weight. **g-h** Cell line xenograft: MDA-MB-231 LNA DRE continued tumor growth curve and body weight changes. After vehicle and docetaxel treated mice were culled on day 30 post implantation (day 16 of treatment), WEHI-7326 were continued to be treated with an increased dose of WEHI-7326 (35 mg/kg, i.v.) and monitored 6 more weeks. Upward ticks on x-axis indicate administration points of WEHI-7326. Data for each point are the mean +/- SEM of 7 individual mice until day 57, when 4 mice were culled due to signs of tumor regrowth and sickness. The remaining 3 mice appeared healthy with almost complete tumor regression.

**Figure S9 (related to figure 3): Post mortem analysis of organs harvested from nude mice carrying the human cell line xenografts.**

**a-b** Measured organ weights from mice in either vehicle or WEHI-7326 treatment arms in U87 and H1437 xenograft studies at endpoint of experiment. Mean +/- SD. **c** Images of tumours harvested from individual mice in H1437 lung cancer xenograft study depicting the differences in tumor growth inhibition between vehicle and WEHI-7326 treatment arms. **d** Sizes of individual tumors and spleens harvested from either vehicle or WEHI-7326 treated mice at end of LoVo xenograft study. The increase in spleen weight for the 20 mg/kg group is believed to be due to the mice being dosed 1 h before harvest on day 30 as opposed to 24 h for the 25 mg/kg group. **e** Sizes of individual tumors of vehicle or WEHI-7326 groups in PC3 xenograft study after harvest on day 17. **f** MDA-MB-231 LNA DRE xenograft study: Images of representative mice of docetaxel (red tail band) or WEHI-7326 (blue tail band) taken at day 30, before docetaxel group was culled (they were not recovering from severe weight loss). **g** Histological analysis of LIM2537 xenograft tumors of vehicle- and WEHI-7326 treated animals *post mortem*. Hematoxylin and eosin staining of tumor section (scale 2000 μm) and enlarged view in inset (200 μm) showing tumor cells with necrotic centre. The tumors from WEHI-7326 treated mice show increased cell death compared to vehicle-treated. p-values were generated by one-way ANOVA and unpaired t-tests, p < 0.05 (*), p < 0.01 (**), p < 0.001 (***), p < 0.0001 (****).

**Figure S10 (related to figure 4):** **Body weight changes in taxane-refractory PDX mouse models of triple-negative breast cancer.**

Body weight change (**a – b**) and relative change in body weight compared to day 0 (**c – d**) of 24T TNBC and 322_TNC PDX recipient female mice treated with either docetaxel (10 mg/kg; i.p.; once weekly) or WEHI-7326 (20 mg/kg; twice a week; i.v.). Vehicle mice were administered the vehicle for WEHI-7326. 24T model (n = 7 mice) adverse effects in WEHI- 7326 arm: 1 female sick, 1 female found dead, 1 female with back leg paralysis. 322TNC model (n = 10 mice) adverse effect in WEHI-7326 arm: 1 female sick, 1 female found dead.

**Figure S11 (related to figure 4):** **Effect of WEHI-7326 on TNBC PDX cells *in vivo* - proliferation and induction of apoptosis.**

The effects on cellular (**a**) proliferation and (**b**) apoptosis *in vivo* following 16 h treatment with single dose of WEHI-7326 or Docetaxel was studied in the 24T TNBC PDX model. Tumors (n = 9 tumors each) were harvested and single cell suspensions prepared, stained with fluorescence-conjugated antibodies against Ki67 (proliferation) or cleaved-Caspase 3 (apoptosis) and analyzed via flow cytometry. P-values were generated in PRISM using two-tailed t-tests.

**Figure S12 (related to figure 4):** **Post mortem organ analysis for toxicity of WEHI-7326 *in vivo* following short-time treatment.**

The effects of 16 h treatment with single dose of 20 mg/kg WEHI-7326 on crucial organs (**a**) and spine (**b**) in the 24T TNBC PDX mouse model were assessed using hematoxylin and eosin (H&E) stains. Following 16 h treatment, mice were culled and organs were harvested post mortem and sliced for histology analysis. **a** H&E stain of organs are (A) ovary; (B) uterus; (C) liver; (D) kidney; (E) spleen; (F) heart; (G) lung. **b** H&E of spine (coronal section) with anatomical markers. Scale bar = 200 *μ*m

**Figure S13 (related to figure 5): Response of lung squamous cell carcinoma PDX MH792 to cisplatin.**

Tumor growth curve (**a**) and Kaplan-Meier survival plot (**b**) of MH792 LUSC PDX recipient male mice treated with either cisplatin (4 mg/kg, i.p.; once) or vehicle. p = 0.5071 (ns) compared to vehicle control; p-values obtained through Log-rank (Mantel-Cox) test, n = 7-10 mice per arm.

**Figure S14 (related to figure 6): Drug responses in chemo-naïve and body weight changes in all PDX mouse models of high-grade serous ovarian cancer.**

**a** Table summarizing the responses of the chemo-naïve HG-SOC PDX models to cisplatin. The calculation of time to progressive disease (PD) and median time to harvest (TTH) are based on ^1^ **b** Table summarizing the responses of the chemo-naïve HG-SOC PDX models WEHI-7326. **c** Relative changes in body weight compared to day 0 of HG-SOC PDX recipient female mice treated with either vehicle or WEHI-7326 (20 mg/kg; twice a week for 6 weeks; i.v.). Body weights were measured when treatment was administered (12 measurement points for each mouse) but not continued after completion of treatment. Vehicle control administered was vehicle for WEHI-7326 (5% dextrose in saline).

**Figure S15 (related to figure 6): Responses to WEHI-7326 in post-chemo PDX mouse models of very aggressive high-grade serous ovarian cancer.**

**a – c** Tumor growth curves and Kaplan-Meier survival plots of Different post-taxane treated and platinum-refractory PDX models of HG-SOC in female recipient mice: 134111 (CCNE1 amplification), 134169 (BRCA1 methylated) and 34931 (cascade PDX, refractory to SOC treatment except MTA) treated with either vehicle or WEHI-7326 (20mg/kg; twice a week for 6 weeks; i.v.). Ethical endpoint was tumor size >700 mm^3^, censored data when mice culled not due to tumor size (illness, weight loss). Displayed are *p*-values compared to vehicle obtained through Log-rank (Mantel-Cox) test. **d – e** Table summarizing the responses of the post-chemo HG-SOC PDX models to cis-platin (**d**) or WEHI-7326 (**e**). The calculation of time to progressive disease (PD) and median time to harvest (TTH) are based on literature^1^**.**

**Figure S16 (related to figure 7): The corresponding weight data (showing tolerability) for the high-risk neuroblastoma PDX models and the Th-MYCN transgenic mouse model.**

Body weight changes of mice during treatment period for PDX model COG-N-496x (**a**), COG-N-440x (**b**) and Th-MYCN transgenic mouse model (**c**). Shown are averages +/- SD. In all animal experiments for high risk neuroblastoma, mice were euthanized upon detection of an abdominal tumour of 1 cm in diameter. Mice were also euthanized if showing weight loss equal to or greater than 20%. **d** The Th-MYCN transgenic mouse model of MYCN-amplified neuroblastoma as reported ^2^: Human MYCN is under control of neural crest (Th) promoter, spontaneous neuroblastomas arise in paraspinal ganglia with 100% incidence and very short latency (all mice culled by 7 weeks of age).

**Figure S17 (related to figure 7): The corresponding individual mouse tumor volume and weight data for the neuroblastoma models.**

Individual tumor volumes and body weight changes of mice during treatment period for PDX model COG-N-496x (**a, b**), COG-N-440x (**c,d**) and Th-MYCN transgenic mouse model (**e**). In all animal experiments for high risk neuroblastoma, mice were euthanized upon detection of an abdominal tumour of 1 cm in diameter. Mice were also euthanized if showing weight loss equal to or greater than 20%.

**Figure S18 (related to supporting tables S1-S7): Mean body weight changes for toxicity studies in Sprague-Dawley rats.**

**a** Body weight progression for acute toxicity study following single intravenous dose administration of WEHI-7326 in Sprague-Dawley rats. Given are mean averages +/- SD (n = 3 mice per group). **b** Mean body weight progression during the fourteen day maximum tolerated dose finding study following twice weekly intravenous administration with WEHI-7326 in female Sprague-Dawley rats is presented. Given are mean averages +/- SD (n = 4 mice per group).

**Supporting Tables Legends**

**Table S1: Summary of Clinical Observations and Adverse Events – WEHI-7326 acute toxicity study following single intravenous dose administration in male and female Sprague-Dawley rats.**

Two animals (one male animal in Group 3 Toxicity Study and one male animal in Group 4 Toxicokinetic Study) required early termination due to laboured breathing, severely affected gait and lack of response to stimuli immediately post-dose. Four animals (one female animal in Group 4 and three male animals in Group 4) showed mild impairment of gait and lack of response to stimuli post-dose which was resolved within 2 hours. An additional six animals required early termination from the study due to reaching their body weight loss endpoint of >10% (one male from Group 3, three males from Group 4 and two females from Group 3). One male in the mid-dose WEHI-7326 (Group 3) had a red-stained right eye prior to it reaching its weight loss endpoint. The two females in the mid-dose WEHI-7326 (Group 3) had no other adverse clinical signs with their weight loss. For the three males in the high-dose WEHI-7326 (Group 4) the loss of body weight was accompanied by a hunched posture with half shut eyes and discharge from the nose. One female in the high-dose WEHI-7326 (Group 4) had mild fur loss around both eyes. No adverse clinical signs were observed in animals treated with Vehicle Control (Group 1) and low dose WEHI-7326 (Group 2). (*) Note: N=4 due to transfer of one male rat from Toxicokinetic Study.

**Table S2: Macroscopic Pathology Summary– WEHI-7326 acute toxicity study.**

The incidence of macroscopic pathology findings at necropsy in each group (males and females) at the end of the study (Study Day 15) following single intravenous dose administration of WEHI-7326 in male and female Sprague-Dawley rats are shown.

**Table S3: Haematology and Coagulation – WEHI-7326 acute toxicity study (day 15, males).**

Mean ±SD is shown for each parameter for Toxicity Study males for each group on Study Day 15 following single intravenous dose administration of WEHI-7326 in male Sprague-Dawley rats. Abbreviations: WBC; white blood cells, RBC; red blood cells, HGB; hemoglobin, HCT; hematocrit, MCV; mean cell volume, MCH; mean cell hemoglobin, MCHC; mean cell hemoglobin concentration, PLT; platelets, LUC; large unstained cells, PT; prothrombin time, APTT; activated partial thromboplastin time. *P<0.05 versus Group 1 (One-Way ANOVA, Dunnett’s Multiple Comparison Test). **P<0.01 versus Group 1 (One-Way ANOVA; Dunnett’s Multiple Comparison Test)

**Table S4: Haematology and Coagulation – WEHI-7326 acute toxicity study (day 15, females).**

Mean ±SD is shown for each parameter for Toxicity Study males for each group on Study Day 15 following single intravenous dose administration of WEHI-7326 in female Sprague-Dawley rats. Abbreviations: WBC; white blood cells, RBC; red blood cells, HGB; hemoglobin, HCT; hematocrit, MCV; mean cell volume, MCH; mean cell hemoglobin, MCHC; mean cell hemoglobin concentration, PLT; platelets, LUC; large unstained cells, PT; prothrombin time, APTT; activated partial thromboplastin time. *P<0.05 versus Group 1 (One-Way ANOVA, Dunnett’s Multiple Comparison Test). **P<0.01 versus Group 1 (One-Way ANOVA; Dunnett’s Multiple Comparison Test)

**Table S5: Biochemistry – WEHI-7326 acute toxicity study (day 15, males).**

Mean ±SD is shown for each parameter for Toxicity Study males for each group on Study Day 15 following single intravenous dose administration of WEHI-7326 in male Sprague-Dawley rats. Abbreviations: ALT; alanine amino transferase, ALP; alkaline phosphatase, AST; aspartate amino transferase.

**Table S6: Biochemistry – WEHI-7326 acute toxicity study (day 15, females).**

Mean ±SD is shown for each parameter for Toxicity Study males for each group on Study Day 15 following single intravenous dose administration of WEHI-7326 in female Sprague-Dawley rats. Abbreviations: ALT; alanine amino transferase, ALP; alkaline phosphatase, AST; aspartate amino transferase.

**Table S7: Summary of Clinical Observations and Adverse Events – WEHI-7326 maximum tolerated dose study.**

Fourteen day maximum tolerated dose (MTD) study of WEHI-7326 dihydrochloride following twice weekly intravenous administration in female Sprague-Dawley rats. All treatments were administered i.v. (tail vein) in a dosing volume of 10 mL/kg. Treatment was administered in a staggered manner, beginning with WEHI-7326 at 7.5 mg/kg and then increasing the dose on subsequent days. For all data calculations and presentation, the first day of treatment for each group is designated as Day 1. One animal died immediately following a single dose of WEHI-7326 at 60 mg/kg (Group 2). The remaining scheduled treatments at this dose were cancelled and dosing recommenced at a reduced dose of 10 mg/kg to the remaining 3 animals in this group. Intravenous (tail vein) treatment of female Sprague-Dawley rats with WEHI-7326 was tolerated without serious adverse events or body weight loss exceeding the ethical limit at doses of 7.5 and 10 mg/kg in this study. As signs of toxicity were observed at doses of 15, 30 and 60 mg/kg, the maximum tolerated dose was determined to be 10 mg/kg.

**Supporting Videos Legends**

**Supplementary Videos S1 and S2: Induction of apoptosis in SW480-H2B-Scarlet cells by WEHI-7326.**

IncuCyte® S3 live-cell analyses of SW480-H2B-Scarlet-1 cells treated with WEHI-7326: SW480-H2B-mScarlet-I cells were incubated overnight in culture medium. The cells were then synchronized in S-phase by a double thymidine block. After the second thymidine block, DMSO control (**Video 1**) or WEHI-7326 (1 *µ*M) (**Video 2**) and SYTOX® green (30 nM) were added to the cultures. Within two hours, the cells were imaged by the IncuCyte® S3 Live-Cell Analysis System. Images were collected every 10 minutes for 24 hours. Movies were generated using the IncuCyte® S3 software. Three image channels (green, red and phase) and masks were layered for the image view. The movies were acquired immediately (0d 0h 0m) after placing the 96-well-plate into the IncuCyte® S3 instrument. The movies were customized to 1 frame per second. Scale bar: 200 *µ*m.

**References**

1 Topp, M. D. *et al.* Molecular correlates of platinum response in human high-grade serous ovarian cancer patient-derived xenografts. *Mol Oncol* **8**, 656-668, (2014).

2 Weiss, W. A., Aldape, K., Mohapatra, G., Feuerstein, B. G. & Bishop, J. M. Targeted expression of MYCN causes neuroblastoma in transgenic mice. *Embo J* **16**, 2985-2995, (1997).
