# Supplementary material for: Preclinical small molecule WEHI-7326 overcomes drug resistance and elicits response in patient-derived xenograft models of human treatment-refractory tumors
Source: Cell Death Dis. 2021 Mar 12;12(3):268. doi: 10.1038/s41419-020-03269-0 (PMC7955127; doi:10.1038/s41419-020-03269-0)
Supplement: Supplementary file 27 — Supplementary Information - In vivo toxicity [file 41419_2020_3269_MOESM27_ESM.docx]

**Supplementary Data – *In vivo* rat non-GLP toxicity studies**

**Preclinical small molecule WEHI-7326 overcomes drug resistance and elicits response in patient-derived xenograft models of human treatment-refractory tumors**

Christoph Grohmann^1,2^*, Francesca Walker^1,2,3^*, Mark Devlin^4,5^, Meng-Xiao Luo^1,2^, Anderly C. Chüeh^1,2,5^, Judy Doherty^4,5^, François Vaillant^1,2^, Gwo-Yaw Ho^1,2^, Matthew J. Wakefield^1,2,6^, Clare E. Weeden^1,2^, Alvin Kamili^8,9^, Jayne Murray^8^, Sela T Po’uha^8^, Janet Weinstock^1,2,3^, Serena R. Kane^1,2^, Maree C.Faux^1,2^, Esmee Broekhuizen^1,2^, Ye Zheng^1,2^, Kristy Shield-Artin^1,2^, Nadia J. Kershaw^1,2,3^, Chin Wee Tan^1,2^, Helen M. Witchard^1^, Gregor Ebert^1,2^, Susan A. Charman^7^, Ian Street^1,5^, Maria Kavallaris^8,10^, Michelle Haber^8^, Jamie I. Fletcher^8,9^, Marie-Liesse Asselin-Labat^1,2^, Clare L. Scott^1,2,4,6^, Jane E. Visvader^1,2^, Geoffrey J. Lindeman^1,2,4,11^, Keith G. Watson^1,2^, Antony W. Burgess^1,2,3,‡^, Guillaume Lessene^1,2,12,‡^

*: Joint first; ^‡^: joint senior authors

1. Walter and Eliza Hall Institute, Parkville, Victoria 3052, Australia

2. The University of Melbourne, Department of Medical Biology, Parkville, Victoria 3050, Australia

3. Ludwig Institute for Cancer Research, Melbourne, Victoria 3000, Australia

4. Peter MacCallum Cancer Centre, Victorian Comprehensive Cancer Centre building, Melbourne, Australia 3000

5. Cancer Therapeutics CRC, Melbourne, Victoria 3000, Australia

6. The University of Melbourne, Department of Obstetrics and Gynaecology, Parkville, Victoria 3050, Australia

7. Centre for Drug Candidate Optimisation, Monash Institute of Pharmaceutical Sciences, Monash University, Victoria 3052, Australia

8. Children’s Cancer Institute, Lowy Cancer Research Centre, UNSW Sydney, NSW 2052, Australia

9. School of Women’s and Children’s Health, UNSW Sydney, NSW 2052, Australia

10. ARC Centre of Excellence in Convergent Bionano Science and Technology, Australian Centre for Nanomedicine, UNSW Sydney, NSW 2052, Australia

11. The University of Melbourne, Department of Medicine, Parkville, Victoria 3000

12. The University of Melbourne, Department of Pharmacology and Therapeutics, Parkville, Victoria 3050, Australia

**Key findings – In vivo toxicity studies WEHI-7326**

**1) Acute Toxicity Study of WEHI-7326 Dihydrochloride Following Single Intravenous Dose Administration in Male and Female Sprague-Dawley Rats (non GLP)**

**Aim.** This study was performed to assess the acute toxicity of WEHI-7326 following a single intravenous dose administration in male and female Sprague Dawley rats.

**Summary**

This acute toxicity study included a satellite group for toxicokinetic assessment. The toxicity study was comprised of 12 male and 12 female rats, which were allocated into four groups of three males and three females. The toxicity of the test substance, WEHI-7326 dihydrochloride (referred to as WEHI-7326 from here), was tested at three dose concentrations of 5 mg/kg, 15 mg/kg and 30 mg/kg administered as a single dose via intravenous tail vein injection. The vehicle control, D-(+)-Glucose, was formulated at 5% w/v and administered per the above regime. On Study Day 15, all animals were terminated with blood collected for biochemistry, haematology and coagulation analysis before undergoing gross necropsy by a US board certified veterinary pathologist. The toxicokinetic satellite group comprised of 30 male rats which were allocated into four groups of three males in the vehicle control group and nine males in each of the three treatment groups. All treatment group animals were then allocated into three subgroups (A, B and C) and bled pre-dose, then 2min, 10min, 0.5h, 2h, 6h, 12h and 24h post-dose. All vehicle control group animals weren’t allocated to a specific subgroup but were bled at 10min and 12h post-dose. All TK animals were then terminated after the final blood collection. Dosing was suspended on Study Day 1 for both the Toxicity and Toxicokinetic Study animals following adverse reactions which lead to the early termination of two animals (one male treated with mid-dose WEHI-7326 from the Toxicity Study and one male treated with high-dose WEHI-7326 from the Toxicokinetic Study) approximately 5-10 minutes post-treatment. After consultation with the Sponsor, the following changes were made to the Study Design:

1. One rat from Group 4 TK Study was randomly selected and added to the Group 3 Toxicity Study to replace the one male rat that required euthanasia.

2. All Group 3 Toxicity Study rats were then administered a dose (i.v.) of 15mg/kg WEHI-7326, starting with the female rats.

3. Group 3 Toxicity Study rats were monitored closely over a 48 hour period whereby all rats had tolerated the dose well and did not exhibiting any adverse signs to suggest euthanasia was required. Group 4 Toxicity Study rats were then administered a dose (i.v.) of 20mg/kg WEHI-7326, again starting with the female rats.

4. As for Group 3 Toxicity Study rats, Group 4 rats were closely monitored and assessed for adverse signs immediately post-dose and for the remainder of the study duration.

Dosing of remaining Group 4 Toxicokinetic Study rats was not performed.

Treatment of Toxicity Study rats with WEHI-7326 at 15 mg/kg resulted in the premature termination of two male and two female rats due to reaching their body weight loss endpoint of >10% loss. At the revised high dose of 20mg/kg, all male rats had to be terminated prematurely also due to reaching their body weight loss endpoint of >10% loss. Some signs of toxicity were observed in rats treated at 20 mg/kg immediately post-dose (mild impairment of gait, lack of arousal), whereby one female rat recovered after one hour post-dose and other affected male rats having to be culled due to reaching body weight loss endpoints. Significant findings in clinical pathology outcomes were considered incidental and unrelated to test article treatment. The majority of macroscopic findings at necropsy were also considered to be incidental, however the findings of white streaks through the testes of male animals treated with WEHI-7326 may be related to treatment. Body weights were measured once prior to dose administration, then daily for the duration of the study and at termination. Clinical observations were performed on a daily basis.

The study followed test guideline OECD No. 420 (2001) “Acute Oral Toxicity - Fixed Dose Procedure” with the exception that the route of administration was intravenous (i.v.).

This study was a non-GLP study.

**1.1 Key findings - Results and Discussion**

**1.1.1** **Clinical Observations and Adverse Events**

The overall incidence of clinical signs is shown in Table S1. Two animals (one male animal in Group 3 Toxicity Study and one male animal in Group 4 Toxicokinetic Study) required early termination due to laboured breathing, severely affected gait and lack of response to stimuli immediately post-dose. Four animals (one female animal in Group 4 and three male animals in Group 4) showed mild impairment of gait and lack of response to stimuli post-dose which was resolved within 2 hours. An additional six animals required early termination from the study due to reaching their body weight loss endpoint of >10% (one male from Group 3, three males from Group 4 and two females from Group 3). One male in the mid-dose WEHI-7326 (Group 3) had a red-stained right eye prior to it reaching its weight loss endpoint. The two females in the mid-dose WEHI-7326 (Group 3) had no other adverse clinical signs with their weight loss. For the three males in the high-dose WEHI-7326 (Group 4) the loss of body weight was accompanied by a hunched posture with half shut eyes and discharge from the nose. One female in the high-dose WEHI-7326 (Group 4) had mild fur loss around both eyes.

No adverse clinical signs were observed in animals treated with Vehicle Control (Group 1) and low dose WEHI-7326 (Group 2).

**1.1.2 Body Weight Changes**

Mean body weight progression for male and female rats are shown in Supporting Figure S18. Animals in the mid- and high-dose groups treated with 15 and 20 mg/kg WEHI-7326 respectively (Groups 3 and 4) showed an initial decrease in body weight for up to 2-3 days post-dose. Animals that had an acute weight loss of >10% were terminated early, whilst the remaining animals showed a recovery and gained weight over the remainder of the study.

**1.1.3 Necropsy**

The incidence of macroscopic pathology findings detected in male and female rats at necropsy are shown in Table S2. In male animals dosed with WEHI-7326 at 5 and 15 mg/kg (Groups 2 and 3), white streaks though one or both testes were observed. There were incidental findings in a small number of animals in the lungs (petechiae, red or haemorrhagic areas) and liver (pale yellow).\

**1.1.4 Haematology and Coagulation**

Mean haematology and coagulation parameters for male and female rats are shown in Tables S3 and S4. Eosinophil levels were significantly (P<0.05) elevated in males treated with low-dose WEHI-7326 (Group 2) compared to control (Group 1). Monocyte levels were significantly elevated (p<0.01) in females treated with low-dose WEHI-7326 (Group 2) which was not significant at higher doses of WEHI-7326 when compared to control (Group 1). Females treated with low-dose WEHI-7326 (Group 2) also had a significantly increased level of large unstained cells (LUC) (p<0.01) not present in other treatment groups. Activated partial thromboplastin time (APTT) was significantly increased (p<0.05) in females treated with low- and high-dose WEHI-7326 compared to control (Group 1).

**1.1.5 Biochemistry**

Mean biochemistry parameters for male and female rats are shown in Table S5 and S6. Males treated with WEHI-7326 had no significant changes in blood biochemistry. Females treated with high-dose WEHI-7326 had a significantly higher level of total protein (p<0.05) compared to control (Group 1) and a significantly higher amount of creatinine (p<0.01) compared to control (Group 1)

**1.2 Conclusion**

The acute toxicity of a single intravenous (i.v.) dose of WEHI-7326 Dihydrochloride at dose levels of 5, 15 and 20 mg/kg was assessed in male and female Sprague-Dawley rats. WEHI-7326 Dihydrochloride administered at 5 mg/kg was well tolerated in this study. At 15 and 20 mg/kg, early terminations were required due to excessive body weight loss beyond specified endpoint (>10%) in two male and two female animals at 15 mg/kg and all male animals at 20 mg/kg. Adverse clinical signs (laboured breathing, severely affected gait and lack of response to stimuli) immediately post-dose in one male Toxicity Study animal dosed at 15 mg/kg and one Toxicokinetic Study animal dosed at 30 mg/kg also required early intervention and euthanasia. Significant findings in clinical pathology outcomes were considered incidental and unrelated to test article treatment. The majority of macroscopic findings at necropsy were also considered to be incidental, however the findings of white streaks through the testes of male animals treated with WEHI-7326 Dihydrochloride may be related to treatment.

**2) Fourteen Day Maximum Tolerated Dose (MTD) Study of WEHI-7326 Dihydrochloride Following Twice Weekly Intravenous Administration in Female Sprague-Dawley Rats (non-GLP)**

**Aim.** The purpose of the study was to determine the maximum tolerated dose (MTD) of WEHI-7326 Dihydrochloride (WEHI-7326) following intravenous administration in Sprague-Dawley rats, a commonly used and accepted species for this test. In this 14 day dose finding study, the MTD of the test article WEHI-7326 was investigated using female Sprague-Dawley rats. The dose selected was based on the National Centre for the Replacement, Refinement and Reduction of Animals in Research (NC3Rs)’s “Guidance on dose level selection for regulatory general toxicology studies for pharmaceuticals”. This study is non-GLP.

**Summary**

Sixteen female Sprague-Dawley rats were microchipped and randomised into four groups of four based on body weight two days prior to the first day’s treatment. The test article was to be tested at four dose concentrations of 7.5 mg/kg, 15 mg/kg, 30 mg/kg and 60 mg/kg, administered i.v. via tail vein injection at a dose rate of 10 mL/kg. Dosing was staggered between each dose level group and animals were observed for any adverse effects. Animals were to be dosed on their respective Study Day 1, then twice weekly over two weeks. Body weights were measured once prior to dose administration, then daily for the duration of the study and at termination. Clinical observations were performed on a daily basis. Due to the immediate death of one animal dosed with WEHI-7326 at 60 mg/kg and subsequent development of signs of toxicity in animals that had received three doses at 15 mg/kg and two doses at 30 mg/kg, the dose level for the remaining three animals in Group 4 was reduced to 10 mg/kg (four doses). As treatment with WEHI-7326 was tolerated without serious adverse events or body weight loss exceeding the ethical limit at doses of 7.5 and 10 mg/kg in this study, the maximum tolerated dose was determined to be 10 mg/kg.

**2.1 Key findings – Results and discussion**

**2.1.1 Clinical Observations and Adverse Events**

Clinical observations and adverse events during the study are presented in Table S7.

Apart from one observation of mild red staining of the fur on the right forelimb, there were noadverse clinical signs in animals treated with the lowest dose of WEHI-7326 Dihydrochloride (7.5 mg/kg, Group 1). All animals receiving WEHI-7326 Dihydrochloride at 15 mg/kg (Group 2) presented with mild to moderate piloerection and hunched posture. Three animals additionally showed one or more of redstaining of the eyes and or head, discharge from both eyes, urine-stained fur and squinting of both eyes. Three of the animals in this group were culled after three doses had been administered, on Study Day 9, as the severity of the clinical condition and body weight loss exceeded the ethical limits. All animals receiving WEHI-7326 Dihydrochloride at 30 mg/kg (Group 3) presented with mild to moderate piloerection, hunched posture, lethargy, laboured breathing, red staining of limbs, eyes, snout and or head, squinting of both eyes and urine-stained fur. Two animals additionally showed signs of diarrhoea and excess salivation. All of the animals in this group were culled after two doses had been administered, on Study Day 5, as the severity of the clinical condition and body weight loss exceeded the ethical limits. The first animal treated with WEHI-7326 Dihydrochloride at 60 mg/kg (Group 4) died immediately following treatment. Mild signs of red staining of the snout, fore limbs and/or head were observed in the remaining three animals of this group treated with the reduced dose of WEHI-7326 Dihydrochloride at 10 mg/kg. One animals also presented with urine-stained fur.

**2.1.2 Body Weight Changes**

No animals receiving WEHI-7326 Dihydrochloride at 7.5 mg/kg (Group 1) and 10 mg/kg (Group 4) showed body weight loss exceeding the ethical limit (Figure S18). Three animals receiving WEHI-7326 Dihydrochloride at 15 mg/kg (Group 2) were culled on Study Day 9 and three receiving WEHI-7326 Dihydrochloride at 30 mg/kg (Group 3) were culled on Study Day 5 as body weight loss exceeded the ethical limit of 15%.

**2.2 Conclusion**

Intravenous (tail vein) treatment of female Sprague-Dawley rats with WEHI-7326 Dihydrochloride was tolerated without serious adverse events or body weight loss exceeding the ethical limit at doses of 7.5 and 10 mg/kg in this study.

As signs of toxicity were observed at doses of 15, 30 and 60 mg/kg, the maximum tolerated dose was determined to be 10 mg/kg.
