# Supplementary material for: Preclinical small molecule WEHI-7326 overcomes drug resistance and elicits response in patient-derived xenograft models of human treatment-refractory tumors
Source: Cell Death Dis. 2021 Mar 12;12(3):268. doi: 10.1038/s41419-020-03269-0 (PMC7955127; doi:10.1038/s41419-020-03269-0)
Supplement: Supplementary file 28 — Supplementary Experimental Information [file 41419_2020_3269_MOESM28_ESM.docx]

**Supplementary Experimental Procedures**

**Preclinical small molecule WEHI-7326 overcomes drug resistance and elicits response in patient-derived xenograft models of human treatment-refractory tumors**

Christoph Grohmann^1,2^*, Francesca Walker^1,2,3^*, Mark Devlin^4,5^, Meng-Xiao Luo^1,2^, Anderly C. Chüeh^1,2,5^, Judy Doherty^4,5^, François Vaillant^1,2^, Gwo-Yaw Ho^1,2^, Matthew J. Wakefield^1,2,6^, Clare E. Weeden^1,2^, Alvin Kamili^8,9^, Jayne Murray^8^, Sela T Po’uha^8^, Janet Weinstock^1,2,3^, Serena R. Kane^1,2^, Maree C.Faux^1,2^, Esmee Broekhuizen^1,2^, Ye Zheng^1,2^, Kristy Shield-Artin^1,2^, Nadia J. Kershaw^1,2,3^, Chin Wee Tan^1,2^, Helen M. Witchard^1^, Gregor Ebert^1,2^, Susan A. Charman^7^, Ian Street^1,5^, Maria Kavallaris^8,10^, Michelle Haber^8^, Jamie I. Fletcher^8,9^, Marie-Liesse Asselin-Labat^1,2^, Clare L. Scott^1,2,4,6^, Jane E. Visvader^1,2^, Geoffrey J. Lindeman^1,2,4,11^, Keith G. Watson^1,2^, Antony W. Burgess^1,2,3,‡^, Guillaume Lessene^1,2,12,‡^

*: Joint first; ^‡^: joint senior authors

1. Walter and Eliza Hall Institute, Parkville, Victoria 3052, Australia

2. The University of Melbourne, Department of Medical Biology, Parkville, Victoria 3050, Australia

3. Ludwig Institute for Cancer Research, Melbourne, Victoria 3000, Australia

4. Peter MacCallum Cancer Centre, Victorian Comprehensive Cancer Centre building, Melbourne, Australia 3000

5. Cancer Therapeutics CRC, Melbourne, Victoria 3000, Australia

6. The University of Melbourne, Department of Obstetrics and Gynaecology, Parkville, Victoria 3050, Australia

7. Centre for Drug Candidate Optimisation, Monash Institute of Pharmaceutical Sciences, Monash University, Victoria 3052, Australia

8. Children’s Cancer Institute, Lowy Cancer Research Centre, UNSW Sydney, NSW 2052, Australia

9. School of Women’s and Children’s Health, UNSW Sydney, NSW 2052, Australia

10. ARC Centre of Excellence in Convergent Bionano Science and Technology, Australian Centre for Nanomedicine, UNSW Sydney, NSW 2052, Australia

11. The University of Melbourne, Department of Medicine, Parkville, Victoria 3000

12. The University of Melbourne, Department of Pharmacology and Therapeutics, Parkville, Victoria 3050, Australia

**Supplementary Materials and Methods -** **IncuCyte® S3 live-cell analyses**

**Cell lines and plasmids:**

SW480 and SW480-H2B- mScarlet-I cell lines were passaged in RPMI medium and 1% (v/v) ADDS with 10% (v/v) fetal calf serum (FCS).

**H2B-mScarlet-I plasmid** was a gift from the Blewitt laboratory WEHI, consisting of the MSCV-puro-hH2B backbone and a pmScarlet-i_C1 construct (Addgene, Cat# 85044)^1^.

**Culture reagents and buffers:**

**ADDS:** The solution 1% (v/v) was prepared by diluting 10 *µ*M Thioglycerol (Sigma-Aldrich, Cat# 6145), 12.5U Insulin (Actrapid®, Novo Nordisk) and 0.5 mg Hydrocortisone (Pfizer, Cat# G2957) into 500 mL of culture medium. This solution was stored as a 5 mL aliquot working solution at 4°C.

**2XHEBS:** The solution was adjusted to a pH of 6.95-7.05, containing 0.28 M NaCl, 0.05 M HEPES and 1.5 mM Na_2_HPO_4_. The stock solution was sterilized by a 0.22 *µ*m PES syringe filter disc and stored at 4°C in a fridge.

**Generation of** **SW480****-****H2B-mScarlet-I cells by retroviral infection**

The human embryonic kidney (HEK293T) cells (obtained from ATCC) were seeded at 1.5 x 10^6^ – 2 x 10^6^ cells per dish in a 10 cm Petri dish (Corning, Cat# CLS430293) and incubated at 37°C until showing a 70% - 80% confluence. To prepare the retroviral infection mixture, 10 *µ*g of the H2B-mScarlet-I plasmid was packaged with 5 *µ*g of the MSCV viral packaging gag/pol plasmid (Addgene, Cat# 14887) and 3 *µ*g of the pCMV-VSV-G plasmid (Addgene, Cat# 8454) as retroviral plasmid *DNA*^2^ in 500 *µ*L 2XHEBS. This retroviral mixture was then transferred to a sterilised spin-X centrifuge tube filter (Corning, Cat# 01219000) and centrifuged at 20,000xg for 1 minute. The viral pellet was resuspended in 0.5 M CaCl_2_ (250 *µ*L) added to the cell culture. The cells were incubated in a humidified incubator at 37°C for 8 hours before replacing the viral culture medium with 10% FCS and DMEM medium and continuing the incubation at 37°C for a further 2 days. This viral supernatant was harvested by centrifuge at 21,100xg for 5 minutes and sterilised using a 0.45 *µ*m filter (Sartorius, Cat# 90624103). The retroviral supernatant fluid was stored in a -80 °C freezer in 1 mL aliquots.

The parental SW480 cells were seeded at 40,000 cells per well into a 12-well-plate (Corning, Cat#3513) and incubated at 37°C overnight. The culture media was discarded and replaced with 1 mL per well of culture medium containing 4 *µ*g of polybrene (Sigma-Aldrich, Cat# H9268-5G) and 1 mL of the thawed retroviral aliquot. The plates were sealed with parafilm and centrifuged at 600xg, at 32°C, for 45 minutes. After the spin-transduction, the parafilm was removed and the plate incubated at 37°C, for 48 hours.

**Enrichment of the** **SW480-H2B- mScarlet-I cells:** To produce the stable SW480-H2B-mScarlet-I cell line**,** the viral infection medium was discarded and the cells were washed twice with fresh culture media before selecting in 2 mL of medium with puromycin (Sigma-Aldrich, Cat# P9620) (5 µg/mL) at 37°C for 7 days.

**IncuCyte® S3 live-cell analyses of SW480-H2B-Scarlet-1 cells treated with WEHI-7326:** SW480-H2B-mScarlet-I cells were plated in a 96-well-plate (Falcon, Cat# 353219) in a density of 3000 cells per well and incubated overnight in culture medium. The cells were then synchronized in S-phase by a double thymidine block (2 mM, Sigma-Aldrich, Cat# T1895)^3^. After the second thymidine block, DMSO control or WEHI-7326 (1 *µ*M) and SYTOX® green (30 nM) (Molecular Probes, Cat# S-7020) were added to the cultures. Within two hours, the cells were imaged by the IncuCyte® S3 Live-Cell Analysis System (Sartorius, Germany) with the 20X objective lens in three positions for each well. Images were collected every 10 minutes for 24 hours. Movies were generated using the IncuCyte® S3 software. The Green channel, Red channel and Phase channel were selected for imaging and the Colour Autoscale was selected. For the Phase channel, the brightness was adjusted as zero and the contrast was adjusted as -3. The Green Autoscale and the Red Autoscale settings were on. The green fluorophores were set to no spectral unmixing and 7 % of the red channel was removed from the green channel. The movies were defined “As Displayed” for export. Three image channels (green, red and phase) and masks were layered for the image view. There was no scaling or cropping of the images. The movies were acquired immediately (0d 0h 0m) after placing the 96-well-plate into the IncuCyte® S3 instrument. The movies were customized to 1 frame per second. Scale bar: 200 *µ*m.

**Supplementary Experimental Data – Chemistry**

**General chemistry**

Solvents were obtained commercially (SIGMA-ALDRICH) and used without further purification. All non-aqueous reactions were performed in oven-dried glassware under inert atmosphere, unless otherwise specified. Analytical thin-layer chromatography was performed on silica gel ^60^F_254_ aluminum-backed plates (MERCK MILLIPORE) and were visualized by fluorescence quenching under UV light or by KMnO_4_ staining. Chromatography was performed with silica gel 60 (particle size 0.040 – 0.063 μm) using an automated purification system (ISCO TELEDYNE). NMR spectra were recorded on an Agilent MR400 400 MHz or an Agilent DD2 600 MHz at 298 K unless otherwise specified. Chemical shifts are reported in ppm on the δ scale and referenced to the appropriate solvent peak. MeOD and CDCl_3_ contain H_2_O. HRMS analyses were carried out at the Monash University Mass Spectrometry Facility on an Agilent 6224 TOF LC/MS Mass Spectrometer coupled to an Agilent 1290 Infinity (Agilent, Palo Alto, CA). All data were acquired and reference mass corrected via a dual‐spray electrospray ionisation (ESI) source. LCMS were recorded on an Agilent LCMS system composed of an Agilent G6120B Mass Detector, 1260 Infinity G1312B Binary pump, 1260 Infinity G1367E HiPALS autosampler and 1260 Infinity G4212B Diode Array Detector. Conditions for LCMS were as follows, column: Poroshell 120 EC-C18, 2.1 x 50 mm 2.7 Micron at 20 C, injection volume 2 μL, gradient: 5–100% B over 3 min (solvent A: water 0.1% formic acid; solvent B: acetonitrile 0.1% formic acid), flow rate: 0.8 mL/min, detection: 254 nm, acquisition time: 5 min. HPLC conditions used to assess purity of final compounds were as follows, column: Phenomenex Gemini C18, 2.0 x 50 mm; injection volume 20 μL; gradient: 0–100% Buffer B over 6 min (buffer A: 0.1% formic acid in autoclaved MilliQ water; buffer B: 0.1% formic acid in 100% acetonitrile), flow rate: 1.0 mL/min, detection: 214 or 224 nm.

**Synthesis strategy for WEHI-7326 and intermediates (Related to Figure 1)**

*The medicinal chemistry strategy, starting from Naamidine-A, and the chemical synthesis methods employed to generate intermediate compounds leading to the final synthesis of WEHI-7326*

*Development of WEHI-7326:* The preparation of naamidine A and derivatives has been reported previously^4^, as well as the synthesis of early stage imidazolidine-phenoxytriazines **1’/1’’** and imidazolidine-aminotriazine compounds **4-6**^5^.

Naamidine A was not an inhibitor of the EGFR, but it blocked cells in G1 and induced apoptosis^4^. The imidaxole core and the 5-Amino-3-methylimidazolidine-e, 4-dione suggested drug-like activity. We decided to convert the imidazole core to a triazine core, and as outlined in^5^, starting with cyanuric chloride we designed and synthesized a triazine derivative with a hydroxy-phenyl- and a methoxy-phenyl group attached via amino links (**1**) or with two methoxyphenyl groups attached *via* two methylamino bridges (**6**); in both cases a 5-amino-3-methyl-imidazolidine-2,4-dione was linked to the triazine *via* its 5-amino group:

Finally, we replaced the 5-amino-3-methyl-imidazolidine-2,4-dione with 2-amino-imidazolidine-2,4-dione to produce compound **6** with significantly increased cell cycle inhibitory potency. To improve solubility and to allow for more flexibility for producing compounds with improved pharmacokinetics or for use as high-affinity ligands suitable for identifying and purifying the target, one of the methoxyphenyl groups was converted to a benzylamino group to give our lead compound **1 (WEHI-7326)**.

**Synthetic method for the preparation of compound 6 and WEHI-7326**

The synthetic preparation of WEHI-7326 and derivatives follows a reported method^6,7^.

**1-((4,6-dichloro-1,3,5-triazin-2-yl)amino)imidazolidine-2,4-di­one (7):** To a mixture of aminohydantoin hydrochloride (3.0 g, 17 mmol, 0.9 eq.) and NaHCO_3_ (3.1 g, 37 mmol, 2.0 eq.) in acetonitrile (50 mL) was added cyanuric chloride (2.8 g, 19 mmol, 1.0 eq.) at 0 °C. The reaction mixture was stirred and allowed to reach room temperature overnight. The solvent was removed *in vacuo* and the residue partitioned between ethyl acetate (100 mL) and water (50 mL). The aqueous layer was extracted with ethyl acetate (2 x 100 mL) and the combined organic layers were washed with brine and dried over MgSO_4_. After removal of the solvent *in vacuo*, the title compound was obtained as an off-white solid (2.4 g, 13 mmol, 80%), which was used in the next step without further purification. **^1^H NMR** (600 MHz, DMSO-d_6_) δ 11.43 (s, 1H), 11.24 (s, 1H), 4.16 (s, 2H). **LCMS** (ES^+^), *m/z* 263.0 (M + H).

**1-((4,6-bis((4-methoxybenzyl)amino)-1,3,5-triazin-2-yl)amino)imidazolidine-2,4-dione (6)**. To a stirred suspension of the dichlorotriazine **7** from part a) (770 mg, 2.93 mmol) and potassium carbonate (2.42 g, 17.6 mmol) in acetonitrile (20 mL) at room temperature was added 4-methoxybenzylamine (0.96 mL, 7.32 mmol). The reaction mixture was stirred for 24 h at room temperature under an atmosphere of nitrogen and then the solvent was removed under reduced pressure. The residue was extracted with ethyl acetate and the ethyl acetate was dried (MgSO_4_) and evaporated to give the crude product which was purified by flash chromatography on silica using CH_2_Cl_2_/MeOH (50:1 to 10:1) as eluent. The pure product was isolated as a colourless solid (587 mg, 43%). **^1^H NMR (d_6_-DMSO)**: *δ* 3.67 (s, 6H); 3.96 and 4.05 (2s, total 2H); 4.20 and 4.32 (2s, total 4H); 6.80 (bs, 4H); 7.13-7.39 (m, 6H); 8.67, 8.85 and 8.99 (2s, total 1H); 11.0 (bs, 1H). **MS (ES+):** m/z 465 (M+H). HRMS found: [M + H]^+^ 465.1987; C22H25N8O4 requires [M + H]^+^, 465.1993.

**4-((tritylamino)methyl)benzonitrile (8)**: To a mixture of 4‑(aminomethyl)­benzonitrile hydrochloride (1.0 g, 6.0 mmol, 1.0 eq.), trityl chloride (1.7 g, 6.0 mmol, 1.0 eq.) and DMAP (20 mg, 0.2 mmol, 0.03 eq.) in CH_2_Cl_2_ (80 mL) was added triethylamine (2.5 mL, 18 mmol, 3.0 eq.) and the resulting solution was stirred at room temperature for 16 h. The reaction was quenched by addition of water (40 mL) and extracted with ethyl acetate (3 x 100 mL). The combined organic layers were washed with brine and dried over MgSO_4_. After removal of the solvent *in vacuo*, the crude product was purified by recrystallization in ethyl acetate to obtain the title compound as an off-white solid (1.7 g, 4.4 mmol, 75%), which was directly engaged in the next step. **^1^H NMR** (600 MHz, CDCl_3_) δ 7.61 (d, *J* = 7.8 Hz, 2H), 7.56 – 7.48 (m, 8H), 7.34 – 7.27 (m, 7H), 7.23 (t, *J* = 7.3 Hz, 3H), 3.43 (s, 2H). **LCMS** (ES^+^), *m/z* 375.2 (M + H).

**N-(4-(aminomethyl)benzyl)-1,1,1-triphenylmethanamine (9)** To a vigorously stirring solution of nitrile **8** (6.0 g, 16 mmol, 1.0 eq.) in dry THF (150 mL) was added LiAlH_4_ (3.2 g, 80 mmol, 5.0 eq.) portionwise at 0 °C. The reaction mixture was stirred and allowed to reach room temperature overnight. The reaction vessel was then cooled on an ice/water bath and water (3 mL) and NaOH (2M, 3 mL) were added cautiously. The resulting suspension was filtered over celite plug and washed with THF. The filtrate was concentrated *in vacuo* and the residue partitioned between CH_2_Cl_2_ and water. The aqueous layer was extracted twice with CH_2_Cl_2_ and the combined organic layers were washed with brine and dried over MgSO_4_. The title compound was obtained as a colorless oil (5.8 g, 15 mmol, 95%), which was used in the next step without further purification. **^1^H NMR** (600 MHz, CDCl_3_) δ δ 7.57 – 7.51 (m, 6H), 7.38 – 7.34 (m, 2H), 7.32 – 7.27 (m, 8H), 7.20 (t, *J* = 7.3 Hz, 3H), 3.88 (s, 2H), 3.30 (s, 2H). **LCMS** (ES^+^), *m/z* 379.2 (M + H).

**1-((4-chloro-6-((4-((tritylamino)methyl)benzyl)amino)-1,3,5-triazin-2-yl)amino)imidazolidine-2,4-dione (10):** A mixture of dichlorotriazine **7** (1.6 g, 6 mmol, 1.1 eq.), benzylamine **9** (2.0 g, 5.4 mmol, 1.0 eq.) and K_2_CO_3_ (1.6 g, 12 mmol, 2.2 eq.) in dry acetonitrile (120 mL) was stirred vigorously at room temperature for 16 h. The solvent was removed *in vacuo* and the residue partitioned between ethyl acetate (100 mL) and water (50 mL). The aqueous layer was extracted with ethyl acetate (2 x 100 mL) and the combined organic layers were washed with brine and dried over MgSO_4_. After removal of the solvent *in vacuo*, the title compound was purified by column chromatography (silica gel; eluent: cyclohexanes/ethyl acetate = 1/1) and obtained as an off-white solid (2.6 g, 4.4 mmol, 81%). **^1^H NMR** (600 MHz, CDCl_3_) δ 8.95 – 8.63 (m, 1H), 7.52 (dd, *J* = 19.7, 7.7 Hz, 6H), 7.44 – 7.25 (m, 9H), 7.25 – 7.16 (m, 4H), 7.14 – 7.04 (m, 2H), 4.60 – 4.39 (m, 2H), 4.22 – 3.89 (m, 2H), 3.31 (s, 2H). **LCMS** (ES^+^), *m/z* 605.2 (M + H).

To a stirred solution of the chlorotriazine (**10**) (1 eq.) in anhydrous dimethylformamide (5 mL per 100 mg starting material) in an oven‑dried round bottom flask under N_2_ was added K_2_CO_3_ (3 eq.), followed by the corresponding 4-methoxybenzylamine (1.5 eq.) and the reaction was stirred under N_2_ at 80 °C for 16 h. After this time, the solvent was removed in vacuo and the residue was partitioned between ethyl acetate and water. The aqueous layer was extracted twice with ethyl acetate and the combined organic layers were washed with brine and dried over MgSO_4_. The crude product was purified by flash column chromatography (silica, eluent: cyclohexanes/ethyl acetate) to obtain the trisubstituted triazine (**11**) bearing a trityl protecting group on the terminal benzylamine moiety.

To obtain the final deprotected benzylamine **1** (WEHI-7326), the trityl protected triazine (**11**) was treated with HCl (4N) in dioxane (2 mL per 100 mg starting material) under N_2_ atmosphere at 50 °C for 5 h. During this time, a precipitate formed, which was collected by filtration and washed with diethyl ether several times. The final product W7326 was obtained as its hydrochloride salt.

**1-((4-((4-(aminomethyl)benzyl)amino)-6-((4-meth­oxybenzyl)amino)-1,3,5-triazin-2-yl)amino)imid­azolidine-2,4-dione dihydrochloride (1) (WEHI-7326)** Following above procedure using 4-(methoxy)benzylamine (1.5 eq.), the title compound was obtained as an off-white solid (172 mg, 0.37 mmol, 74%). **^1^H NMR** (600 MHz, CD_3_OD) δ 7.49 – 7.14 (m, 6H), 6.92 – 6.80 (m, 2H), 4.69 – 4.44 (m, 4H), 4.14 – 3.89 (m, 4H), 3.78 (s, 3H). **LCMS** (ES^+^), *m/z* 464.2 (M + H). HRMS found: [M + H]^+^ 464.2161; C22H26N9O3 requires [M + H]^+^, 464.2153

**References**

1 Bindels, D. S. *et al.* mScarlet: a bright monomeric red fluorescent protein for cellular imaging. *Nat Methods* **14**, 53-56, (2017).

2 Reya, T. *et al.* A role for Wnt signalling in self-renewal of haematopoietic stem cells. *Nature* **423**, 409-414, (2003).

3 Ma, H. T. & Poon, R. Y. Synchronization of HeLa cells. *Methods Mol Biol* **761**, 151-161, (2011).

4 Aberle, N., Catimel, J., Nice, E. C. & Watson, K. G. Synthesis and biological evaluation of analogues of the anti-tumor alkaloid naamidine A. *Bioorg Med Chem Lett* **17**, 3741-3744, (2007).

5 Witchard, H. & Watson, K. G. Synthesis of 5-Amino-3-methylimidazolidine-2,4-dione and 1,3,5-Triazine Derivatives as Analogues of the Alkaloids Naamidine A and G. *Thieme eJournals-Synthesis* **24**, 4312-4316 (2010).

6 Burgess, A. W., Walker, F., Watson, K. G., Witchard, H. & Lessene, G. Novel anti-cancer agents. WO/2012/054978 (2012).

7 Stevenson, G. I. Triazine compounds, compositions and synthesis. WO 2016070241 (2016).
